# Maternity healthcare professionals’ experiences of supporting women in decision-making for labour and birth: a qualitative study

**DOI:** 10.1136/bmjopen-2023-080961

**Published:** 2024-04-28

**Authors:** Kitty Hardman, Anna Davies, Andrew Demetri, Gemma Clayton, Danya Bakhbakhi, Katherine Birchenall, Sonia Barnfield, Abigail Fraser, Christy Burden, Sheelagh McGuinness, Rachel Miller, Abi Merriel

**Affiliations:** 1 Centre for Academic Women's Health, University of Bristol, Bristol, UK; 2 North Bristol NHS Trust, Westbury on Trym, UK; 3 Centre for Academic Child Health, University of Bristol, Bristol, UK; 4 Faculty of Health Sciences, University of Bristol, Bristol, UK; 5 University of Bristol Law School, Bristol, UK; 6 Independent patient representative, Bristol, UK; 7 Institute of Life Course and Medical Sciences, Department of Women's and Children's Health, Centre for Women's Health Research, University of Liverpool, Liverpool, UK

**Keywords:** Decision Making, OBSTETRICS, QUALITATIVE RESEARCH, Clinical Decision-Making

## Abstract

**Objectives:**

To explore and characterise maternity healthcare professionals’ (MHCPs) experience and practice of shared decision-making (SDM), to inform policy, research and practice development.

**Design:**

Qualitative focus group study.

**Setting:**

Large Maternity Unit in the Southwest of England.

**Participants:**

MHCPs who give information relating to clinical procedures and pregnancy care relating to labour and birth and are directly involved in decision-making conversations were purposively sampled to ensure representation across MHCP groups.

**Data collection:**

A semistructured topic guide was used.

**Data analysis:**

Reflexive thematic analysis was undertaken.

**Results:**

Seven focus groups were conducted, comprising a total of 24 participants (3–5 per group). Two themes were developed: contextualising decision-making and controversies in current decision-making. Contextual factors that influenced decision-making practices included lack of time and challenges faced in intrapartum care. MHCPs reported variation in how they approach decision-making conversations and asked for more training on how to consistently achieve SDM. There were communication challenges with women who did not speak English. Three controversies were explored: the role of prior clinical experience, the validity of informed consent when women were in pain and during life-threatening emergencies and instances where women declined medical advice.

**Conclusions:**

We found that MHCPs are committed to SDM but need better support to deliver it. Structured processes including Core Information Sets, communication skills training and decision support aids may help to consistently deliver SDM in maternity care.

**STRENGTHS AND LIMITATIONS OF THIS STUDY**

- Multidisciplinary perspective: community, integrated care, diabetes specialist, birth centre and delivery suite midwives, consultant and trainee obstetricians and specialist associate and consultant anaesthetists.
- Moderated focus group study design enabling generation of rich data.
- Online setting allowing safe collection of data during COVID-19 pandemic.
- Limited to single healthcare trust.
- Limited to maternity healthcare professionals’ perspectives.

## Introduction

Shared decision-making (SDM) is fundamental to clinical practice in obstetrics.^1–6^ It is a process where the woman is at the centre of her care and is able to share information regarding decision-making preferences, personal values and beliefs, and where the maternity healthcare professional (MHCP) provides information about benefits and risks of management options to enable an autonomous, informed decision.^4 5 7^ Informed consent (IC), often the endpoint of SDM, is where the woman makes an informed, voluntary choice of treatment and is often symbolised by signing a consent form.^3 8^ MHCPs are legally bound to achieve IC prior to providing treatment.^4 8^

Various terminology has been used to describe decision-making practices, including SDM, informed decision-making and supported decision-making.^5 9^ This research follows UK General Medical Council (GMC), National Health Service (NHS) and Royal College of Obstetricians and Gynaecologists guidance on decision-making and consent,^4 10 11^ and therefore, uses the term SDM to ensure understanding and comparison. However, it is recognised that the word ‘shared’ may fail to acknowledge women as the ultimate decision-makers.^12 13^ We have referred to ‘woman/women.’ Other parents and families use different words and we respect their chosen terminology. An ongoing dialogue between healthcare systems and the patients they serve is required to ensure patient-centred terminology.

SDM is an international healthcare priority.^3 14 15^ It provides short-term and long-term benefits through improved birth experiences, satisfaction with care regardless of outcome, improved maternal mental health outcomes, reduced preterm birth, higher birth weights and enables safer care.^16–20^ Failing to involve women can lead to their feeling out of control and powerless and is associated with negative and traumatic birth experiences, increased rates of postnatal depression, anxiety and PTSD.^19 21^

Decision-making occurs throughout pregnancy. However, achieving intrapartum and emergency SDM poses unique challenges: women may be in pain, tired, scared, under the influence of opiate analgesia or all of the above.^22 23^ In addition, they often have limited time to consider available options and the risks posed to themselves or their baby.^22^ Frequently cited barriers to practising SDM are time pressures and lack of clinical applicability, that is, a belief that SDM is inappropriate in that clinical situation.^24^

Despite these challenges, the Royal Colleges of Emergency Medicine, Obstetrics and Gynaecology and Midwifery provide limited emergency-specific SDM guidance.^5 7 25^ The GMC advises taking a proportional approach to emergency decision-making, leaving MHCPs to subjectively interpret best practice. Given limited guidance and challenges posed by emergency care, it is unsurprising that emergency obstetric interventions confer the greatest sense of perceived loss of control and choice, and are associated with the poorest psychosocial outcomes.^26 27^

Guidance is needed for SDM in maternity, and especially intrapartum care, to achieve better psychosocial outcomes for women and to support MHCPs.^17^ We aimed to understand MCHP’s experience of decision-making from a multidisciplinary perspective in maternity care as a foundation to develop interventions to improve practice.

## Methods

The Standards for Reporting Qualitative Research checklist guided reporting of this study^28^ (online supplemental file S1).

10.1136/bmjopen-2023-080961.supp1 Supplementary data

### Patient and public involvement

A patient representative was a member of the project steering committee and contributed to protocol design.

### Research team and reflexivity

The research team comprised obstetricians (KH, ADe, DB, KB, SB, CB and AM), a research psychologist (ADa), patient representative (RM), a lawyer (SM), information specialist (KB) and epidemiologist (AF). Data collection and analysis was carried out by KH, ADe, ADa and AM. KH and ADe are trainee obstetricians and early career academics, ADa, a research psychologist, with qualitative and maternity research experience, AM is an academic obstetrician. While the professional background of the researchers (KH and ADe) enabled ‘fitting in’ with participants,^29^ we considered possible over-representation of the MHCP perspective; therefore, a second non-MHCP moderator (ADa) attended focus groups and multidisciplinary discussion of candidate themes was undertaken by the research team.

### Study design

Moderated focus groups explored MHCPs’ experiences of SDM. Focus groups provide an open, supportive environment that facilitates in-depth discussions about sensitive and personal topics, leading to new and unexpected knowledge.^30^

### Participant selection, sampling and sample size

Participants were purposively sampled from a single NHS trust in the south-west of England, with approximately 6000 deliveries annually. All MHCPs directly involved in clinical decision-making conversations with women were considered for inclusion. We targeted midwives (community, integrated care, birth centre and delivery suite) and doctors (consultant and trainee obstetricians and anaesthetists). Focus groups included between three and five participants to ensure each person had the opportunity to contribute. Potential participants were approached via email, posters and word of mouth. Participant information leaflets were emailed to interested participants and remote IC, demographic data, and anonymised record ID numbers were generated and recorded using REDCap.^31 32^ Participants received a £10 e-voucher. Recruitment continued until data was said to be ‘saturated’.^33^

### Data collection

Moderated focus groups were held online in July 2021.The primary moderator (KH) asked questions, while the second moderator (ADe/ADa) took field notes. A topic guide (online supplemental file S2) structured the discussions but once they have begun, the natural flow was not interrupted. Questions relating to experiences and challenges of supporting women in decision-making, perceptions of maternal preparedness for labour and birth, and ways in which decision-making for labour and birth could be improved were asked. An encrypted audio recording device was used, audio recordings were transcribed verbatim and uploaded into NVivo.^34^

10.1136/bmjopen-2023-080961.supp2 Supplementary data

### Data analysis

An experiential orientation to data interpretation was adopted. Meaning was derived through personal experiences, and how individuals process these experiences.^35^ Reflexive thematic analysis was undertaken using an iterative process (online supplemental file S3).^36 37^ An inductive (bottom-up) approach was taken, whereby codes and themes were directly linked to the data, however, a degree of deductive (top-down) analysis was used to ensure the research question remained at the fore.^37 38^ Each phase was carried out independently and then discussed collaboratively (KH, ADa and AM).

10.1136/bmjopen-2023-080961.supp3 Supplementary data

### Trustworthiness

The presence of a second moderator through all focus groups enabled field notes to be taken throughout. The re-reading of these notes alongside audio recordings ensured non-verbal communication, and subtleties of communication were captured enabling an accurate interpretation of what was said and triangulation of data sources.

After each focus group, moderators would reflect on how the focus group had run and discuss initial interpretation of the data, enabling immediate assumptions to be challenged and discussed. A continuous and ‘prolonged engagement’ of reflection and discussion was maintained throughout the study^39 40^(online supplemental file S3). Data saturation was reached once researchers independently agreed that sufficient ‘thick’ and ‘rich’ data had been achieved, and no new codes or themes emerged through group discussion.^40^

## Results

Seven focus groups, each with 3–5 participants per group (24 participants in total) were conducted in July 2021 (see table 1). Participants included community (CMW), integrated care and diabetes specialist midwives (IMW), birth centre (BCMW) and delivery suite (DSMW) midwives, trainee (TO) and consultant obstetricians (CO) and specialist associate and consultant anaesthetists (A).

**Table 1:** Focus groups by participants and experience in maternity care

Focus group	Participants in group	Participants (n)	Experience in maternity care (years)
1	Midwife, community and integrated care	3	5–35
2	Midwife, hospital working in birth centre	3	6.5–17
3	Anaesthetist, consultant and associated specialist	3	12–30
4	Midwife, community, integrated care and diabetes specialist	3	4–30
5	Midwife, hospital working in delivery suite	3	13–19
6	Obstetrician and gynaecologist, consultant	5	11–20
7	Obstetrician and gynaecologist, trainee	4	3.5–9
Total		24	

**Figure 1:**
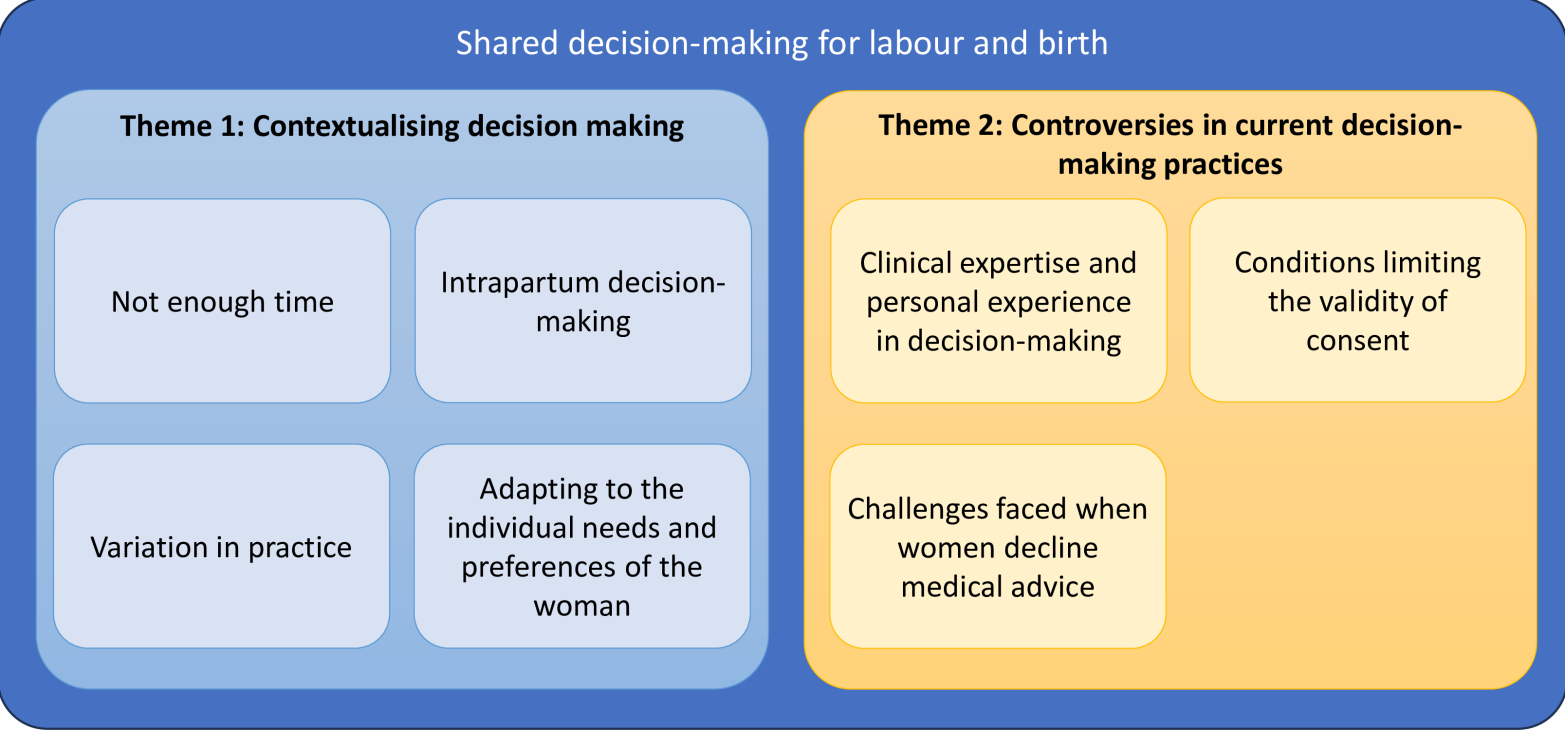
Themes and subthemes.

Two overarching themes were developed: theme 1: contextualising decision-making and theme 2: controversies in current decision-making practices. Figure 1 illustrates each theme and component subthemes. Select quotations supporting each theme are presented in boxes 1 and 2, with full list of quotations in online supplemental files S4,S5. Each theme and subtheme will be discussed in turn.

10.1136/bmjopen-2023-080961.supp4 Supplementary data

10.1136/bmjopen-2023-080961.supp5 Supplementary data

Box 1 **Theme 1 : Contextualising decision-making.**

**Subtheme 1.1: Not enough time**

I keep coming back to time…what I keep coming back to, but you know, it's time to process. Process that information, and then come to as [name] said, you know, what might not necessarily be what we think is the right decision, from our perspective, but when it comes to the patient, and you're bringing all that information together, they feel that's the right decision for them. TO, P22

The midwives that we all work with are incredibly stressed, underfunded, under great time pressures, and there are not enough of them to do the work that is required and the population is increasing and their workload is increasing. TO, P23.

And I feel like shared decision making is something that we all aspire to in situations where we as clinicians feel that there is enough time.TO, P24.

**Subtheme 1.2: Intrapartum decision-making**

There are times that it isn't always possible to give them …accommodate them having a discussion about something sometimes you do have to make…more channelled decision making. BCMW, P5.

You have to we, we have to, and also the birth educators that we currently have in this country have to start having conversation from the very first antenatal class that they hold. BCMW, P6.

At the point where they're in… the process of the labour…that too much choice at that point is actually really derailing. And then I felt like I've left conversations thinking, why did I even? Why did I even do that to that poor woman? Like she's now on the edge to a really traumatic experience, because I've given her those choices and tried to say, look, there are other ways you can do X, Y, and Z. BCMW, P6.

**Subtheme 1.3: Variation in practice**

I think there's just such a massive variety of sources of information that women receive and I don't think there's a huge amount of standardisation. TO, P22.

So I think every single woman you tailor what you say differently. It's all according to like you say what, or how, or your perception of their understanding as well. IMW, P12.

There are days when you're better at it, then there are days when you think, ‘Oh, God, I could have done that better.’ CMW, P11. Communication is something that we bang on about all the time and you do it, you know…everyone's saying ‘you know communication's key’, but actually, the communication isn't always there. IMW, P3.

**Subtheme 1.4: Adapting to the individual needs and preferences of the woman**

Whilst we want to give women this information, to try and empower them, and hopefully make things better, that I think there will be a group of patients who who will, they won't want that information, because they'll find it potentially very scary, or, you know that, but certainly, it might put some barriers up to accepting that information. TO, P22.

I think using an interpreter for people with a language barrier has a profound impact on trying to communicate in an emergency or even semi emergency situation. If I have someone on the labour ward who in any way might need a caesarean, sometimes in the middle of night, I find it quite useful to go in and go through a consent form with a translator in advance of doing a procedure because I think for those women communicating with them is so incredibly difficult. TO, P21.

You've got the whole range of the tertiary level educated patient who doesn't want us to do anything versus quite often someone who maybe left school after GCSEs…but you still have to provide both of those sets of patients with all the same information, but you have to then guide how you do that. And that's, that can be quite challenging. TO, P22.

Box 2 **Theme 2: Controversies in current decision-making practices.**

**Subtheme 2.1: Clinical expertise and personal experience in decision-making**

So what you would tend to do in that situation is probably stress the downsides of having a general anaesthetic and talk about actually, you know, failed intubation…So actually, we will manipulate that conversation based on us…thinking we actually probably do know the best thing for that patient. A, P8.

We do all subconsciously do that, we select which bits of information we think the patient needs. A, P9.

I sort of feel like women are very coerced…And I feel like the information that’s shared with women isn't neutral. They're scared into stuff. CMW, P12.

[The BRAIN app] is really good because it gives a really good balance and what are the risks, what are the benefits, what are the alternatives, what are the family’s preferences. So it just it’s a really good tool for facilitating those shared decisions, and looking at other people’s perspectives as well. CMW, P2.

**Subtheme 2.2: Conditions limiting validity of consent**

I think in an emergency situation, I find it very difficult, because I think the consent process I currently go through seems like a bit of a sham… we go through this process of waving a consent form at them saying *‘you and your baby going to die. If we don't do this*’. TO, P21.

When you're trying to consent a labouring woman for an epidural, and she’s screaming, *‘just put it in*’ at you that, you know, they don't take on board, we could tell them the risks were, you know, 1 in 2 risk of death or something, at that point, they're not listening to you at all. A, P8.

We cannot say that a woman in labour is giving true consent, even for an epidural, when she has so much pain…She’s so crippled and tired and, you know, fed up with everything, that she'll just agree to anything. IMW, P12.

I’ve never seen anyone try and do a decision making kind of conversation at the time of a shoulder dystocia, and I've also never come across a mum who has retrospectively said, *‘I can't believe you didn't talk to me about that first*.’ TO, P24.

**Subtheme 2.3: Challenges faced when women decline medical advice**

She knew the risk, but she was absolutely clear what the risks were, what the implications could be what the outcome could be for her baby, but, that was the decision that she wanted. And it’s it was so difficult. IMW, P3.

I think it is the fear of, of litigation, and that defensive practice, which is the overwhelming you know, feeling. I know, I've had some personal experiences around that. So that definitely does probably change the way I practice as a midwife, making me perhaps more overcautious…it’s that kind of fear of, if something happens or goes wrong the responsibility then lies with you as the midwife, and the woman…will turn around and say, *‘Well, that was something that you didn't do,*’ Or *‘if you'd have told me something differently, that wouldn't have happened*.’ BCMW, P5.

It’s like women who decline induction, it’s like, well, we'll tell you about the risks again, because you aren't doing what we've decided is the right thing to do from our perspective of, you know, recommendations. *‘Remember, it’s on you now*’…You know, and therefore, it’s not shared decision making. BCMW, P6.

That might be because we've alienated people as well. So I think with with regards to pre birthing, and birthing outside of guidance. CO, P19.

They were quite bullish, actually in the hospital, they kept ringing her but she just turned the phone off in the end and said, *‘I'm not going to speak to you, I need a day off from all of you*’. CMW, P11.

### Theme 1: contextualising decision-making

Participants identified systemic barriers to SDM. They felt there was not enough time to adequately discuss management options and described limiting the discussion to the time available. Participants felt more time could enable better discussions. MHCPs reported significant variation in their individual approach to SDM. Decision-making conversations varied depending on the individual needs and preferences of the woman.

#### Subtheme 1.1: not enough time

All groups felt their ability to achieve SDM was related to the time available, ‘I keep coming back to time… when you've got that time to actually provide some information’ (TO, P22).

Having time enabled MHCPs to perform high-quality decision-making, which involved exploring preferences, addressing fears and building trusting relationships. However, the time routinely available was felt to be insufficient, ‘you are very much trying to limit the consultation based on the time that you're given for that woman. So I would say that when I'm allowed longer time with a woman I would think it was a more informed decision that was going to come out of that because I have time to listen’ (CO, P17).

Experienced community midwives felt that systemic changes including reduced appointment times contributed to poorer SDM, ‘we used to spend hours sitting with every single woman before she delivered doing the birth plan, but it wasn't really the birth plan, it was a birth discussion…so she could tell you all her fears, and that would help with her decision-making process’ (IMW, P1).

Participants described good decision-making experiences to involve multiple or longer appointments to build rapport, so women could process information and deliberate decisions, however, this was not the norm. For example, when a CMW described using multiple longer antenatal appointments another participant replied, ‘So great [name] that you've managed to find space for someone, in a quite a complex situation, but for the *majority* of women, it’s a very superficial process… So we really need to improve that for, you know, for every woman’ (IMW, P3).

#### Subtheme 1.2: intrapartum decision-making

SDM during labour was challenging, women were felt to have limited capacity to engage in conversations due to pain, fatigue and feeling scared and that decisions were time-dependent, ‘you've not got time to just pause the body, give everyone a break, give the woman 25 minutes to process the information’ (BSMW, P6). Presenting women with new information and multiple options during labour was percieved to be potentially ‘derailing’ and ‘traumatising’. SDM approaches adopted in the antenatal setting were sometimes felt to be ineffective in achieving a meaningful, informed conversation.

Groups emphasised the need for improved antenatal education so that women arrived at the point of birth informed of the main options, and knowledgeable of their preferences, so that discussions in labour did not require giving new information, or unexpected choices.

#### Subtheme 1.3: variation in practice

Decision-making practice varied within and between MHCPs. Communication skills and how information was imparted to women varied with time, the decision, the patient and the clinician, ‘every single situation, every woman is different, every doctor is different, every interaction is different. All you can do is keep honing your skills, practising and doing your best. [There] definitely isn't one way of doing it’… (A, P8).

All groups described an ad hoc approach to developing communication skills for decision-making, with none receiving formal training. The obstetrician and anaesthetist groups discussed the importance of learning from senior clinicians, ‘seeing how different people do these things in order to work out actually what would work for our particular communication style or personality to try and keep things as, as shared and as broad as possible’ (TO, 22). The midwifery groups reported fewer on-the-job learning opportunities. Participants felt communication skills training would improve consultations.

All participants reported variation in their ability to achieve SDM, ‘there are days when you're better at it, then there are days when you think, *‘Oh, God, I could have done that better*’ (CMW, P11). Factors such as fatigue, hunger and stress were felt to also contribute to how well they carried out SDM, ‘sometimes women get less a whole lot less from me, than perhaps they should because I'm tired and rushed’ (A, P9).

### Subtheme 1.4: adapting to the individual needs and preferences of the woman

There was perceived variation in the individual needs and decision-making preferences of women. Most notably, women who did not speak English faced barriers to communication, and pre-emptive conversations in these situations were felt to be essential to ensure understanding and consent, ‘I think using an interpreter for people with a language barrier has a profound impact on trying to communicate in an emergency or even semi emergency situation…I find it quite useful to go in and go through a consent form with a translator in advance of doing a procedure because I think for those women communicating with them is so incredibly difficult’ (TO, P21).

Others highlighted the challenges of ensuring patients from all sociodemographic backgrounds were equally informed, ‘you've got the whole range of the tertiary level educated patient who doesn't want us to do anything vs quite often someone who maybe left school after GCSEs…but you still have to provide both of those sets of patients with all the same information…that can be quite challenging’ (TO, P22).

Lastly, women were perceived to vary in the amount of information they wanted to receive, and their role in decision-making, some seeming to prefer clinician-led decision making, ‘You know, there are some people who say, *‘I don't want to know, I don't want to know the risks of this, I just want you to do what you think I need*…but I’ve got to tell you…it says here I have to consent…I have to tell you all the risks, and they don't want to know’ (A, P8).

### Theme 2: controversies in current decision-making practices

Theme 2 explores controversies in current decision-making practices: participants reported providing information in a way that aligned with their clinical perspective; pain and emergency situations were felt to limit the validity of IC; women declining medical advice was challenging and MHCPs were fearful of medicolegal repercussions, while these women were made to feel isolated.

#### Subtheme 2.1: clinical expertise and personal experience in decision-making

All groups reported bringing their clinical expertise, training and experience to decision-making conversations, resulting in women receiving differing information from different MHCPs, ‘a [midwife] describing a breech where they do it quite frequently-ish, vs like a consultant who works in HSIB [Healthcare Safety Investigation Branch] that is a very different description that you will receive’ (TO, P21).

Two distinct issues became apparent. First, the way in which MHCPs conducted decision-making conversations and the information provided to women was influenced by training, experience and individual interpretation of the available evidence, and was described by some as their personal or clinical bias. This was felt to be very difficult to mitigate.

Second, there were occasions where participants felt that they presented information differently depending on the particular clinical situation, ‘so like if I don't want to induce a patient at 37 weeks for a pretty benign reason, but the patient is really keen to be induced, I will give them the figures for NICU admission, whereas if there’s a patient who I want to induce, I might not necessarily tell them that same information’ (TO, P23). This practice was reported most among the obstetrician and anaesthetist groups.

Some participants had an insight about their potential clinical biases. They discussed the importance of using absolute rather than relative risk and infographics to help communicate information objectively. Two participants described using a decision tool to help standardise information.

#### Subtheme 2.2: conditions limiting the validity of consent

MHCPs believed that severe pain and life-threatening emergencies meant it was near-impossible to achieve IC, let alone SDM.

The anaesthetic and midwifery groups felt that many women were unable to weigh up risks and benefits of an epidural when they were in so much pain, ‘when you're trying to consent a labouring woman for an epidural, and she’s screaming, *‘just put it in!*’…we could tell them the risks were, you know, 1 in 2 risk of death or something, at that point, they're not listening to you at all’ (A, P8). The anaesthetic group felt pre-emptive conversations regarding epidural analgesia were important, and reported using information cards to support this.

All groups questioned whether SDM and IC is possible in life-threatening situations. A trainee obstetrician reported, ‘I’ve never seen anyone try and do a decision-making kind of conversation at the time of a shoulder dystocia, and I've also never come across a mum who has retrospectively said, *‘I can't believe you didn't talk to me about that first*’ (TO, P24). However, they reported following process and signing consent forms, despite feeling it doesn’t reflect SDM or IC. A trainee obstetrician reported, ‘I think in an emergency situation, I find it very difficult, because I think the consent process I currently go through seems like a bit of a sham… we go through this process of waving a consent form at them saying, *‘you and your baby going to die if we don't do this*’ (TO, P21).

#### Subtheme 2.3: challenges faced when women decline medical advice

Decision-making conversations were felt to be challenging when women decline medical advice. MHCPs were psychologically affected by poor neonatal and maternal outcomes and fearful of medicolegal repercussions, ‘it gets turned very much back against you as the medical professional saying, *Why didn't you explain that this might happen?* Even if it’s been written in black and white…’ (TO, P23).

Several participants reported practising more defensively having experienced poor neonatal outcomes. Furthermore, participants from all groups felt they needed to explain all risks to women to protect themselves against litigation, ‘it’s that kind of fear of, if something happens or goes wrong the responsibility then lies with you as the midwife, and the woman…will turn around and say, *‘Well, that was something that you didn't do,*’ Or *‘if you'd have told me something differently, that wouldn't have happened*’ (BCMW, P5).

However, this approach was perceived to infringe on women’s experience of decision-making. For example, women were reportedly harassed when they declined medical advice and made to feel that their decisions were not respected by repeatedly being told the risks of declining medical advice, or being repeatedly offered medical interventions. A community midwife described a woman having to turn her phone off to avoid repeated phone calls offering her an induction of labour.

## Discussion

In this qualitative exploration of MHCPs’ experiences of decision-making, participants were motivated to involve women in decision-making. However, challenges to SDM included time pressures, lack of training and intrapartum/emergency care. MHCPs perceived that women’s decision-making needs and preferences varied, non-English-speaking women faced communication challenges. Suggested changes to improve SDM were increased consultation time, skills training and improved antenatal education. Three areas of controversy were explored the role of prior clinical experience in SDM, the validity of IC during intrapartum/emergency care and when women declined medical advice.

The need to deliver patient-centred care, with time to ask questions, express concerns and receive high-quality information coincides with increasing demands on healthcare systems.^3–5 41^ A systematic review of barriers and facilitators to SDM found that time constraints are the most commonly cited barrier across cultural and organisational contexts.^24^ For SDM to be successfully implemented a systems approach needs to be considered to provide clinicians with time and resources to counsel women.^15^

Decision aids can support MHCPs to standardise content, support risk communication, facilitate discussion about what matters patients, and reduce decisional conflict without extending consultations.^42–44^ In UK maternity care, the use of decision aids is growing with tools to provide decision-making structure,^10^ support discussion about mode of birth^45^ and intrapartum decision-making.^46^

However, it is unlikely that there can be a decision aid for every decision, and they are not universally acceptable or useful.^47^ One effective way of improving decision-making skills for clinicians is to role play different decision options alongside the integration of decision aids.^47 48^ The NHS personalised care plan expects clinicians to be trained in decision-making conversations,^15^ however, none of our participants had formal SDM training. MHCPs need to be equipped with the tools to support SDM, and given the opportunity to attend training.

Language poses a significant barrier to SDM. Women who do not speak the local language face issues around communication and this may affect quality of care.^49 50^ The National Institute for Health and Care Excellence (NICE) emphasises the importance of using clear language with resources translated into other languages if needed.^50 51^ Our participants had developed strategies to manage decision-making in this group; it is important that the maternity system develops a strategy to support these vulnerable women.

Participants’ prior experiences influenced their communication, and in some instances, the decision chosen by the woman. These findings are in keeping with research from a range of specialities.^26 52^ MHCPs have a duty to declare personal beliefs and potential biases to ensure transparency, however, how often this happens in reality is unclear.^4 53^ The use of decision aids may help to standardise information, and free it from clinicians’ personal biases.^42^

In instances where women declined medical advice, participants expressed conflict between fear of litigation and patient autonomy. MHCPs may try to persuade women to accept medical interventions as there is a common belief that they may incur ethical or legal liability if women decline.^54 55^ However, this persuasion could negate consent as voluntary choice has been lost.^54^ MHCPs should be supported to explore the values underlying a woman’s refusal while emphasising patient choice. They should be enabled to maintain communication to facilitate safest possible care,^26^ and in doing so support women to be autonomous decision-makers, and exercise their right to informed refusal of care. Structured, informed refusal processes may help MHCPs feel more confident in caring for these patients and prevent women from feeling ostracised from medical care.^54 56^

Participants questioned the validity of consent when women were in pain and during emergencies. Procedures relating to IC were perceived to become meaningless paperwork rather than respectful support and autonomy. Women who consented in an emergency are more likely to feel that they would have signed whatever was on the consent form, find the consent form harder to understand and are less likely to remember signing it, and their overall satisfaction with the consent process is lower.^57^ Focusing on obtaining written consent in emergency scenarios may not achieve either informed choice or woman-centred care.^7^ Better birth preparation may improve this.

Participants suggested that presenting new information in labour can be overwhelming. While one cannot legislate for every eventuality, women should be aware of common obstetric interventions.^58^ Improving antenatal education and preparation for birth is vital to improving birth experiences,^19 54 59^ and should be consistently delivered throughout pregnancy to enable SDM.^10 53^ The development of Core Information Sets regarding vaginal birth, unplanned assisted birth and unplanned emergency caesarean births offer one way which may help women to receive consistent, accurate information, that is valued by them,^60–62^ while the use of decision aids may help to standardise and guide decision-making conversations.^42^

### Strengths and limitations

Further research could involve participant recruitment from additional healthcare trusts and geographically and socially diverse areas. However, our findings are congruent with decision-making experiences across maternity settings, suggesting these results may be relevant more broadly.

The online focus groups enabled the study to proceed during the COVID-19 pandemic, they created a relaxed atmosphere and enabled open discussion.^30^ However, technical issues introduced additional challenges. Gaining perspectives of women’s experience of SDM is essential and work undertaken to address this is currently being analysed.

## Conclusion

To improve women’s birth experiences and to better support MHCPs, a systems-wide approach to SDM must be considered. Women require access to information and support throughout pregnancy to ensure they are prepared for decision-making in labour and birth, including familiarisation of common emergency obstetric interventions, and the possibility of unexpected choices. The development of Core Information Sets, better support tools and training for staff will help women to receive balanced information, relevant to them. MHCPs must be supported in providing advice and care to women birthing outside of guidelines with well-defined pathways for those who decline medical advice. Women must be supported to be autonomous decision-makers, including those who choose informed refusal of care. Decision-making and consent during intrapartum and emergency situations should be revisited given the concerns regarding its validity. MHCPs believe in SDM. It is important that research, training and policy mature alongside health systems to deliver SDM to all women throughout their pregnancy journey.

## Supplementary Material

Author's manuscript

## Data Availability

Data are available on reasonable request. Data are available on reasonable request and subject to relevant ethical approvals.
