# Supplementary material for: Maternity healthcare professionals’ experiences of supporting women in decision-making for labour and birth: a qualitative study
Source: BMJ Open. 2024 Apr 28;14(4):e080961. doi: 10.1136/bmjopen-2023-080961 (PMC11057275; doi:10.1136/bmjopen-2023-080961)
Supplement: Supplementary data [file bmjopen-2023-080961supp001.pdf]

Supplementary table 1, Standards for Reporting Qualitative Research (SRQR) Checklist(1)

No.	Topic	Item
**Title and abstract**		
S1	**Title**	Page 1, Rows 2-3
S2	**Abstract**	Page 2, Rows 17-51
**Introduction**		
S3	**Problem formulation**	Pages 4-5, Rows 75-87
S4	**Purpose or research question**	Page 5, Rows 87-89
**Methods**		
S5	**Qualitative approach and research paradigm**	Page 6, Rows 106-108<br>Page 7, Rows 128-132
S6	**Researcher characteristics and reflexivity**	Page 5, Rows 96-104
S7	**Context**	Pages 6-7, Rows 119-126
S8	**Sampling strategy**	Page 6, Row 109-118<br>Page 7, Rows 139-143
S9	**Ethical issues pertaining to human subjects**	Page 21, Rows 375-377
S10	**Data collection methods**	Pages 6-7, Rows 119-126
S11	**Data collection instruments and technologies**	Pages 6-7, Rows 119-126
S12	**Units of study**	Page 8, Rows 144-150
S13	**Data processing**	Page 7, Rows 124-125.
S14	**Data analysis**	Page 7, Rows 127-133, and supplementary file, S3.
S15	**Techniques to enhance trustworthiness**	Page 7, Rows 134-143

**Results/findings**		
S16	**Synthesis and interpretation**	Pages 8-16, Rows 144-283.
S17	**Links to empirical data**	Pages 8-16, Rows 144-283. Supplementary files S4, S5.
**Discussion**		
S18	**Integration with prior work, implications, transferability, and contribution(s) to the field**	Pages 17 -19, Rows 284-340
S19	**Limitations**	Page 19, Rows 341-348
**Other**		
S20	**Conflicts of interest**	Page 20, Rows 363-364
S21	**Funding**	Page21, Rows 378-382

1. O'Brien BC, Harris IB, Beckman TJ, Reed DA, Cook DA. Standards for Reporting Qualitative Research: A Synthesis of Recommendations. Academic Medicine. 2014;89(9).
