# Supplementary material for: Maternity healthcare professionals’ experiences of supporting women in decision-making for labour and birth: a qualitative study
Source: BMJ Open. 2024 Apr 28;14(4):e080961. doi: 10.1136/bmjopen-2023-080961 (PMC11057275; doi:10.1136/bmjopen-2023-080961)
Supplement: Supplementary data [file bmjopen-2023-080961supp002.pdf]

Shared decision making for labour and birth

Staff focus group questions

Intro: This study focusses on shared decision making. We're trying to understand your experiences of supporting women in their decision making.

We also want to find out about what you think is needed to optimise the support that you provide to women when making decisions.

Topic area 1:

Have you heard of shared decision making, what does it mean to you?

Prompt: exchange of information, listening, sharing, weighing, balancing

What do you think the important elements of decision making are?

Topic area 2:

Can you describe your personal experience of supporting women to make decisions?

Prompts: antenatally birth choices, ERCS vs VBAC, during labour, analgesia choices and pain management, instrumental delivery vs CS, Foetal blood sampling, birthing positions.

Topic area 3 :

Do you think women are well informed and prepared for the interventions, or medical care they might receive during labour and birth?

Appropriate expectations

Prompts: where do you think they get their information from, do you think the information they have is accurate and sufficient? What information do women receive about intrapartum interventions?

Do you think this affects their decision making ability?

Topic area 4:

How can we better support women to make decisions during labour and birth?

More training for health care professionals **,** More consultations, more antenatal classes, videos, leaflets

Topic area 5:

Which interventions, or decision choices do you think we should be focus on when practising shared decision making?

For example Which interventions are women least expecting? Least information. Most uncertainty, distress

Topic area 6:

Are there any interventions that should not involve shared decision making during labour?

Prompts: are you happy to participate in supporting shared decision making for all interventions? Life threatening emergencies are not usually expected to involve shared decision making – what is a life-threatening emergency to you?
