# Supplementary material for: Maternity healthcare professionals’ experiences of supporting women in decision-making for labour and birth: a qualitative study
Source: BMJ Open. 2024 Apr 28;14(4):e080961. doi: 10.1136/bmjopen-2023-080961 (PMC11057275; doi:10.1136/bmjopen-2023-080961)
Supplement: Supplementary data [file bmjopen-2023-080961supp003.pdf]

Supplementary Table 3, A phased approach to reflexive thematic analysis (1, 2)

	Phase	Description of activities
1	Familiarisation with data	Immersion within the data was carried out independently (KH, AKD, AM). It was achieved by reading, and re-reading the data set and referring to the field notes associated with each focus group.
2	Generating initial codes	The dataset was coded independently by KH, AKD, and AM using succinct, shorthand descriptive labels.
3	Generating themes	Through a series of meetings, KH, AKD, and AM collaboratively explored initial themes using an inductive approach, whereby themes were directly linked to the data (3). Through this approach we hoped to directly represent the lived experiences of MHCPs', rather than by attempting to fit them onto pre-defined social constructs or frameworks.<br><br>In keeping with our experiential approach meaning and meaningfulness of themes was attributed to their relevance in answering the research question, and the significance participants attributed to these issues (4, 5, 6). Whilst we were also interested in the concept of theme frequency, and commonality, we also were aware that what is common is not necessarily meaningful or important(6).
4	Reviewing and identifying themes	A process of reading and re-reading themes, codes and data was carried out to reach a set of consistent, distinct, and coherent themes. Theme discussion, reflection and exploration occurred simultaneously.
5	Defining themes	The data set and coded data items were re-read to ensure that candidate themes functioned as meaningful interpretation of the data.
6	Write up	An illustrative narrative analysis of each theme was undertaken. A final review of theme name and order was undertaken to ensure the themes and key messages reflected the data, and answer the research question in a logical and clear manner.

1. Braun V, Clarke V. Reflecting on reflexive thematic analysis. Qualitative Research in Sport, Exercise and Health. 2019;11(4):589-97.
2. Byrne D. A worked example of Braun and Clarke's approach to reflexive thematic analysis. Quality & Quantity. 2021.

3. Braun V, Clarke V. Using thematic analysis in psychology. Qualitative Research in Psychology. 2006;3(2):77-101.
4. Byrne D. A worked example of Braun and Clarke's approach to reflexive thematic analysis. Quality & Quantity. 2021;56:1391-412.
5. Clarke V, Braun V. Thematic analysis. The Journal of Positive Psychology. 2017;12(3):297-8.
6. Braun V, Clarke V. Thematic analysis. APA handbook of research methods in psychology, Vol 2: Research designs: Quantitative, qualitative, neuropsychological, and biological. APA handbooks in psychology®. Washington, DC, US: American Psychological Association; 2012. p. 57-71.
