# Supplementary material for: Maternity healthcare professionals’ experiences of supporting women in decision-making for labour and birth: a qualitative study
Source: BMJ Open. 2024 Apr 28;14(4):e080961. doi: 10.1136/bmjopen-2023-080961 (PMC11057275; doi:10.1136/bmjopen-2023-080961)
Supplement: Supplementary data [file bmjopen-2023-080961supp004.pdf]

**Supplementary Table 4. Theme 1: Contextualising decision-making**
Subtheme 1.1. Not enough time
lets explore that further, and what's concerning you, and what's led to that decision so far, so that we can make sure that it's the right decision for you. TO, P22.
So our discussion starts with "*Tell me about what you want your experience to be?...Tell me about what you're planning?"...*So it's very much a - their decision making can't happen without the information that I'm going to give them, but equally, I'm taking into account information they're giving me to help them come to a conclusion that works for them... BCMW, P4.
I keep coming back to time, maybe this is ....what I keep coming back to, but you know, it's time to process. Process that information, and then come to as [name] said, you know, what might not necessarily be what we think is the right decision, from our perspective, but when it comes to the patient, and you're bringing all that information together, they feel that's the right decision for them. TO, P22
the midwives that we all work with are incredibly stressed, underfunded, under great time pressures, and there are not enough of them to do the work that is required and the population is increasing and their workload is increasing. TO, P23.
And I feel like shared decision making is something that we all aspire to in situations where we as clinicians feel that there is enough time...TO, P24.
That's why women aren't given information, we have 20 minutes to do so many things…CMW, P11.
So great [name] that you've managed to find space for someone, in a quite a complex situation, but for the majority of women, it's a very superficial process… So we really need to improve that for, you know, for every woman. IMW, P3
you are very much trying to limit the consultation based on the time that you're given for that woman. So I would say that when I'm allowed, longer time with a woman, I would think it was a more informed decision that was going to come out of that because I have time to listen. CO, P17.
Subtheme 1.2: Intrapartum decision-making
There are times…that it isn't always possible to give them ...accommodate them having a discussion about something sometimes you do have to make more…more channelled decision making. BCMW, P5.
You have to we, we have to, and also the birth educators that we currently have in this country have to start having conversation from the very first antenatal class that they hold. BCMW, P6.
At the point where they're in… the process of the labour…that too much choice at that point is actually really derailing. And then I felt like I've left conversations thinking, why did I even? Why did I even do that to that poor woman? Like she's now on the edge to a really traumatic experience, because I've given her those choices and tried to say, look, there are other ways you can do X, Y, and Z. BCMW, P6.
How then are we expecting women to be ready to make decisions when they've actually not made a decision at all throughout the whole process of the nine months prior to that. So the whole time when they're meant to be training almost for the event of... Trying to make the shared decisions. We've not given them any training time. Instead, what you say is: *"Okay, at the point of birth, then you get choices"*. But actually, at the point of birth, the choices go from nothing to a million and one choices. BCMW, P6.
So let's prepare them for the main options, and then train them to be fluid, you know, so that then they have a slightly more open minded, kind of coming into it. BCMW, P6
Subtheme 1.3: Variation in practice
I think there's just such a massive variety of sources of information that women receive and I don't think there's a huge amount of standardisation. TO, P22
Every single situation, every woman is different. Every doctor is different, every interaction is different. All you can do is keep honing your skills, practising and doing your best. There's no, I don't think there's one way that definitely isn't one way of doing it...A, P8.
But again, I think the whole consent thing is it is you tailor it to the patient... So it's very hard to say *"this is the way you should be imparting that information"*. A, P9.

[we] will communicate with the same person in a different way, depending on the situation. A,P7.
We are all different people…we're better off getting a broad experience of seeing how different people do these things in order to work out actually what would work for our particular communication style or personality to try and keep things as, as shared and as broad as possible. TO, P22.
Sometimes women get less a whole lot less from me, than perhaps they should because I'm tired and rushed. A, P9.
So I think every single woman you tailor what you say differently. It's all according to like you say what, or how, or your perception of their understanding as well. IMW, P12.
There are days when you're better at it, then there are days when you think, *"Oh, God, I could have done that better"* CMW, P11.
I just think in general, communication is something that we bang on about all the time and you do it, you know…everyone's saying *"you know communication's key"*, but actually, the communication isn't always there. IMW, P3
Skills around the actual conversation could be improved… you're making me think there's some teaching sessions we could be doing here. A, P9
I'd like a trainee to sit in clinic, and [name] trained in [place] as I did, and there was consultant there, who actually, came and sat in with you in clinic, and he sat there with you while you consulted, and by golly, your consultation style, improved, your feedback, etc… I think hands on direct, consultant, training like that, is really important. CO, 17.
Subtheme 1.4: Adapting to the individual needs and preferences of the woman
Whilst we want to give women this information, to try and empower them, and hopefully make things better, that I think there will be a group of patients who who will, they won't want that information, because they'll find it potentially very scary, or, you know that, but certainly, it might put some barriers up to accepting that information. TO, P22.
They don't realise that they can discuss that option. So I think when when, when you present them with an opportunity to discuss this, whatever problem they might have, um they're quite welcoming... I think sometimes they're quite surprised that that actually can happen, that they can discuss.... Whatever point they've they've come across with somebody. CMW, P2.
It's almost like continuing to give them permission that they can say what they feel, or they can say what they want or, you know, and then ...so there's that in the process of continuing to say, "*You have choice, this isn't prescription*" BCMW, P6
I think using an interpreter for people with a language barrier has a profound impact on trying to communicate in an emergency or even semi emergency situation. If I have someone on the labour ward who in any way might need a caesarean, sometimes in the middle of night, I find it quite useful to go in and go through a consent form with a translator in advance of doing a procedure because I think for those women communicating with them is so incredibly difficult... I think undoubtedly those women making intrapartum decision making is like the dreaded decision making because it's a difficult thing to communicate if both people have a shared first language, let alone with a language line and a phone interpreter and a possible partner. I think that's just like a perfect storm of issues.TO, P21.
You've got the whole range of the tertiary level educated patient who doesn't want us to do anything versus quite often someone who maybe left school after GCSEs...but you still have to provide both of those sets of patients with all the same information, but you have to then guide how you do that. And that's, that can be quite challenging. TO, P22.
