# Supplementary material for: Maternity healthcare professionals’ experiences of supporting women in decision-making for labour and birth: a qualitative study
Source: BMJ Open. 2024 Apr 28;14(4):e080961. doi: 10.1136/bmjopen-2023-080961 (PMC11057275; doi:10.1136/bmjopen-2023-080961)
Supplement: Supplementary data [file bmjopen-2023-080961supp005.pdf]

| **Supplementary Table 5. Theme 2: Controversies in current decision-making practices.** |
|---|
| Subtheme 2.1. Clinical expertise and personal experience in decision making |
| So what you would tend to do in that situation is probably stress the the downsides of having a general anaesthetic and talk about actually, you know, failed intubation ...So actually, we will manipulate that conversation based on us...thinking we actually probably do know the best thing for that patient. A, P8. |
| Yeah, exactly like a midwife, led unit midwife describing a breech in [place] where they do it quite frequently-ish, versus like a consultant who works in HCIB describing it at [place], that is a very different description that you will receive. TO, P21 |
| We do all subconsciously do that, we select which bits of information we think the patient needs. A, P9. |
| Can you distinguish bias from experience? Or from or from teaching? I suppose, in that we're coming from a clinical viewpoint where we're...How do I put this? ...I'm trying to sort of say that it's not necessarily a biased opinion. Whereas I suppose what I'm trying to get at is that as a hopefully an experienced clinician, is it still bias? TO, P22 |
| And so like if I don't want to induce a patient at 37 weeks for a pretty benign reason, but the patient is really keen to be induced, I will give them the figures for nicu admission, whereas if there's a patient who I want to induce, I might not necessarily tell them that same information. TO, P23. |
| Because we all know that, you know, with risks and percentages and risk ratios, etc, you can you can lean any decision to different ways. CO, P19. |
| I sort of feel like women are very coerced. ..And I feel like the information that's shared with women isn't neutral. They're scared into stuff. CMW, P12. |
| you acknowledge your bias and say, "*Well, obviously, I'm a consultant obstetrician, and I see, you know, a lot of high risk, and therefore I am biased*" CO, P18. |
| [the BRAIN app] is really good because it gives a really good balance and what are the risks, what are the benefits, what are the alternatives, what are the family's preferences. So it just it's a really good tool for facilitating those shared decisions, and looking at other people's perspectives as well. CMW, P2. |
| I do think sometimes putting numbers on things [by using absolute risk rather than relative risk] does help to give a kind of a more fair picture and allow people to make decisions that are maybe, well you know, just informs them and then they can make the decision they feel is right for them. TO, P24. |
| Subtheme 2.2. Conditions limiting validity of consent |
| I think in an emergency situation, I find it very difficult , because I think the consent process I currently go through seems like a bit of a sham… we go through this process of waving a consent form at them saying "*you and your baby going to die. If we don't do this*". TO, P21. |
| when you're trying to consent a labouring woman for an epidural, and she's screaming, "*just put it in*" at you that, you know, they don't take on board, we could tell them the risks were, you know, "*1 in 2 risk of death*" or something, at that point, they're not listening to you at all. A,P8. |
| We cannot say that a woman in labour is giving true consent, even for an epidural, when she has so much pain…She's so crippled and tired and, you know, fed up with everything, that she'll just agree to anything. IMW, P12. |
| I've never seen anyone try and do a decision making kind of conversation at the time of a shoulder dystocia, and I've also never come across a mum who has retrospectively said, "*I can't believe you didn't talk to me about that first*." TO, P24 |
| "*Why was it five minutes before they took me around to theatre that somebody suddenly mentioned that I might end up having a caesarean section, why? When there were nine months when I could have been counselled about this*." TO, P21 |

| |
|---|
| We've got sort of risks of general anaesthesia, regional anaesthesia, we've got these lovely information cards from the OAA (Obstetric Anaesthetists' Association). A, P22 |
| Subtheme 2.3. Challenges faced when women decline medical advice |
| She knew the risk, but she was absolutely clear what the risks were, what the implications could be what the outcome could be for her baby, but, that was the decision that she wanted. And it's it was so difficult. IMW, P3. |
| I think it is the fear of, of litigation, and that defensive practice, which is the overwhelming you know, feeling. I know, I've had some personal experiences around that. So that definitely does probably change the way I practice as a midwife, making me perhaps more overcautious… it's that kind of fear of, if something happens or goes wrong the responsibility then lies with you as the midwife, and the woman…will turn around and say, "*Well, that was something that you didn't do,*" Or "*if you'd have told me something differently, that wouldn't have happened*.". BCMW, P5. |
| They won't let you deliver that baby. And I find that always challenging and it takes maybe 12, 24 or 48 hours before you're allowed to do that. And then that baby obviously has, may have problems. And they're the ones I really struggle with…and it gets turned very much back against you as the medical professional saying, "*Why didn't you explain that this might happen*?" Even if it's been written in black and white. TO, P23 |
| Yeah, it's, it's massive that and um what support networks are in there? Because at the end of the day, you still got another, you know, 50 women on your caseload that you've got to look after. IMW, P1 |
| You need to talk about every option, not just the one you want them to do, and so you need to be really facetious about it.. It's a nightmare...CO, P18 |
| If you give women too much information, you're just scare mongering, you know, if I say "*you've got this percentage chance and this percentage or whatever*". So it is difficult. CO, P19 |
| The trace was pretty horrid... And she really did need to have a caesarean section, but she'd made the decision... And, and that's what we did. And I actually felt although it was, it wasn't a pleasant experience, it actually, for me, it was positive, because I know that we'd I'd worked with both with the couple as much as I possibly could. DSMW, P15 |
| It's like women who decline induction, it's like, well, we'll tell you about the risks again, because you aren't doing what we've decided is the right thing to do from our perspective of, you know, recommendations. *"Remember, it's on you now"*...You know, and therefore, it's not shared decision making. BCMW, P6. |
| That might be because we've alienated people as well. So I think with with regards to pre birthing, and birthing outside of guidance. CO, P19. |
| They were they were quite bullish, actually in the hospital, they kept ringing her but she just turned the phone off in the end and said," I'm not going to speak to you, I need a day off from all of you. CMW, P11. |
