# Supplementary material for: Maternity healthcare professionals’ experiences of supporting women in decision-making for labour and birth: a qualitative study
Source: BMJ Open. 2024 Apr 28;14(4):e080961. doi: 10.1136/bmjopen-2023-080961 (PMC11057275; doi:10.1136/bmjopen-2023-080961)
Supplement: Author's manuscript [file bmjopen-2023-080961.draft_revisions.pdf]

BMJ Open is committed to open peer review. As part of this commitment we make the peer review history of every article we publish publicly available.

When an article is published we post the peer reviewers' comments and the authors' responses online. We also post the versions of the paper that were used during peer review. These are the versions that the peer review comments apply to.

The versions of the paper that follow are the versions that were submitted during the peer review process. They are not the versions of record or the final published versions. They should not be cited or distributed as the published version of this manuscript.

BMJ Open is an open access journal and the full, final, typeset and author-corrected version of record of the manuscript is available on our site with no access controls, subscription charges or pay-per-view fees (http://bmjopen.bmj.com).

If you have any questions on BMJ Open's open peer review process please email info.bmjopen@bmj.com

BMJ Open

Maternity healthcare professionals perspectives of decision-making in the UK: a qualitative study

Journal:	*BMJ Open*
Manuscript ID	bmjopen-2023-080961
Article Type:	Original research
Date Submitted by the Author:	15-Oct-2023
Complete List of Authors:	Hardman, Kitty; University of Bristol, Bristol Medical School; North Bristol NHS Trust<br>Davies, Anna; University of Bristol, Centre for Academic Child Health<br>Demetri, Andrew; University of Bristol, Bristol Medical School; North Bristol NHS Trust, Department of Women’s and Children’s Health<br>Clayton, Gemma; University of Bristol, Faculty of Health Sciences<br>Bakhbakhi, Danya; University of Bristol, Centre for Academic Women's Health<br>Birchenall, Kate; University of Bristol, Bristol Medical School<br>Barnfield, Sonia; North Bristol NHS Trust, Department of Women's and Children's Health<br>Fraser, Abigail; University of Bristol, School of Social and Community Medicine<br>Burden, Christy; University of Bristol Faculty of Health Sciences<br>McGuinness, Sheelagh ; University of Bristol, Law; University of Bristol Law School<br>Miller, Rachel; Independent patient representative<br>Merriel, Abi; University of Bristol, Bristol Medical School; University of Liverpool, Institute of Life Course and Medical Sciences, Department of Women's and Children's Health, Centre for Women's Health Research
Keywords:	Decision Making, OBSTETRICS, QUALITATIVE RESEARCH, Clinical Decision-Making

SCHOLARONE™
Manuscripts

I, the Submitting Author has the right to grant and does grant on behalf of all authors of the Work (as defined in the below author licence), an exclusive licence and/or a non-exclusive licence for contributions from authors who are: i) UK Crown employees; ii) where BMJ has agreed a CC-BY licence shall apply, and/or iii) in accordance with the terms applicable for US Federal Government officers or employees acting as part of their official duties; on a worldwide, perpetual, irrevocable, royalty-free basis to BMJ Publishing Group Ltd ("BMJ") its licensees and where the relevant Journal is co-owned by BMJ to the co-owners of the Journal, to publish the Work in this journal and any other BMJ products and to exploit all rights, as set out in our licence.

The Submitting Author accepts and understands that any supply made under these terms is made by BMJ to the Submitting Author unless you are acting as an employee on behalf of your employer or a postgraduate student of an affiliated institution which is paying any applicable article publishing charge ("APC") for Open Access articles. Where the Submitting Author wishes to make the Work available on an Open Access basis (and intends to pay the relevant APC), the terms of reuse of such Open Access shall be governed by a Creative Commons licence – details of these licences and which Creative Commons licence will apply to this Work are set out in our licence referred to above.

Other than as permitted in any relevant BMJ Author's Self Archiving Policies, I confirm this Work has not been accepted for publication elsewhere, is not being considered for publication elsewhere and does not duplicate material already published. I confirm all authors consent to publication of this Work and authorise the granting of this licence.

For peer review only

Title page

Maternity healthcare professionals perspectives of decision-making in the UK: a qualitative study

Shortened running title:

Decision-making in maternity care: a UK qualitative study

Authors:

Kitty Hardman[1,2], Anna Davies[1,], Andrew Demetri[1,2], Gemma Clayton[1], Danya Bakhbakhi[1], Kate Birchenall,[1] Sonia Barnfield[2], Abigail Fraser[1], Christy Burden[1,2], Sheelagh McGuinness[3], Rachel Miller[4], Abi Merriel.[1,5]

1. [1]University of Bristol Medical School, University of Bristol, UK
2. [2]Department of Women's and Children's Health, North Bristol NHS Trust, UK
3. [3]University of Bristol Law School, University of Bristol, UK
4. [4]Independent patient representative
5. [5]Centre for Women's Health Research, Department of Women's and Children's Health, Institute of Life Course and Medical Sciences, University of Liverpool, UK

Corresponding author: Dr Abi Merriel, abi.merriel@liverpool.ac.uk, https://orcid.org/0000-0003-0352-2106

Address: Centre for Women's Health Research, Department of Women's and Children's Helath, Institute of Life Course and Medical Sciences, Faculty of Health & Life Sciences, University of Liverpool, Liverpool Women's Hospital, Crown Street, L8 7SS

For peer review only

For peer review only - http://bmjopen.bmj.com/site/about/guidelines.xhtml

Abstract

Objectives

To explore and characterise maternity healthcare professionals' (MHCPs) experience and practice of informed decision-making (IDM), to inform policy, research and practice development.

Design

Qualitative focus group study.

Setting

Online with MHCPs from a single maternity unit in the Southwest of England.

Participants

MHCPs who give information relating to clinical procedures and pregnancy care and are directly involved in decision-making conversations purposively sampled from a single National Health Service (NHS) Trust.

Data collection: A semi-structured topic guide was used.

Data Analysis: Reflexive thematic analysis.

Results

Twenty-four participants attended seven focus groups. Two themes were developed: *contextualising decision-making* and *controversies in current decision-making*. Contextual factors that influenced decision-making practices included lack of time, and challenges faced in intrapartum care. MHCPs reported variation in how they approach decision-making conversations and asked for more training on how to consistently achieve IDM. There were communication challenges with women/birthing people who do not speak English. Three controversies were explored in the controversies theme: the role of prior clinical experience, the

For peer review only

For peer review only - http://bmjopen.bmj.com/site/about/guidelines.xhtml

validity of informed consent when women/birthing people were in pain and during emergencies, and instances where women/birthing people declined medical advice.

Conclusions

We found that MHCPs are committed to IDM but need better support to deliver it consistently. Structured processes including core information sets, communication skills training and the decision support aids may help to standardise the information and better support IDM.

Strengths and limitations

- Multi-disciplinary perspective: community, integrated care, birth centre and delivery suite midwives, consultant and trainee obstetricians and specialist associate and consultant anaesthetists.
- Moderated focus group study design enabling generation of rich data
- Online setting allowing safe collection of data during COVID-19 pandemic
- Limited to single healthcare trust
- Limited to experiences of maternity healthcare professionals

Original study protocol Available via supplementary material, S1.

Word count 3744

For peer review only

For peer review only - http://bmjopen.bmj.com/site/about/guidelines.xhtml

Introduction

Informed decision-making (IDM) is fundamental to clinical practice(1, 2, 3, 4, 5, 6). It is a process where the woman/birthing person is at the centre of their care, and is able to share information regarding decision-making preferences, personal values and beliefs, and where the clinician provides information about benefits and risks of management options to enable an autonomous, informed decision(4, 5, 7).

IDM differs from shared decision-making (SDM) as it unequivocally acknowledges women/birthing people as the decision-makers (3, 4, 5, 8). Informed consent (IC), often the endpoint of IDM, is where the woman/birthing person makes an informed, voluntary choice of treatment, and is often symbolised by signing a consent form(3, 9). Maternity healthcare professionals (MHCPs) are legally bound to achieve IC prior to providing treatment(4, 9).

IDM is an international healthcare priority(3, 10, 11) . It provides short- and long-term benefits through improved birth experiences, satisfaction with care regardless of outcome, improved maternal mental health outcomes, reduced pre-term birth, higher birth weights, and enables safer care(12, 13, 14, 15, 16). Failing to involve women/birthing people can lead to their feeling out of control and powerless, leading to negative and traumatic birth experiences, increased rates of postnatal depression, anxiety and PTSD (15, 17).

Decision-making occurs throughout pregnancy. However, achieving intrapartum and emergency IDM poses unique challenges; there may be limited time to discuss options, and the woman/birthing person may be tired, in pain and feeling anxious. Frequently cited barriers to practising IDM are time pressures and lack of clinical applicability, i.e. a belief that IDM is inappropriate in that clinical situation (18).

Despite these challenges, the Royal Colleges of Emergency Medicine, Obstetrics and Gynaecology and Midwifery provide limited emergency specific decision-making guidance(5, 7, 19). The General Medical Council (GMC) advise taking a proportional approach to emergency decision-making, which leaves MHCPs to subjectively interpret best practice. Given limited guidance and challenges posed by emergency care, it is

For peer review only

unsurprising that unplanned/emergency obstetric interventions confer the greatest sense of perceived loss of control and choice, and are associated with the poorest psychosocial outcomes (20, 21).

Guidance is needed for IDM in maternity, and especially intrapartum care, to achieve better psychosocial outcomes for women/birthing people and to support MHCPs (13). We aimed to understand MCHP's experience of decision-making from a multi-disciplinary perspective in maternity care as a foundation to develop interventions to improve practice.

Methods

The standards for reporting qualitative research (SRQR), checklist guided reporting of this study, see supplementary table, S2 (22).

Patient and public involvement

A patient representative was a member of the project steering committee and contributed to protocol design.

Research team and reflexivity

The research team comprised obstetricians, a research psychologist, patient representative, a lawyer, information specialist and epidemiologists. Data collection and analysis was carried out by KH, AKD, AD and AM. KH and AD are trainee obstetricians and early career academics, AKD, a research psychologist, with qualitative and maternity research experience, AM is an academic obstetrician. We considered possible over-representation of the MHCP perspective, therefore a non- MHCP facilitator attended focus groups and multidisciplinary discussion of candidate themes was undertaken by the research team.

Study design

For peer review only

For peer review only - http://bmjopen.bmj.com/site/about/guidelines.xhtml

Moderated focus groups explored MHCPs' knowledge and practice of IDM, mainly in relation to decision-making for labour and birth. Focus groups provide an open, supportive environment that facilitate in-depth discussions about sensitive and personal topics, leading to new and unexpected knowledge(23).

Participant selection, sampling and sample size

Participants were purposively sampled from a single trust in the south-west of England, with 6,000 deliveries annually. We aimed to sample across the range MHCPs who give information relating to clinical procedures and pregnancy care. We targeted: midwives (community, integrated care, birth centre and delivery suite) and doctors (consultant obstetricians and anaesthetists and their trainees). Focus groups were between three and seven participants to ensure each person had the opportunity to contribute. Potential participants were approached via email, posters, and word of mouth. Participant information leaflets were emailed to interested participants and remote informed consent, demographic data, and anonymised record ID numbers were generated and recorded using REDCap (24, 25). Participants received a £10 e-voucher. Recruitment continued until no new themes emerged (26).

Data collection

Moderated focus groups were held online in July 2021.The primary moderator (KH) asked questions, whilst the second moderator (AKD/AD) took field notes. A topic guide structured the discussions but once they begun, the natural flow was not interrupted. An encrypted audio recording device was used, audio-recordings were transcribed verbatim, and uploaded into NVivo (27).

Data analysis

An experiential orientation to data interpretation was adopted, meaning was derived through personal experiences, and how individuals process these experiences (28). Reflexive thematic analysis was undertaken using an iterative process (see supplementary table, S3) (29, 30). An inductive (bottom-up) approach was taken to develop the themes, whereby codes and themes were directly linked to the data,

For peer review only - http://bmjopen.bmj.com/site/about/guidelines.xhtml

For peer review only

however a degree of deductive (top-down) analysis was used to ensure the research question remained at the forefront (30, 31). Each phase was carried out independently and then discussed collaboratively (KH, AKD and AM).

Results

Twenty-Four participants attended seven focus groups in July 2021. Participants included community (CMW), integrated care and diabetic midwives (IMW), birth centre (BCMW) and delivery suite (DSMW) midwives, trainee (TO) and consultant obstetricians (CO), and specialist associate and consultant anaesthetists (A). See supplementary table, S4 for participant demographics.

Two overarching themes were developed, theme 1 C*ontextualising decision-making*, and theme 2 *Controversies in current decision-making practices.* Figure 1 illustrates each theme and component subthemes. Select quotations supporting each theme are presented in Tables 1 and 2, with full list of quotations in supplementary tables S5, S6. Each theme and subtheme will be discussed in turn.

Figure 1, Themes and subthemes

Theme 1: Contextualizing decision-making

Participants identified systemic barriers to IDM. They felt there was not enough time to adequately discuss management options, and described limiting the discussion to the time available. Participants felt more time could enable better discussions. MHCPs reported significant variation in the individual's approach to IDM. Women/birthing people were perceived to vary in their ability to participate in decision-making, particularly those who did not speak English.

Subtheme 1.1: "I keep coming back to time"

For peer review only - http://bmjopen.bmj.com/site/about/guidelines.xhtml

All groups felt their ability to achieve IDM was related to the time available, “I keep coming back to time… when you've got that time to actually provide some information” (TO, P22).

Having time enabled MHCPs to perform high-quality decision-making, which involved exploring preferences, addressing fears and building trusting relationships. However, the time routinely available was felt to be insufficient, “you are very much trying to limit the consultation based on the time that you're given for that woman. So I would say that when I'm allowed longer time with a woman I would think it was a more informed decision that was going to come out of that because I have time to listen” (CO, P17).

Experienced community midwives felt that systemic changes including reduced appointment times contributed to poorer IDM, “we used to spend hours sitting with every single woman before she delivered doing the birth plan, but it wasn't really the birth plan, it was a birth discussion…so she could tell you all her fears, and that would help with her decision-making process” (IMW, P1).

Participants described good decision-making experiences to involve multiple or longer appointments to build rapport, so women/birthing people could process information and deliberate decisions, however this was not the norm. For example, when a CMW described using multiple longer antenatal appointments another participant replied, “ So great [name] that you've managed to find space for someone, in a quite a complex situation, but for the *majority* of women, it's a very superficial process… So we really need to improve that for, you know, for every woman”(IMW, P3).

Subtheme 1.2: Intrapartum decision-making

IDM during labour was challenging, women/birthing people were felt to have limited cognitive capacity to engage in conversations due to pain, fatigue and emotional exhaustion, and decisions were time dependent, “you've not got time to just pause the body, give everyone a break, give the woman 25 minutes to process the information” (BSMW, P6). Presenting women/birthing people with new information and multiple

For peer review only

For peer review only - http://bmjopen.bmj.com/site/about/guidelines.xhtml

options during labour can be "derailing" and "traumatising". IDM approaches adopted in the antenatal setting were felt to be inappropriate.

Groups emphasized the need for improved antenatal education so that women/birthing people arrived at the point of birth informed of the main options, and knowledgeable of their preferences, so that discussions in labour did not require giving new information, or unexpected choices.

Subtheme 1.3: Variation in practice

Decision-making practice varied within and between MHCPs. Communication skills and how information was imparted to women/birthing people varied with time, the decision, the patient, and the clinician, "every single situation, every woman is different, every doctor is different, every interaction is different. All you can do is keep honing your skills, practising and doing your best. [There] definitely isn't one way of doing it..." (A,P8)

All groups described an ad-hoc approach to developing communication skills for decision-making , with none receiving formal training. The obstetrician and anaesthetist groups discussed the importance of learning from senior clinicians, "seeing how different people do these things in order to work out actually what would work for our particular communication style or personality to try and keep things as, as shared and as broad as possible" (TO, 22). The midwifery groups reported fewer on-the-job learning opportunities. Participants felt communication skills training would improve consultations.

All participants reported variation in their ability to achieve IDM, "there are days when you're better at it, then there are days when you think, "*Oh, God, I could have done that better*"(CMW, P11). Factors such as fatigue, hunger and stress were felt to also contribute to how well they carried out IDM, "sometimes women get less a whole lot less from me, than perhaps they should because I'm tired and rushed" (A, P9).

Subtheme 1.4: Accessibility of decision-making conversations

For peer review only - http://bmjopen.bmj.com/site/about/guidelines.xhtml

There was perceived variation in women/birthing people's ability to participate in decision-making conversations, for example if they do not speak English. A trainee obstetrician reported, "using an interpreter for people with a language barrier has a profound impact on trying to communicate in an emergency or even semi-emergency situation"(TO, P21). MHCPs reported using telephone interpreter services and partners to achieve IDM during intrapartum care, and described it as a "perfect storm of issues"(TO, P21). One participant described pre-emptive conversations about emergency scenarios. Others highlighted the challenges of ensuring patients from all socio-demographic backgrounds were equally informed, "you've got the whole range of the tertiary level educated patient who doesn't want us to do anything versus quite often someone who maybe left school after GCSEs...but you still have to provide both of those sets of patients with all the same information…that can be quite challenging" (TO, P22).

For peer review only

For peer review only - http://bmjopen.bmj.com/site/about/guidelines.xhtml

Table 1, Theme 1 Contextualizing decision-making.

**Theme 1: Contextualising decision-making**
Subtheme 1.1. "I keep coming back to time."
I keep coming back to time, maybe this is ....what I keep coming back to, but you know, it's time to process. Process that information, and then come to as [name] said, you know, what might not necessarily be what we think is the right decision, from our perspective, but when it comes to the patient, and you're bringing all that information together, they feel that's the right decision for them. TO, P22
The midwives that we all work with are incredibly stressed, underfunded, under great time pressures, and there are not enough of them to do the work that is required and the population is increasing and their workload is increasing. TO, P23.
And I feel like shared decision making is something that we all aspire to in situations where we as clinicians feel that there is enough time.TO, P24.
Subtheme 1.2: Intrapartum decision-making
There are times that it isn't always possible to give them ...accommodate them having a discussion about something sometimes you do have to make…more channelled decision making. BCMW, P5.
You have to we, we have to, and also the birth educators that we currently have in this country have to start having conversation from the very first antenatal class that they hold. BCMW, P6.
At the point where they're in… the process of the labour…that too much choice at that point is actually really derailing. And then I felt like I've left conversations thinking, why did I even? Why did I even do that to that poor woman? ..Like she's now on the edge to a really traumatic experience, because I've given her those choices and tried to say, look, there are other ways you can do X, Y, and Z. BCMW, P6.
Subtheme 1.3: Variation in practice
I think there's just such a massive variety of sources of information that women receive and I don't think there's a huge amount of standardisation. TO, P22
So I think every single woman you tailor what you say differently. It's all according to like you say what, or how, or your perception of their understanding as well. IMW, P12.
There are days when you're better at it, then there are days when you think, "*Oh, God, I could have done that better*." CMW, P11.
Communication is something that we bang on about all the time and you do it, you know…everyone's saying "*you know communication's key*", but actually, the communication isn't always there. IMW, P3
Subtheme 1.4: Accessibility of decision-making conversations
whilst we want to give women this information, to try and empower them, and hopefully make things better, that I think there will be a group of patients who who will, they won't want that information, because they'll find it potentially very scary, or, you know that, but certainly, it might put some barriers up to accepting that information. TO, P22.
I think using an interpreter for people with a language barrier has a profound impact on trying to communicate in an emergency or even semi emergency situation. If I have someone on the labour ward who in any way might need a caesarean, sometimes in the middle of night, I find it quite useful to go in and go through a consent form with a translator in advance of doing a procedure because I think for those women communicating with them is so incredibly difficult.TO, P21.
You've got the whole range of the tertiary level educated patient who doesn't want us to do anything versus quite often someone who maybe left school after GCSEs...but you still have to provide both of those sets of patients with all the same information, but you have to then guide how you do that. And that's, that can be quite challenging. TO, P22.

For peer review only - http://bmjopen.bmj.com/site/about/guidelines.xhtml

Theme 2: Controversies in current decision-making practices.

Theme 2 explores controversies in current decision-making practices: participants reported providing information in a way that aligned with their clinical perspective; pain and emergency situations were felt to limit the validity of IC; women/birthing people declining medical advice was challenging and MHCPs were fearful of medicolegal repercussions, whilst these women/birthing people were made to feel isolated.

Subtheme 2.1: Clinical expertise and personal experience in decision-making

All groups reported bringing their clinical expertise, training and experience to decision-making conversations, resulting in women/birthing people receiving differing information from different MHCPs, "a [midwife] describing a breech where they do it quite frequently-ish, versus like a consultant who works in HSIB [Healthcare Safety Investigation Branch] that is a very different description that you will receive." (TO, P21)

Two distinct issues became apparent. First, the way in which MHCPs conducted decision-making conversations and the information provided to women/birthing people was influenced by training, experience and individual interpretation of the available evidence, and was described by some as their personal or clinical bias. This was felt to be very difficult to mitigate.

Second, there were occasions where participants felt that they presented information differently depending on the particular clinical situation, "so like if I don't want to induce a patient at 37 weeks for a pretty benign reason, but the patient is really keen to be induced, I will give them the figures for NICU admission, whereas if there's a patient who I want to induce, I might not necessarily tell them that same information"(TO, P23). This practice was reported most amongst the obstetrician and anaesthetist groups.

Some participants had insight about their potential clinical biases. They discussed the importance of using absolute rather than relative risk and infographics to help communicate information objectively. Two participants described using a decision tool to help standardise information.

For peer review only - http://bmjopen.bmj.com/site/about/guidelines.xhtml

For peer review only

Subtheme 2.2: Conditions limiting the validity of consent

MHCPs believed that severe pain and life-threatening emergencies meant it was near-impossible to achieve IC, let alone IDM.

The anaesthetic and midwifery groups felt that many women/birthing people were unable to weigh up risks and benefits of an epidural when they were in so much pain, “when you're trying to consent a labouring woman for an epidural, and she's screaming, “just put it in” …we could tell them the risks were, you know, "1 in 2 risk of death" or something, at that point, they're not listening to you at all” (A,P8). The anaesthetic group felt pre-emptive conversations regarding epidural analgesia were important, and reported using information cards to support this.

All groups questioned whether IDM and IC is possible in life-threatening situations. A trainee obstetrician reported, “I’ve never seen anyone try and do a decision-making kind of conversation at the time of a shoulder dystocia, and I've also never come across a mum who has retrospectively said, *“I can't believe you didn't talk to me about that first”(*TO, P24). However they reported following process and signing consent forms, despite feeling it doesn’t reflect IDM or IC. A trainee obstetrician reported, “I think in an emergency situation, I find it very difficult , because I think the consent process I currently go through seems like a bit of a sham… we go through this process of waving a consent form at them saying, “you and your baby going to die if we don't do this ”(TO, P21).

Subtheme 2.3: Challenges faced when women/birthing people decline medical advice

Decision-making conversations are challenging when women/birthing people decline medical advice. MHCPs were psychologically affected by poor neonatal and maternal outcomes and fearful of medicolegal repercussions, “it gets turned very much back against you as the medical professional saying, *Why didn't you explain that this might happen?* Even if it's been written in black and white…” (TO,P23)

For peer review only - http://bmjopen.bmj.com/site/about/guidelines.xhtml

Several participants reported practising more defensively having experienced poor neonatal outcomes. Furthermore, participants from all groups felt they needed to explain all risks to women/birthing people to protect themselves against litigation, "it's that kind of fear of, if something happens or goes wrong the responsibility then lies with you as the midwife, and the woman…will turn around and say, "*Well, that was something that you didn't do,*" Or "*if you'd have told me something differently, that wouldn't have happened*."" BCMW, P5

Yet this approach infringed on women/birthing people's experience of decision-making, women/birthing people were reportedly harassed when they declined medical advice, and made to feel that their decisions were not respected by repeatedly being told the risks of declining medical advice, or being repeatedly offered medical interventions. A community midwife described a woman having to turn her phone off due to avoid repeated phone calls offering an induction of labour.

For peer review only - http://bmjopen.bmj.com/site/about/guidelines.xhtml

Table 2, Theme 2: Controversies in current decision-making practices.

**Table 2, Theme 2: Controversies in current decision-making practices.**
Subtheme 2.1. Clinical expertise and personal experience in decision making
So what you would tend to do in that situation is probably stress the the downsides of having a general anaesthetic and talk about actually, you know, failed intubation ...So actually, we will manipulate that conversation based on us...thinking we actually probably do know the best thing for that patient. A, P8.
We do all subconsciously do that, we select which bits of information we think the patient needs. A, P9.
I sort of feel like women are very coerced. ..And I feel like the information that's shared with women isn't neutral. They're scared into stuff. CMW, P12.
[The BRAIN app] is really good because it gives a really good balance and what are the risks, what are the benefits, what are the alternatives, what are the family's preferences. So it just it's a really good tool for facilitating those shared decisions, and looking at other people's perspectives as well. CMW, P2.
I do think sometimes putting numbers on things [by using absolute risk rather than relative risk] does help to give a kind of a more fair picture and allow people to make decisions that are maybe, well you know, just informs them and then they can make the decision they feel is right for them. TO, P24.
Subtheme 2.2. Conditions limiting validity of consent
I think in an emergency situation, I find it very difficult , because I think the consent process I currently go through seems like a bit of a sham... we go through this process of waving a consent form at them saying "*you and your baby going to die. If we don't do this*". TO, P21.
When you're trying to consent a labouring woman for an epidural, and she's screaming, "*just put it in*" at you that, you know, they don't take on board, we could tell them the risks were, you know, "*1 in 2 risk of death*" or something, at that point, they're not listening to you at all. A,P8.
We cannot say that a woman in labour is giving true consent, even for an epidural, when she has so much pain...She's so crippled and tired and, you know, fed up with everything, that she'll just agree to anything. IMW, P12.
I've never seen anyone try and do a decision making kind of conversation at the time of a shoulder dystocia, and I've also never come across a mum who has retrospectively said, "*I can't believe you didn't talk to me about that first*." TO, P24
Subtheme 2.3. Challenges faced when women/birthing people decline medical advice
She knew the risk, but she was absolutely clear what the risks were, what the implications could be what the outcome could be for her baby, but, that was the decision that she wanted. And it's it was so difficult. IMW, P3.
I think it is the fear of, of litigation, and that defensive practice, which is the overwhelming you know, feeling. I know, I've had some personal experiences around that. So that definitely does probably change the way I practice as a midwife, making me perhaps more overcautious… it's that kind of fear of, if something happens or goes wrong the responsibility then lies with you as the midwife, and the woman...will turn around and say, "*Well, that was something that you didn't do,*" Or "*if you'd have told me something differently, that wouldn't have happened*.". BCMW, P5.
They won't let you deliver that baby. And I find that always challenging and it takes maybe 12, 24 or 48 hours before you're allowed to do that. And then that baby obviously has, may have problems. And they're the ones I really struggle

For peer review only - http://bmjopen.bmj.com/site/about/guidelines.xhtml

with…and It gets turned very much back against you as the medical professional saying, "*Why didn't you explain that this might happen*?" Even if it's been written in black and white. TO, P23
Yeah, it's, it's massive that and um what support networks are in there? Because at the end of the day, you still got another, you know, 50 women on your caseload that you've got to look after. IMW, P1
If you give women too much information, you're just scare mongering, you know, if I say "*you've got this percentage chance and this percentage or whatever*". So it is difficult. CO, P19
It's like women who decline induction, it's like, well, we'll tell you about the risks again, because you aren't doing what we've decided is the right thing to do from our perspective of, you know, recommendations. *"Remember, it's on you now*"...You know, and therefore, it's not shared decision making. BCMW, P6.
That might be because we've alienated people as well. So I think with with regards to pre birthing, and birthing outside of guidance. CO, P19.
They were they were quite bullish, actually in the hospital, they kept ringing her but she just turned the phone off in the end and said," I'm not going to speak to you, I need a day off from all of you. CMW, P11.

For peer review only

For peer review only - http://bmjopen.bmj.com/site/about/guidelines.xhtml

Discussion

In this qualitative exploration of MHCPs experiences of decision-making, participants were motivated to involve women/birthing people in decision-making, and their definition of good decision making aligned with guidance on IDM(4, 5). However, challenges to IDM included: time pressures, lack of training and intrapartum/emergency care. MHCPS perceived that women/birthing people's desire and IDM accessibility varied, and non-English speaking women/birthing people faced communication challenges. Suggested changes to improve IDM were: increased consultation time, skills training, and improved antenatal care/education to better prepare women/birthing people for labour. Three areas of controversy were explored: the role of prior clinical experience in IDM, the validity of IC during intrapartum/emergency care, and when women/birthing people declined medical advice.

Interpretation in context

The need to deliver patient-centred care, with time to ask questions, express concerns and receive high-quality information coincides with increasing demands on healthcare systems(3, 4, 5, 32). A systematic review of decision-making found that time constraints are the most commonly cited barrier across cultural and organisational contexts (18). For IDM to be successfully implemented a systems approach needs to be considered to provide clinicians with time and resources to counsel women/birthing people(11) .

Decision aids can support MHCPs to standardise content, support risk communication, facilitate discussion about what matters patients, and reduce decisional conflict without extending consultations(33, 34, 35). In UK maternity care, use of decision aids is growing with tools to provide decision-making structure,(36) support discussion about mode of birth, (37) and intrapartum decision making (38).

However, it is unlikely that there can be a decision aid for every decision, and they are not universally acceptable or useful (39). One effective way of improving decision-making skills for clinicians is to role play using a decision aid for different decision options (39, 40). The NHS personalised care plan expects clinicians

For peer review only - http://bmjopen.bmj.com/site/about/guidelines.xhtml

to be trained in decision-making conversations (11). None of our participants had formal IDM training, MHCPs need to be equipped with the tools to support IDM, and opportunity to attend training.

Language poses a significant barrier to IDM. Women/birthing people who do not speak the local language face issues around communication and this may affect quality of care (41, 42). The National Institute for Health and Care Excellence (NICE) emphasises the importance of using clear language with resources translated into other languages if needed(42, 43). Our participants had developed strategies to manage decision-making in this group; it is important that the maternity system develops a strategy to support these vulnerable women/birthing people.

Participants' prior experiences influence their communication, and in some instances the decision chosen by the patient. These findings are in keeping with research from a range of specialities (20, 44). MHCPs have a duty to declare personal beliefs and potential biases to ensure transparency however, how often this happens in reality is unclear (4, 45). The use of decision aids may help to standardise information, and free it from clinicians' personal biases (33).

Participants expressed conflict between fear of litigation and patient autonomy when women/birthing people declined medical advice. Research suggests that MHCPs believe they incur ethical or legal liability if patients decline care, and may therefore try to persuade women/birthing people to accept intervention(46, 47). Structured, informed refusal processes may help MHCPs feel more confident in caring for these patients, and prevent women/birthing people from feeling ostracised (46, 48). MHCPs should be trained to explore the values underlying a woman's refusal, whilst emphasising patient choice. They should be enabled to maintain communication to facilitate safest possible care (20).

Participants questioned the validity of consent when women/birthing people were in pain, and during emergencies. Women/birthing people consented in an emergency are more likely to feel that they would have signed whatever was on the consent form, find the consent form harder to understand and are less likely to remember signing it, and their overall satisfaction with the consent process is lower (49). Focusing

For peer review only - http://bmjopen.bmj.com/site/about/guidelines.xhtml

on obtaining written consent in emergency scenarios may not achieve either informed choice or woman-centred care(7). Better birth preparation may improve this.

Participants suggested that presenting new information in labour can be overwhelming. Improving antenatal education and preparation for birth is vital to improving birth experiences (15, 46, 50). Consistent information throughout pregnancy is needed to enable IDM (36, 45). The development of core information sets regarding vaginal birth, unplanned assisted birth and unplanned emergency caesarean births offer one way which may help women/birthing people to receive consistent, accurate information, that is valued by them, whilst the use of decision aids may help to standardise and guide decision-making conversations (33, 51).

Strengths and limitations

Further research could involve participant recruitment from additional healthcare trusts and geographically and socially diverse areas. However, our findings are congruent with decision-making experiences across maternity settings suggesting these results may be relevant more broadly.

The online focus groups enabled the study to proceed during the COVID-19 pandemic, they created a relaxed atmosphere and enabled open discussion(23). However technical issues caused additional challenges. Furthermore, gaining perspectives of women/birthing people's experience of IDM is essential and work undertaken to address this is currently being analysed.

Conclusion

To improve women/birthing people's birth experiences, and to better support MHCPs a systems-wide approach to IDM must be considered. Better preparedness for birth with access to consistent information throughout pregnancy is important in ensuring women/birthing people are not faced with new or unexpected intrapartum choices. The development of core information sets, better support tools, and training for staff will help women/birthing people to receive standardised information relevant to them.

For peer review only

For peer review only - http://bmjopen.bmj.com/site/about/guidelines.xhtml

MHCPs must be supported in providing advice and care to women/birthing people birthing outside of guidelines with well-defined pathways for those who decline medical advice. Decision-making and consent during intrapartum and emergency situations should be revisited given the concerns regarding its validity. MHCPs believe in IDM. It is important that research, training and their implementation matures alongside the health system to deliver IDM to all women/birthing people.

Acknowledgements

We thank all participants who contributed to the study.

Disclosure of Interests

None

Contribution to Authorship

KH contributed to the design of the study, data acquisition, data interpretation, drafting of the article and with the final approval to be published.

AKD contributed to the conception and design of study, acquisition of data, analysis, interpretation of data, drafting the article and with the final approval to be published.

AD, contributed to the acquisition of data, and final approval of the article to be published.

AM conceived the study and design, contributed to the acquisition of data, analysis, interpretation of data, drafting the article and final approval to be published.

GC, DB, KB, KB, SB, AF, CB, SM, RM contributed to study conception and design, and critical review of the final draft.

For peer review only

For peer review only - http://bmjopen.bmj.com/site/about/guidelines.xhtml

Details of Ethics Approval

20/SW/0035

South West – Frenchay Research Ethics Committee

Funding

This work was supported by a David Telling Trust award and the Elizabeth Blackwell Institute, University of Bristol, and funded in whole, or in part, by the Wellcome Trust [Grant number - 204813/Z/16/Z]. For the purpose of Open Access, the author has applied a CC BY public copyright licence to any Author Accepted Manuscript version arising from this submission.

References

1. NHS England. National maternity review. Better Births: Improving outcomes of maternity services in England - A Five Year Forward View for maternity care. London: NHS England; 2016.
2. NHS. Personalised care and support planning guidance. Guidance for local maternity systems.; 2021.
3. Informed consent and shared decision making in obstetrics and gynaecology. ACOG committee opinion. Number 819 (*Replaces Committee Opinion Number 363, April 2007, and Committee Opinion Number 439, August 2009*) [press release]. ACOG2021.
4. GMC. Guidance on professional standards and ethics for doctors. Decision making and consent: General Medical Council; 2020. Available from: https://www.gmc-uk.org/ethical-guidance/ethical-guidance-for-doctors/decision-making-and-consent.
5. Royal College of Midwives. Informed decision making.: Royal College of Midwives; 2022. Available from: https://www.rcm.org.uk/media/6007/informed-decision-making_0604.pdf.
6. Campbell M. Montgomery v Lanarkshire Health Board. Common Law World Review. 2015;44(3):222–8.
7. Royal College of Obstetricians and Gynaecologists. Obtaining Valid Consent. Clinical Governance Advice No.6. London: Royal College of Obstetricians and Gynaecologists; 2015.
8. Birthrights. Birthrights and GMC - what does informed consent mean in maternity care? : Birthrights; 2021 [Available from: https://www.birthrights.org.uk/2021/09/17/birthrights-and-gmc-what-does-informed-consent-mean-in-maternity-care/.
9. Department of Health and Social Care. Reference guide to consent for examination or treatment. London: Department of Health; 2009. Available from: www.dh.gov.uk/publications
10. World Health Organization (WHO). WHO global strategy on integrated people-centred health services 2016-2026. Switzerland: World Health Organization 2015. Available from: https://www.who.int/health-topics/integrated-people-centered-care#tab=tab_1.
11. NHS. Universal Personalised Care. Implementing the Comprehensive Model. 2019.
12. Care Quality Commission. Getting safer faster: key areas for improvement in maternity services. Available at: Safety, equity and engagement in maternity services: Care Quality Commission

For peer review only - http://bmjopen.bmj.com/site/about/guidelines.xhtml

(www.cqc.org.uk); 2021. Available from: https://www.cqc.org.uk/publications/themed-work/getting-safer-faster-key-areas-improvement-maternity-services.

13. Independent Maternity Review. Ockenden report – Final: Findings, conclusions, and essential actions from the independent review of maternity services at the Shrewsbury and Telford Hospital NHS Trust. Department of Health and Social Care; 2022 June, 6th, 2022.

14. Garcia ER, Yim IS. A systematic review of concepts related to women's empowerment in the perinatal period and their associations with perinatal depressive symptoms and premature birth. BMC Pregnancy and Childbirth. 2017;17(2):347.

15. Elmir R, Schmied V, Wilkes L, Jackson D. Women's perceptions and experiences of a traumatic birth: a meta-ethnography. Journal of Advanced Nursing. 2010;66(10):2142-53.

16. Nieuwenhuijze MJ, Korstjens I, de Jonge A, de Vries R, Lagro-Janssen A. On speaking terms: a Delphi study on shared decision-making in maternity care. BMC Pregnancy and Childbirth. 2014;14(223).

17. Beck CT. Birth trauma: in the eye of the beholder. Nurs Res. 2004;53(1):28-35.

18. Légaré F, Ratté S, Gravel K, Graham ID. Barriers and facilitators to implementing shared decision-making in clinical practice: Update of a systematic review of health professionals' perceptions. Patient Education and Counseling. 2008;73(3):526-35.

19. The Royal College of Emergency Medicine. Consent in Adults, Adolescents and Children in Emergency Departments: The Royal College of Emergency Medicine; 2018.

20. Birthrights. Dignity in Childbirth: The Dignity Survey 2013: Women's and midwives' experiences of dignity in UK maternity care.; 2013.

21. Yuill C, McCourt C, Cheyne H, Leister N. Women's experiences of decision-making and informed choice about pregnancy and birth care: a systematic review and meta-synthesis of qualitative research. BMC Pregnancy and Childbirth. 2020;20(1):343.

22. O'Brien BC, Harris IB, Beckman TJ, Reed DA, Cook DA. Standards for Reporting Qualitative Research: A Synthesis of Recommendations. Academic Medicine. 2014;89(9).

23. Wilkinson S. Focus groups in health research : exploring the meanings of health and illness. Journal of health psychology. 1998;3.

24. Harris PA, Taylor R, Minor BL, Elliott V, Fernandez M, O'Neal L, et al. The REDCap consortium: Building an international community of software platform partners. Journal of Biomedical Informatics. 2019;95:103208.

25. Harris PA, Taylor R, Thielke R, Payne J, Gonzalez N, Conde JG. Research electronic data capture (REDCap)—A metadata-driven methodology and workflow process for providing translational research informatics support. Journal of Biomedical Informatics. 2009;42(2):377-81.

26. Braun V, Clarke V. To saturate or not to saturate? Questioning data saturation as a useful concept for thematic analysis and sample-size rationales. Qualitative Research in Sport, Exercise and Health. 2021;13(2):201-16.

27. QSR International Pty Ltd. Nvivo (Released in March 2020): https://www.qsrinternational.com/nvivo-qualitative-data-analysis-software/home; 2020. Available from: https://www.qsrinternational.com/nvivo-qualitative-data-analysis-software/home.

28. Braun V, Clarke V. Using thematic analysis in psychology. Qualitative Research in Psychology. 2006;3(2):77-101.

29. Byrne D. A worked example of Braun and Clarke's approach to reflexive thematic analysis. Quality & Quantity. 2021;56:1391-412.

30. Braun V, Clarke V. Reflecting on reflexive thematic analysis. Qualitative Research in Sport, Exercise and Health. 2019;11(4):589-97.

31. Clarke V, Braun V. Successful Qualitative Research: A Practical Guide for Beginners. 1 ed. London: SAGE Publications Ltd; 2013.

32. All Party Parliamentary Groups. Safe staffing: the impact of staffing shortages in maternity and neonatal care. Report of the baby loss and maternity all party parliamentary groups.; 2022.

For peer review only

For peer review only - http://bmjopen.bmj.com/site/about/guidelines.xhtml

33. Poprzeczny AJ, Stocking K, Showell M, Duffy JMN. Patient Decision Aids to Facilitate Shared Decision Making in Obstetrics and Gynecology: A Systematic Review and Meta-analysis. Obstetrics and gynecology. 2020;135(2):444-51.
34. Stacey D, Légaré F, Lewis K, Barry MJ, Bennett CL, Eden KB, et al. Decision aids for people facing health treatment or screening decisions.: Cochrane Database for Systematic Reviews; 2017.
35. Légaré F, Ratté S, Stacey D, Kryworuchko J, Gravel K, Graham ID, et al. Interventions for improving the adoption of shared decision making by healthcare professionals. Cochrane Database Syst Rev. 2010(5):Cd006732.
36. NHS. Shared Decision Making Summary Guide: NHS England and NHS Improvement; 2019. Available from: https://www.england.nhs.uk/shared-decision-making/.
37. RCOG publishes resources to support pregnant women considering how to give birth [press release]. Royal College of Obstetricians and Gynaecologists2022.
38. Birthrights. IDECIDE - a new consent tool is on its way... 2020 [Available from: https://www.birthrights.org.uk/2020/01/30/idecide-a-new-consent-tool-is-on-its-way/.
39. Joseph-Williams N, Lloyd A, Edwards A, Stobbart L, Tomson D, Macphail S, et al. Implementing shared decision making in the NHS: lessons from the MAGIC programme. BMJ. 2017;357:j1744.
40. Elwyn G, Frosch D, Thomson R, Joseph-Williams N, Lloyd A, Kinnersley P, et al. Shared decision making: a model for clinical practice. Journal of general internal medicine. 2012;27(10):1361-7.
41. Chauvin P, Simonnot N, Douay C, Vanbiervliet F. Access to healthcare for the most vulnerable in a Europe in social crisis. 2014.
42. Knight M BK, Patel R, Shakespeare J, Kotnis R, Kenyon S, Kurinczuk JJ (Eds.) on behalf of MBRRACE-UK. Saving Lives, Improving Mothers' Care Core Report - Lessons learned to inform maternity care from the UK and Ireland Confidential Enquiries into Maternal Deaths and Morbidity 2018-20. Oxford: National Perinatal Epidemiology Unit, University of Oxford; 2022.
43. National Institute for Health and Care Excellence (NICE). Antenatal care. NICE guideline [NG201]. Evidence.: NICE; 2021. Available from: https://www.nice.org.uk/Guidance/NG201/evidence.
44. Lelie A. Decision-making in nephrology: shared decision making? Patient Educ Couns. 2000;39(1):81-9.
45. Begley K, Daly D, Panda S, Begley C. Shared decision-making in maternity care: Acknowledging and overcoming epistemic defeaters. J Eval Clin Pract. 2019;25(6):1113-20.
46. Kotaska A. Informed consent and refusal in obstetrics: A practical ethical guide. Birth. 2017;44(3):195-9.
47. Thompson A. Midwives' experiences of caring for women whose requests are not within clinical policies and guidelines. British Journal of Midwifery. 2013;21(8):564-70.
48. Jenkinson B, Kruske S, Stapleton H, Beckmann M, Reynolds M, Kildea S. Women's, midwives' and obstetricians' experiences of a structured process to document refusal of recommended maternity care. Women and Birth. 2016;29(6):531-41.
49. Akkad A, Jackson C, Kenyon S, Dixon-Woods M, Taub N, Habiba M. Informed consent for elective and emergency surgery: questionnaire study. BJOG: An International Journal of Obstetrics & Gynaecology. 2004;111(10):1133-8.
50. López-Toribio M, Bravo P, Llupià A. Exploring women's experiences of participation in shared decision-making during childbirth: a qualitative study at a reference hospital in Spain. BMC Pregnancy and Childbirth. 2021;21(1):631.
51. Demetri A, Davies A, Bakhbakhi D, Ijaz S. Vaginal birth core information set: study protocol for a Delphi study to achieve a consensus on a "core information set" for vaginal birth. BMJ Open. 2023.

For peer review only - http://bmjopen.bmj.com/site/about/guidelines.xhtml

NHS
Health Research Authority

NHS
North Bristol
NHS Trust

Study Protocol

Shared decision making for labour and birth

This protocol has regard for the HRA guidance and order of content

FULL/LONG TITLE OF THE STUDY

Shared decision making for labour and birth

For peer review only - http://bmjopen.bmj.com/site/about/guidelines.xhtml

Health Research Authority

SHORT STUDY TITLE / ACRONYM

Shared decision making for labour and birth

PROTOCOL VERSION NUMBER AND DATE

4.0 09/10/2020

RESEARCH REFERENCE NUMBERS

**IRAS Number:**	**256244**
**SPONSORS Number:**	4656
**FUNDERS Number:**	489

For peer review only

NHS Health Research Authority

SIGNATURE PAGE

The undersigned confirm that the following protocol has been agreed and accepted and that the Chief Investigator agrees to conduct the study in compliance with the approved protocol and will adhere to the principles outlined in the Declaration of Helsinki, the Sponsor's SOPs, and other regulatory requirement.

I agree to ensure that the confidential information contained in this document will not be used for any other purpose other than the evaluation or conduct of the investigation without the prior written consent of the Sponsor

I also confirm that I will make the findings of the study publicly available through publication or other dissemination tools without any unnecessary delay and that an honest accurate and transparent account of the study will be given; and that any discrepancies from the study as planned in this protocol will be explained.

For and on behalf of the Study Sponsor:

Signature: ………………………………………………………………………………………… Date: ……/……/……

Name (please print): …………………………………………………………………………………………

Position: …………………………………………………………………………………………

Chief Investigator:

Signature: ………………………………………………………………………………………… Date: ……/……/……

Name: (please print): …………………………………………………………………………………………

LIST of CONTENTS

GENERAL INFORMATION	Page No.
HRA PROTOCOL COMPLIANCE DECLARATION	i
TITLE PAGE	ii
RESEARCH REFERENCE NUMBERS	ii
SIGNATURE PAGE	iii
LIST OF CONTENTS	iv
KEY STUDY CONTACTS	v
STUDY SUMMARY	v
FUNDING	vi
ROLE OF SPONSOR AND FUNDER	vi
ROLES & RESPONSIBILITIES OF STUDY STEERING GROUPS AND INDIVIDUALS	vi
STUDY FLOW CHART	vii

For peer review only

NHS
Health Research Authority

SECTION	
1. BACKGROUND AND RATIONALE	1
2. THEORETICAL FRAMEWORK	3
3. RESEARCH QUESTION/AIM(S)	3
4. STUDY DESIGN/METHODS	4
5. STUDY SETTING	5
6. SAMPLE AND RECRUITMENT	5
7. ETHICAL AND REGULATORY COMPLIANCE	8
8. DISSEMINATION POLICY	11
9. REFERENCES	11
10. APPENDICES	13

For peer review only

NHS
Health Research Authority

KEY STUDY CONTACTS

Chief Investigator	Dr Anna Davies<br>Department of Women's and Children's Health, Southmead Hospital, The Chilterns, Bristol, BS10 5NB<br>And<br>Centre for Academic Child Health, University of Bristol, Bristol BS8 1NU<br>Tel: 07729229744
Sponsor	North Bristol NHS Trust<br>researchsponsor@nbt.nhs.uk
Funder(s)	David Telling Charitable Trust<br>c/o The Bristol Cancer Institute, Horfield Road, Bristol BS2 8ED.
Key Protocol Contributors	Abi Merriel, abi.merriel@bristol.ac.uk<br>Anna Davies, anna.davies@bristol.ac.uk<br>Sheelagh McGuinness, sheelagh.mcguinness@bristol.ac.uk
Committees	**Core Project Team:**<br>Abi Merriel, Academic Clinical Lecturer University of Bristol<br>Anna Davies, Senior Research Associate, University of Bristol<br>Katie Barnard, Information Specialist, North Bristol NHS Trust<br>Sheelagh McGuinness, Reader in Law, University of Bristol<br><br>**Steering Committee:**<br>Abi Merriel, Academic Clinical Lecturer University of Bristol<br>Anna Davies, Research Associate, University of Bristol<br>Katie Barnard, Information Specialist, North Bristol NHS Trust<br>Sheelagh McGuinness, Reader in Law, University of Bristol<br>Abi Fraser, Reader in Epidemiology, University of Bristol<br>Sonia Barnfield, Head of Obstetrics, North Bristol NHS Trust<br>Danya Bakhbakhi, Research Fellow, University of Bristol<br>Emma Anderson, Research Associate, University of Bristol<br>Kate Birchenall, Research Fellow, University of Bristol<br>Christy Burden, Senior Lecturer in Obstetrics, University of Bristol<br>Rachel Miller, Patient Representative

For peer review only

NHS
Health Research Authority

STUDY SUMMARY

Study Title	Shared Decision making for labour and birth
Internal ref. no. (or short title)	Shared Decision making for labour and birth
Study Design	Qualitative semi-structured interviews and focus groups.
Study Participants	1) Antenatal and postnatal women within 12 months of having a baby.<br>2) Maternity staff working at North Bristol NHS Trust
Planned Size of Sample (if applicable)	1) Up to 40 antenatal and postnatal women<br>2) Up to 32 maternity staff
Follow up duration (if applicable)	For the antenatal women who agree to participate postnatally, up to 4 months
Planned Study Period	February 2020 – October 2020
Research Question/Aim(s)	The aim of this research is to improve shared decision making for labour and birth, with the purpose of improving women's experiences of making decisions during labour and birth. To do so, we would like to carry out exploratory work to elicit the views of antenatal and postnatal women and staff, to inform development of future intervention/s.<br><br>The questions we will seek to answer are:<br>1. How is shared decision making currently experienced by women during labour and birth, and what is the impact of this on their experiences of birth and postnatally?<br>2. What information do women receive about intrapartum interventions, when is it given, and by whom? What impact did it have on the decision-making process during labour?<br>3. What are the experiences of staff in supporting women to make shared decisions?<br>4. What intrapartum interventions do women and staff feel that women should receive information about?<br>5. How do women and staff think information about intrapartum interventions could be given? What information should be given, how, when, and by whom?

FUNDING AND SUPPORT IN KIND

**FUNDER(S)**<br><br>(Names and contact details of ALL organisations providing funding and/or support in kind for this study)	**FINANCIAL AND NON FINANCIALSUPPORT GIVEN**
David Telling Charitable Trust	£21,026.54

For peer review only

c/o The Bristol Cancer Institute, Horfield Road, Bristol BS2 8ED.	

ROLE OF STUDY SPONSOR AND FUNDER

The study sponsor and any funder have played no role in study design, conduct, data analysis and interpretation, manuscript writing, and dissemination of results.

ROLES AND RESPONSIBILITIES OF STUDY MANAGEMENT COMMITEES/GROUPS & INDIVIDUALS

The steering committee has discussed the aims of the study and inputted into the study design. We have two women actively as patient representatives involved in the protocol design and the design of the patient information leaflets.

PROTOCOL CONTRIBUTORS

Dr Abi Merriel, Dr Anna Davies and Dr Sheelagh McGuinness have developed the protocol. The study steering committee have all approved the final protocol patient representatives were involved in the development of the study, and Rachel Miller (patient representative) has inputted to the protocol and the participant information leaflets.

STUDY FLOW CHART

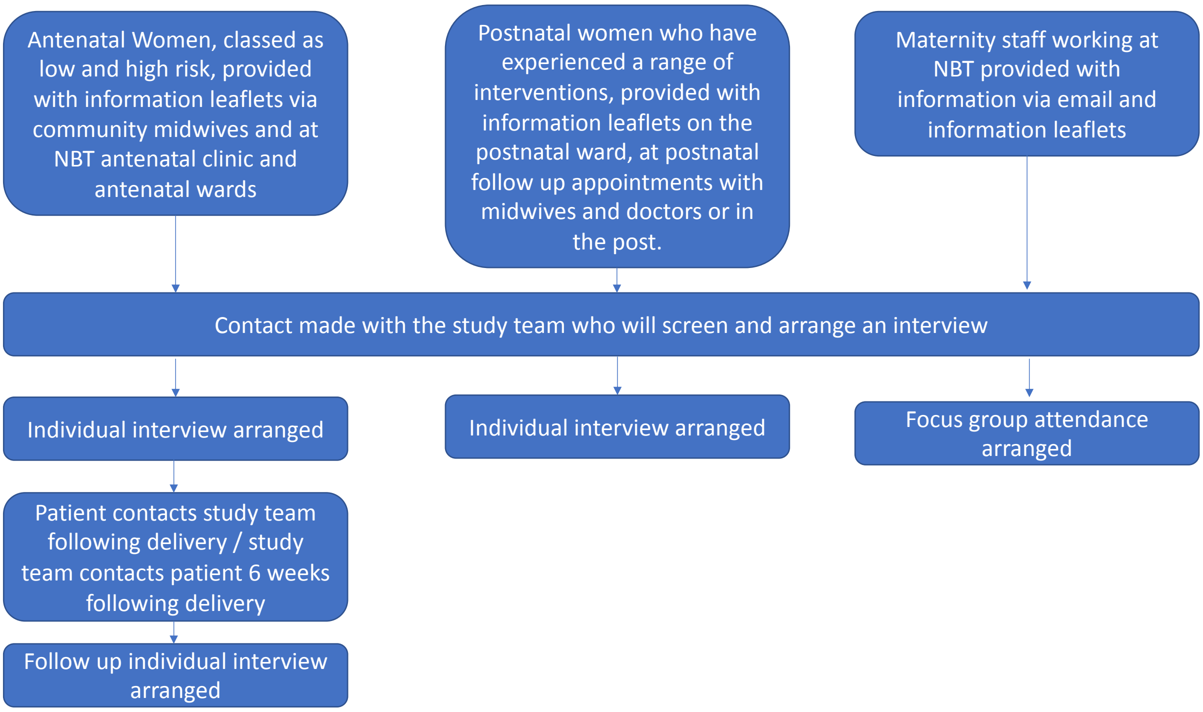

For peer review

NHS
Health Research Authority

STUDY PROTOCOL

1 BACKGROUND AND RATIONALE

Informed consent has become a priority since the Montgomery ruling in 2015 (Chan et al., 2017). A legal challenge confirmed the need to provide women with information that is important to them to enable them to make an informed decision about their care. Alongside this, there is the pressing need to respond to calls from our patients and childbirth charities, to improve experience during birth (Birthrights 2013). This is supported by NICE guidelines, which suggest that alternative ways of supporting women in making informed decisions should be investigated (NICE 2017). Recently, NHS England and NHS improvement have highlighted shared decision making as a cornerstone of the NHS's goal for personalised care (NHS England & NHS improvement 2019). Despite all of this attention, little guidance is provided for clinicians and the 'process' has not evolved greatly beyond the signing of a consent form at or around the time of a procedure.

During labour, women are often offered interventions to ensure their and their baby's safety and to optimise their experience of birth. Women may feel vulnerable and are often in pain, it could be considered to be a particularly difficult time at which to have to make decisions about interventions. It is therefore vitally important that we prepare women for the decisions they may be asked to make in labour and ensure that staff are well equipped to work in partnership with women to make these decisions. This shared decision making (SDM) is the pinnacle of patient centred care, and moves the conversation between a clinician and woman beyond that of 'seeking' informed consent towards a partnership. It involves an exchange of ideas between the patient and clinician, and collaboration in decision-making, based on the patient's views and values (Whitney et al., 2003). It has been defined as '*an approach where clinicians and patients share the best available evidence when faced with the task of making decisions, and where patients are supported to consider options to achieve informed preferences*'. SDM is supported by evidence, it can result in improved outcomes, less regret, better adherence (Stacey et al., 2014) and it can also reduce health inequalities (Durand et al,. 2014).

Impact of informedness on outcomes

Giving women adequate information about intrapartum interventions to enable them to participate in decision making is important for their postnatal psychosocial wellbeing. Women want to participate in decision making during labour(Lavender et al., 1999), they report poorer birth experiences when they have a lack of information, expectations that are not met and feel a lack of control.(Lavender et al., 1999, Green et al., 1990) Surveys undertaken by the charity Birthrights, as well as other published literature, suggest that women who have intrapartum interventions such as instrumental or caesarean delivery have poorer experience of care (Birthrights 2013, O'CAthain et al., 2002, Jackson et al., 2000). A woman's experience of birth can have a significant impact on mental health. Some studies suggest that women undergoing interventional births have a higher incidence of postnatal depression (Boyce & Todd 1992, Hannah et al., 1992). This finding is supported by a meta-analysis which has shown that there is a small impact of obstetric factors on postnatal depression (O'Hara & Swain 1996). Furthermore, negative birth experience can contribute to stress-related symptoms which can be present in up to 50% of women two months after Caesarean section and in 24% at six weeks after vaginal deliveries(Ayers& Pickering 2001). Up to 1.5% of women experience post-traumatic stress disorder (PTSD) at six months following delivery(Ayers& Pickering 2001, Olde et al., 2006). In addition to the mental health implications, the impact of a negative birth experience is associated with women having fewer children, larger birth intervals and infertility (Gottvall & Waldenstrom 2002).

Whilst the causes of a poor birth experience are multifactorial, one important factor associated with poor birth experience is being unprepared for what may happen during labour(Henriksen et al., 2017). The current literature shows that women often do not receive the information needed to make decisions and feel in control during labour. One example is of a qualitative study focused on the labour ward culture and women's acceptance of interventions. The study observed that true choices in labour were limited and identified that informed consent was rare. However, generally women accepted interventions despite lack of information (Marshall et al., 2011). Another study found a similar lack of informedness about common intrapartum interventions. It found that 60% of women

For peer review only - http://bmjopen.bmj.com/site/about/guidelines.xhtml

were not aware of the risks and benefits of a vaginal examination and 34% were not consulted for an episiotomy (Thompson & Miller 2014). This was also investigated in another study where there was variation in feelings of how informed women felt according to which interventions were used. For example, 38% of women felt they made an informed choice about monitoring in labour, whereas 64% felt they were informed about having an epidural (O'Cathain et al., 2002).

Complexities of shared decision-making during labour and birth

A key issue in supporting women to make shared decisions during labour and birth is the urgency of the decision making, and the fact that women are often in pain and tired when making their decisions. Even in non-emergent situations, it is frequently necessary for a woman to make decisions quickly, without much time to consider different options or risks to her or the baby.

Some women, are not adequately prepared women for this potentially complex decision making by current antentatal care and education. It may be that discussions about intervention around the time of birth are minimised to avoid causing any distress to women who are planning and hoping for an unassisted vaginal birth. The Royal College of Obstetricians and Gynaecologists provides guidance about seeking informed consent in the obstetric context, where women in labour may be experiencing pain, and may be under the influence of analgesia (RCOG 2015), though no guidance to support SDM is given. To mitigate the inherent difficulties in achieving informed decisions while in labour, they state that where possible, women should be informed during the antenatal period about predictable problems and interventions that may occur. There is further guidance that women should be given summarised information concerning possible procedures and interventions upon admission in labour or for induction of labour. It is advised that the views of women are sought about such procedures so that their care givers are aware of the choices made by the woman and will act accordingly.

A difficulty with this guidance is that it is not possible to pre-empt every occurrence that will require a decision. Occasionally, immediate lifesaving actions are required, and to maximise the likelihood of good outcomes for the mother and baby there is little time for detailed discussion about risks, benefits and viable alternative courses of action. However, the requirements of consent are not the same in this situation and whether it is possible to achieve SDM in this situation is likely to be debateable (Whitney 2003).

Preparation for intrapartum decision making

Some investigation of how to improve information relating to informed consent around pregnancy has been undertaken, however there has not been a focus on shared decision-making during labour. A systematic review of decision aids for pregnancy care found that decision aids increased knowledge, decreased decisional conflict scores and decreased anxiety (Vlemmix et al., 2013). This suggests that improving how we deliver information can also have a positive impact on women, however, it is unclear whether and to what extent women wish to receive information about interventions during labour antenatally and whether this will help them to make shared decisions with their care providers during their labour and birth.

A key issue for improving experiences of making shared decisions about intrapartum interventions is identifying *what information should be given* (e.g. risks, benefits and reasonable alternatives), about *which interventions*, and *when* those important pieces of information should be given to support recall and use of that information when a decision needs to be made. Challenges relating to this include giving women adequate information about the range of possible interventions at a point when they will be able to process it, for example antenatally or during the early stages of labour, and balancing it with the potential to cause anxiety about labour and birth. Nonetheless, most women will receive at least one procedure for which a shared decision should be made, including vaginal examinations or pain relief. However, if this information about these interventions is not provided until it is required, there may be difficulty in conveying adequate information and providing opportunity to consider it, to support decision making. One example is the risks and benefits of epidurals during labour. A study has found that women wanted to know the possible complications of epidurals, but not their incidence when they were in

For peer review only - http://bmjopen.bmj.com/site/about/guidelines.xhtml

established labour(Jackson et al., 2000). This highlights that when women receive information may impact upon what they want to know, but by not conveying this information staff may be in breach of medicolegal requirements.

The antenatal period does provide the opportunity to deliver information about intrapartum interventions which would reach the majority of women. In the UK women attend ten antenatal appointments with a midwife in their first pregnancy and seven in their pregnancies thereafter. Some also attend additional appointments with doctors. The NICE guidelines state that women should be provided with information about breastfeeding, preparation for labour and birth and awareness of postnatal depression (NICE 2017). The extent to which information about potential interventions for labour and birth are given in antenatal care or during NHS antenatal education is unclear. However, there is no detail about how this information should be delivered, what it should include or when it should be provided.

3 THEORETICAL FRAMEWORK

We will use the Six steps in quality intervention development (6SQuID) framework (Wight et al., 2016) to explore the issues around informed consent for intrapartum interventions to develop an optimised method for delivering information about intrapartum interventions. The 6SQuID framework provides a pragmatic approach to developing interventions and is applicable to this study, with steps 1-2 relating to defining the problem and identifying how to bring about change in the outcome of interest. The six steps are outlined below. This study will focus on the first two steps of the framework to understand the problem from both the patient and staff perspective, and to identify where it may be possible to intervene to improve the experience of intrapartum consent and subsequent outcomes for women.

1. Define and understand the problem and its causes.
2. Clarify which causal or contextual factors are malleable and have greatest scope for change.
3. Identify how to bring about change: the change mechanism.
4. Identify how to deliver the change mechanism
5. Test and refine on small scale.
6. Collect sufficient evidence of effectiveness to justify rigorous evaluation/implementation.

4 RESEARCH QUESTION/AIM(S)

The aim of this research is to improve shared decision making for intrapartum interventions, with the purpose of improving women's experiences of making decisions during labour and birth. To do so, we would like to carry out exploratory work to elicit the views of antenatal and postnatal women and staff, to inform development of future intervention/s.

The questions we will seek to answer are:

1. How is shared decision making currently experienced by women during labour and birth, and what is the impact of this on their experiences of birth and postnatally?
2. What information do women receive about intrapartum interventions, when is it given, and by whom? What impact did it have on the decision-making process during labour?
3. What are the experiences of staff in supporting women to make shared decisions?
    - Sub-question: for which intrapartum interventions do they feel they need to seek a shared decision?
4. What intrapartum interventions do women and staff feel that women should receive information about?
5. How do women and staff think information about intrapartum interventions could be given? What information should be given, how, when, and by whom?

4.1 Objectives

For peer review only - http://bmjopen.bmj.com/site/about/guidelines.xhtml

NHS
Health Research Authority

1) To develop an understanding of women's views of current practices around information provision and shared decision making for intrapartum interventions.
2) To understand healthcare providers' experiences of shared decision making for intrapartum interventions.
3) To develop an understanding of potential methods for intervening to improve shared decision making.

4.2 Outcome

The outcome of this work will be a detailed view about women's and staff's experiences of shared decision making in relation to intrapartum interventions, and potential improvements that could be made. This information will inform the development of a future intervention to improve this process.

5 STUDY DESIGN and METHODS of DATA COLLECTION AND DATA ANALYIS

Design: Two qualitative studies will be conducted. Semi-structured interviews and focus group discussions will be used to understand the decision-making process during labour and how it can be improved. The study design, data collection and methods of analysis are described below for each study.

Study 1:
Aim: To explore antenatal and postnatal women's views of current practices around information provision and shared decision making for intrapartum interventions**,** and their recommendations for how to improve the provision of information to support this throughout pregnancy and during labour.

Participants:
Group 1: Approximately 20 women will be recruited antenatally and asked to participate in an interview pre and post-delivery.
Group 2: Approximately 20 women who have experienced interventions will be recruited postnatally.

Method: Semi structured interviews lasting for approximately 60 minutes will be carried out by a member of the research team trained in qualitative research methods. We are aiming to recruit up to 40 women to the study (up to 20 of which will be interviewed twice) as we believe that this will be provide us with enough different perspectives to provide an understanding of current experience and allow us to identify possible models for provision of information.

A topic guide will be used (Appendix 1). Women from a range of socio-demographic backgrounds, with a breadth of experiences will be purposively sampled and invited to participate in the interviews. They will take place either a North Bristol Trust site, by telephone or at the participant's home if preferred.

After informed written consent is gained (Appendix 2), the interviews will be recorded using an encrypted device and uploaded on to the secure University of Bristol server. The interviews will be transcribed using a University of Bristol approved transcription service, who are subject to a duty of confidentiality.

For women in group 1, the antenatal interviews can take place from 12 weeks gestation to the woman going into labour with their second interview taking place approximately 6 weeks postnatally. We are anticipating that some of these women will choose not to be interviewed for a second time. For the women in group 2, the interview will be arranged between 6 weeks and 6 months postnatally.

Analysis: Interviews will be coded by two members of the research team as they are completed. We will use a framework analysis approach to interrogate the data. Analysis once the interviews are completed will ensure that when saturation of themes is reached, no additional interviews will be conducted unnecessarily. Nvivo software will be used to analyse the interview transcripts.

Study 2:

For peer review only - http://bmjopen.bmj.com/site/about/guidelines.xhtml

Health Research Authority

Aim: To explore healthcare providers' experience of providing information and gaining consent for intrapartum interventions and their recommendations for how to improve the provision of information to support informed consent antenatally and during labour**.**

Method: Approximately four focus groups, with up to 8 participants each, lasting 60-90 minutes will be held. They will be facilitated by two members of the research team, with one facilitating and one taking written notes. They will follow an interview topic guide (Appendix 3). The interviews will take place at a North Bristol NHS Trust site at varying times to accommodate staff working patterns and ensure that those that are willing to take part are able to. Written informed consent (Appendix 4) will be obtained. The groups will be audio recorded onto an encrypted recorder and transcribed by a University of Bristol approved transcription service, who are subject to a duty of confidentiality.

Analysis: A thematic analysis will be used to understand staff perspectives. Data will be analysed using Nvivo software.

6 STUDY SETTING

This study will take place at North Bristol NHS Trust in the Women's and Children's Department, which provides services to women from across Bristol. It is a busy obstetric unit with consultant and midwifery led patients. It will therefore provide the opportunity to invite participants from a range of backgrounds with a range of experiences in terms of their level of risk in their pregnancy and their birth setting.

We will recruit patients from community midwifery and hospital antenatal clinics, the antenatal wards, the post-natal wards and postnatal follow up clinics at Southmead. We will undertake interviews either at women's homes at a time convenient to them, or at Southmead if that is more convenient.

7 SAMPLE AND RECRUITMENT

7.1 Eligibility Criteria

7.1.1 Inclusion criteria

Women:

Group 1 (Antenatal and postnatal paired interviews)

Any woman, who is over the age of 18, who is pregnant beyond 12 weeks of gestation, who is booked for delivery at North Bristol NHS or attending community midwifery services in the North Bristol area. They must be able to speak English.

Group 2 (postnatal interviews)

Any woman who is over the age of 18 and has had a delivery, including some form of intervention at North Bristol NHS trust within the last 12 months, recruited through the Birth Afterthoughts service, and where they have attended for Postnatal Review. We will also recruit through trusted social media channels including facebook and twitter. They must be able to speak English.

Staff:

Any member of staff working in the women's and children's division of North Bristol NHS Trust who is delivering clinical care for women in the intrapartum period. They must be involved or witness the process of giving information relating to clinical procedures and obtaining informed consent for them. We will include both midwives working in the delivery suite setting, and midwives working in the community.

7.1.2 Exclusion criteria

Women: Women who have not or are not about to receive intrapartum care for example women who have had a first trimester miscarriage. Women who are booked for their pregnancy at another NHS trust. Women

For peer review only - http://bmjopen.bmj.com/site/about/guidelines.xhtml

who are under the age of 18. Women who are not able to give informed consent to participate in the study and women who do not speak English adequately to participate in an interview.

Staff: Staff not delivering direct intrapartum clinical care or not working at North Bristol NHS Trust.

7.2 Sampling

7.2.1 Size of sample

Women:
We will recruit up to 40 women to participate in the study split between the two groups mentioned above, a group of antenatal women agreeing to a pre and post birth interview and a group of postnatal women who have experienced interventions. Please note that the sample size for these interviews will be determined by data saturation being reached, whereby interviews in each group of participants will end when no new themes are generated.

Staff:
Up to 32 staff will be recruited to take part in four focus groups of 8 people. The size and number of focus groups was selected to ensure that individual perspectives are captured, from a broad spread of views from across the clinical spectrum, whilst keeping the amount of data manageable within the time and funds available.

7.2.2 Sampling technique

Women:
A purposive approach to sampling will be used to ensure that women across the spectrum on pregnancy and pregnancy experiences are represented. This will include:

- Antenatal Women in their first pregnancy
- Antenatal women in their subsequent pregnancy
- Postnatal women who have had interventions in labour including those who have had postnatal contact with the hospital team due to traumatic events during their birth. Examples of interventions may include (but are not limited to) caesarean section, instrumental delivery, post-partum haemorrhage and manual removal of placenta.

Staff:
A purposive approach to sampling will be taken to ensure that all staff groups are represented:

- Midwives (birthsuite/ homebirth team/delivery suite)
- Maternity Care Assistants
- Obstetricians & Gynaecologists and those in training
- Anaesthetists and Anaesthetic trainees
- Junior doctors (non-specialists)working on the labour ward

7.3 Recruitment

7.3.1 Sample identification

Identification of women
Women from the different groups will be identified in different ways.

1. Antenatal Women in their first and subsequent pregnancies will be identified from community and hospital antenatal clinics, and the antenatal ward. Women will be given an information sheet outlining the purpose of the study by midwifery clinic staff or a member of the research team (see Appendix 5). Women may also be recruited through trusted social media channels. They will be invited to express interest in participation using a reply slip/emailing or by informing the recruiter. These women will be added to an interest log (Appendix 6). Following an expression of interest they will be given a full participant information leaflet (Appendix 7). They will then be contacted by a member of the study team who will

For peer review only - http://bmjopen.bmj.com/site/about/guidelines.xhtml

answer any questions that they have, and will assess them against the eligibility criteria using the screening log (Appendix 8). Eligible participants will be offered an appointment at a convenient time to be interviewed at a North Bristol Trust site, the University of Bristol, by telephone, or at another mutually convenient setting. At the end of the interview we will make individual arrangements of when and how to contact the participant to arrange the follow up interview. For example, whether we will contact them by telephone about 2-3 weeks after their due date. We will arrange that we will contact a woman up to 3 times via telephone to try to arrange their follow up. We may also make individual arrangements with women to send an additional reminder via text message, as this may be easier to respond to at their convenience.

2. Postnatal who have experienced interventions: Postnatal women attending birth afterthoughts appointments or postnatal reviews will be identified from those follow up clinics. Women will also be identified by the community midwifery team and could be recruited through trusted social media channels. An information sheet will be distributed to all women (Appendix 9), who will be invited to contact the study team by email or telephone. Alternatively, women returning a reply slip (Appendix 9) will be contacted by telephone/email by a member of the study team. After receiving a participant information leaflet (Appendix 10). All women will have their questions answered and if they are willing to proceed they will be assessed for eligibility using a screening log (Appendix 8) A convenient time and location for interview will be arranged.

Women will receive up to £15 as a contribution towards their costs and inconvenience for each interview they participate in.

Staff Identification
Staff will be identified through an email advertisement (Appendix 11) sent by an NBT member of staff and word of mouth at NBT. Individuals expressing interest in participating will be added to a screening log (Appendix 12) contacted by a member of the research team, given an information sheet about the study (Appendix 13) and offered a selection of dates to attend a focus group session, so that it is convenient to them.

It is possible that due to Covid restrictions on the size of group meetings focus groups may need to be moved online. In this instance the focus group will be arranged for a convenient time and conducted on trusted videoconferencing software (e.g. MS Teams, Zoom or Skype).

Staff will receive up to £15 as a contribution towards their travel costs and inconvenience.

7.3.2 Consent

Study 1: Women: At the start of the interview, women will be invited to review the information sheet (Appendix 7/10) and discuss any questions they have with the interviewer. Following this written informed consent will be sought using a consent form (Appendix 2). They will then be asked to complete the appropriate demographics form (Appendix 14/15/16)

Where interviews are conducted via telephone, consent will be sought using an online version of the above form (appendix 2). Women will be asked to complete this prior to their interview. If it is not completed beforehand we will complete the consent form verbally at the start of the interview with the participant, and record it. We will complete the demographics form verbally at the start of the interview.

Study 2: Staff: At the start of the focus group, participants will be invited to review the information (Appendix 13) and ask any questions they have of the facilitator. Informed consent will be sought using a consent form (Appendix 4). They will then be asked to complete the demographics form (Appendix 17).

For peer review only - http://bmjopen.bmj.com/site/about/guidelines.xhtml

Health Research Authority

Where it is necessary to conduct the focus group online, staff will be asked to complete the consent and demographics form online before attending.

For peer review only

Health Research Authority

8 ETHICAL AND REGULATORY CONSIDERATIONS

8.1 Assessment and management of risk

Study 1: Interviews with women

Risk	Mitigation
1. Woman reports issues concerning poor practice	All incidents of poor practice identified during an interview will be referred to management. Women will be asked to provide their contact details for further discussion or investigation.<br><br>If the issue is one that may have caused distress but is not a patient safety issue/ poor practice, they will be asked if they would like to raise it formally with North Bristol NHS trust. If they say yes they will be provided with a complaints leaflet to enable them to report the issue if they wish to. (see appendix 18 for Disclosure policy v1.1 29_05_2020)
2. Distress of woman when recounting birth experiences	Women experiencing distress during their interview will be supported using the distress policy. For most women, the interviewer will pause the interview, offer immediate support and check their wellbeing. If the participant feels able to continue their interview it will be resumed.<br><br>For those that are unable to continue the interview will be terminated and the participant will be encouraged to contact a family member, friend or health care provider. The researcher will offer to do so if desired.<br>If the participant is showing signs of distress consistent with harm to self or others, their GP will be contacted immediately, or we will arrange for them to attend hospital immediately to instigate formal support.<br><br>Participants will be followed up with a call if they give permission to be contacted. They will be invited to call a member of the team if they experience distress in the days following the interview.<br><br>(see appendix 19 Distress policy v2.0 29_05_2020)
3. Risk to staff of travel to private home	The first choice interview location will be the Southmead hospital site, by telephone call or videoconferencing.<br>It is very unlikely that staff will travel to participant homes due to current covid-related restrictions.

For peer review only - http://bmjopen.bmj.com/site/about/guidelines.xhtml

	However, if necessary, staff will endeavour to travel in pairs to the homes of participants, otherwise the lone working of the appropriate employer procedure will be followed.
4. Women may have a poor pregnancy outcome between the antenatal and postnatal interview	We do not want to remove the opportunity from women of participating in this study, as that would be removing an opportunity to discuss their experience and acknowledge their grief. However, we will need to sensitively approach women and clearly offering them every opportunity to not complete the interview if this is preferable to them. We will also offer them the opportunity to delay their participation by up to 1 month if this would support their needs.

Study 2: Staff focus groups

**Risk**	**Mitigation**
1. Staff reports issues concerning poor practice	We will have a disclosure policy for clearly dealing with these issues. (appendix 18)<br>If a significant patient safety issue is raised or actions which would result in a disciplinary action are identified, the focus group leader will approach the participant discuss it further.<br>They will explain that as poor practice has been identified that there is a duty to report it to the management team. They will follow North Bristol NHS Trusts internal incident reporting system (Datix) and will report it to the relevant manager.<br><br>If the information is not a significant patient safety issue or an action that would result in a disciplinary action, the participant will be approached. It will be suggested that they can raise the issue with the appropriate manager if they wish to, or in the Datix system. We will also inform them that anonymised feedback will be provided to the department within the context of the study, and therefore anonymised information will be given to the department management team.
2. Staff time away from work to participate	Arranging of focus groups outside of working hours and at times convenient to participants.

8.2 Research Ethics Committee (REC) and other Regulatory review & reports

The study will be performed subject to favourable opinion, authorisation and permission from all necessary regulatory and other bodies. This includes but is not limited to the Health Research Authority (HRA), a UK Research Ethics Committee and the NHS.

This study will be conducted in accordance with:

- International Conference for Harmonisation guidelines for Good Clinical Practice (ICH GCP)

For peer review only

For peer review only - http://bmjopen.bmj.com/site/about/guidelines.xhtml

- UK Policy Framework for Health and Social Care Research

Before commencement the CI will obtain the formal 'go-ahead' from the Host and Sponsor, North Bristol NHS Trust.

If amendments to the protocol and/or the study documents are required, relevant approvals will be sought from the HRA. The CI will be responsible for decisions to amend the protocol and to determine whether the amendment is substantial or non-substantial, in liaison with the Sponsor (NBT). Relevant study documents will be altered, and the changes made tracked to demonstrate where they have been made. These will be submitted to the HRA, along with a supporting letter to the REC and NHS R&D. Amendments to the protocol will be documented using sequential version numbers, with updated documents replaced in the site file.

8.3 Peer review

This study has been reviewed as part of a competitive application process to the David Telling Charitable Foundation.

8.4 Patient & Public Involvement

The study and research questions have been discussed and refined with the two patient representatives on the research team. The protocol and participant facing documentation have been reviewed by these two members of the team.

8.5 Protocol compliance

It is known that accidental protocol deviations can happen. These will be documented on relevant forms and reported to the CI and Sponsor immediately. Causes of deviation will be identified and procedures put in place to mitigate them.

8.5.1 Monitoring

Progress Monitoring

The core project team will meet approximately monthly to ensure the study meets its milestones. The project steering group will meet six monthly after the study opens to recruitment, to examine recruitment rate data, study progress, communication and dissemination plans.

Regulatory Monitoring

The study will be monitored in accordance with NBT's Monitoring Standard Operating Procedure. All study related documents will be made available on request for monitoring and audit by NBT, the HRA or other licensed bodies.

The monitoring plan will be developed and agreed by the Sponsor. Monitoring will take place through assessments conducted by local Sponsor representatives.

This visit will be used to review the completeness and filing/archiving arrangements for: all forms, signature, delegation and accountability logs, investigator site file, evidence of training.

Safety Monitoring

This study is an interview study with no intervention, therefore the risks associated have been documented above and beyond this there are unlikely to be any safety related events.

Provision has been made through the distress policy (appendix 11) of dealing with adverse events, for example, psychological distress. If this distress policy has to be deployed we will inform the sponsor. Participants will be

For peer review only - http://bmjopen.bmj.com/site/about/guidelines.xhtml

asked to report any other adverse events to the study team when they occur. Adverse events will be recorded and reported in accordance with the Sponsor's Safety Reporting Standard Operating Procedure.

For peer review only

For peer review only - http://bmjopen.bmj.com/site/about/guidelines.xhtml

NHS
Health Research Authority

8.5.2 Protocol Violations

Participants who do not follow the planned protocol are considered to have a protocol violation. The nature of the protocol violation will be documented for each participant.

8.5.3 Withdrawal

Participants are free to withdraw from the study at any point up until the data analysis begins, after which time it will not be possible to dis-aggregate their data from that of others. This will not affect their ongoing care. If the woman withdraws from the study, she will be managed by the clinical team as per routine care guidelines. If a member of staff withdraws from the Study this will not affect their working role(s).

A record will be kept of participants who withdrew consent on a specific Study Withdrawal Form (SWF). This will allow the participant to specify what level of data already collected they are happy to be used in the study analysis (i.e. no data, data collected to date).

8.6 Data protection and patient confidentiality

Participant data will include:
Study 1: Semi structured interview audio files (electronic) and demographic data (paper), consent forms,. Transcripts of audio recordings
Study 2: Focus group audio files (electronic) and demographic data (paper), consent forms, transcripts of audio recordings,

Paper data: Consent forms will be stored for the duration of the study in the NBT research offices in the women's and children's department. The demographic data collected will be entered into an excel spreadsheet and the paper copy destroyed. A participant identifier will be allocated to all participants in the study, and an excel data file will link participant details to their identifier. The master list linking participant names and Participant IDs will be kept on University of Bristol Computer in a password protected file.

Electronic data (audio files): Audio recorded data will be downloaded to an UoB secure computer and encrypted password protected files will be used. Once download is completed, the file will be deleted from the audio recording device. This data will then be transferred to a UoB approved provider for transcription services using a secure transfer facility. The provider is subject to a duty of confidentiality. Following transcription the data will be transferred back to a UoB secure computer and saved in an encrypted, password protected file.

Data will be collected and retained in accordance with the General Data Protection Regulation (GDPR) (EU) 2016/679 and within the principles of Good Clinical Practice. Participant data collection forms will be labelled with the Participant ID and the participants' hospital identification label.

Personal data will be treated in strict confidence. Participants' personal information, including name, address, telephone number and email address is required in order for the research team to keep in contact with participants for the duration of the research. Participants will be advised that if they wish to check what personal data the Study team are holding for this research, they should contact the CI.

Aside from any planned contact, outlined in this protocol, the only time the research team would use personal data would be if there was a concern that a participant was at risk of harm due to their involvement in the study. If there was a need to inform relevant authorities of risks to participants, the study team would discuss this first with participants and encourage them to seek appropriate help.

Research data will be anonymised before analysis. Participants will not be able to withdraw research data once the process of analysis has commenced.

Data Storage

For peer review only - http://bmjopen.bmj.com/site/about/guidelines.xhtml

The Study data will be stored on secure servers at NBT and UoB with daily backup. Study documents (paper and electronic) will be retained in a secure location during, and 10 years after, the study. After this time, it will be safely disposed of. All essential documents, including patient records and other source documents will be retained in accordance with NBT's Archiving Standard Operating Procedure following the end of a study. The electronic records on UoB servers will be retained for 10 years.

8.7 Indemnity

This is an NHS-sponsored research study. For NHS sponsored research HSG(96)48 reference no.2 refers. If there is negligent harm during the clinical trial when the NHS body owes a duty of care to the person harmed, NHS indemnity covers NHS staff, medical academic staff with honorary contracts, and those conducting the trial. NHS indemnity does not offer no-fault compensation and is unable to agree in advance to pay compensation for non-negligent harm.

8.8 Access to the final study dataset

The final study data set will be stored on the NBT and UoB Secure Servers and on. All members of the core study team will have access to this data. Individuals in the wider steering group will receive parts of the data, as required, for analysis and discussion, and can access the full dataset by visiting either UoB or NBT.

It is not envisaged that this data will be used for analysis outside the remit of this study, however, we will ask permission to use the data for subsequent analysis in the consent form.

9 DISSEMINATION POLICY

9.1 Dissemination policy

9.1.1 Intellectual Property

Intellectual Property that will be generated (Foreground IP) comprises of copyright in the following items:

- Study protocol
- Other publications
- Presentations

All foreground IP will be owned by NBT with an appropriate licence to the collaborating parties for non-commercial research, training and teaching purposes.

9.2 Dissemination Plans

Communication and dissemination will be an agenda item at study team meetings to ensure the identification of appropriate dissemination strategies to maximise potential uptake of the research findings. A publication plan will be also be developed and be subject to ongoing refinement as this study progresses. We will provide a synopsis for this study on our NBT Study web page. We will also invite participants to be part of a mailing list and provide brief accessible reports via a study newsletter. We will submit to national conferences to disseminate our work and engage our peers for any future implementation or larger studies.

Our collaborative links will allow us to expand the reach of our findings and engage with the wider research community, key stakeholders, peers, patients and the public. Furthermore, we will liaise closely with our patient representatives to ensure our findings are accessible to a wide audience. We will co-produce and disseminate a short, briefing paper.

On completion of the Study, the data will be analysed and reported back to the Funder, HRA and REC.

In all publications we will acknowledge The David Telling Charity as the funder, NBT as the Sponsor and UoB as partners.

For peer review only - http://bmjopen.bmj.com/site/about/guidelines.xhtml

Health Research Authority

As this is a qualitative study, we do not intend on making the dataset publicly accessible, however, if other researchers/quality improvement teams request the data we will be happy to share it with them.

9.3 Authorship eligibility guidelines and any intended use of professional writers

Authorship will be granted to all members of the study team who participate in the design of the study, data collection or analysis of results and contribute to the final paper.

10 REFERENCES

Ayers S, Pickering AD. Do Women Get Posttraumatic Stress Disorder as a Result of Childbirth? A Prospective Study of Incidence. *Birth* 2001;28(2):111-118

Birthrights. *Dignity in Childbirth: The Dignity Survey 2013: Women's and midwives' experiences of dignity in UK maternity care.* 2013 Available from http://www.birthrights.org.uk/wordpress/wp-content/uploads/2013/10/Birthrights-Dignity-Survey.pdf [Accessed on 27th January 2018]

Boyce PM, Todd AL. Increased risk of postnatal depression after emergency caesarean section. Med.J.Aust. 1992;157;172-174.

Chan SW, Tulloch E, Cooper S, Smith A, Wokcik W, Norman JE. Montgomery and informed consent: where are we now? BMJ 2017;357:j2224

Durand, M.A., Carpenter, L., Dolan, H., *et al.* (2014), Do interventions designed to support shared decision-making reduce health inequalities? A systematic review and meta-analysis. PLoS One 9(4):e94670

Gottvall K, Waldenstrom U. Does a traumatic birth experience have an impact on future reproduction? *BJOG* 2002;109: 254–260

Green JM, Coupland VA, Kitzinger JV. Expectations, Experiences, and Psychological Outcomes of Childbirth: A Prospective Study of 825 Women. Birth 1990; 17 (1): 15-24

Hannah P, Adams D, Lee A, Glover V, Sandler M. Links between early post-partum mood

and post-natal depression. British Journal of Psychiatry. 1992; 160;777-780.

Henriksen L, Grimsrud E, Schei B, Lukasse M on behalf of the Bidens Study Group. Factors related to a negative birth experience – A mixed methods study. *Midwifery* 2017; 51:33–39

Jackson A, Henry R, Avery N, Van Den Kerkhof E, Milne B. Informed consent for labour epidurals: what labouring women want to know. Can J Anesth 2000;47(11):1068-73.

Lavender T, Walkinshaw SA, Walton I. A prospective study of women's views of factors contributing to a positive birth experience. Midwifery 1999:15:40-46

Marshall JE, Fraser DM, Baker PN. An observational study to explore the power and effect of the labor ward culture on consent to intrapartum procedures. Int J of Childbirth. 2011;1(2);82-89

National Institute for Health and Care Excellence (2017) Antenatal care for uncomplicated pregnancies. NICE guideline (CG 62)

NHS England and NHS Improvement. Shared Decision Making: A Summary Guide. 2019

O'Cathain A, Thomas K, Walters SJ, Nicholl J, Kirkham M. Women's perceptions of informed choice in maternity care. Midwifery 2002; 18: 136-144.

O'Hara MW, Swain AM. Rates and risk of postpartum depression-a meta-analysis. International Review of Psychiatry.1996;8:37-54.

Olde E, van der Hart O, Kleber R, van Son M. Posttraumatic stress following childbirth: A review. *Clinical Psychology Review* 2006; 26:1–16

For peer review only - http://bmjopen.bmj.com/site/about/guidelines.xhtml

NHS Health Research Authority

RCOG 2015. Obtaining Valid Consent. Clinical Governance Advice No.6. https://www.rcog.org.uk/globalassets/documents/guidelines/clinical-governance-advice/cga6.pdf

Stacey, D. *et al.* (2014), Decision aids for people facing health treatment or screening decisions, Cochrane Database Syst. Review (1): CD001431

Thompson R, Miller YD. Birth control: to what extent do women report being informed and involved in decisions about pregnancy and birth procedures? BMC Pregnancy and Childbirth 2014; 14 (62).

Vlemmix F, Warendorf JK, Rosman AN, Kok M, Mol BWJ, Morris JM, Nassar N. Decision aids to improve informed decision-making in pregnancy care: a systematic review. BJOG 2013; 120:257-266

Whitney SN. A New Model of Medical Decisions: Exploring the Limits of Shared Decision Making. MEDICAL DECISION MAKING 2003 Jul-Aug;23(4):275-80.

Wight D, Wimbush E, Jepson R, Doi L. Six Steps in quality intervention development (6SQuID). J Epidemiol Community Health 2016;70:520-525

For peer review only

NHS
Health Research Authority

11. APPENDICIES

Appendix 1: Women interview topic guide
Appendix 2: Consent form Women
Appendix 3: Staff focus group topic guide
Appendix 4: Consent Staff Focus groups
Appendix 5: Brief study information antenatal women
Appendix 6: Interest log women
Appendix 7: PIL women - antenatal
Appendix 8: Eligibility and demographic form women.
Appendix 9: Brief information sheet and reply slip postnatal women
Appendix 10: PIL women - postnatal
Appendix 11: Recruitment email for staff focus groups
Appendix 12: Staff screening log focus groups
Appendix 13: PIL Staff
Appendix 14: Initial questionnaire antenatal women
Appendix 15: Postnatal follow up questionnaire for antenatal women
Appendix 16: Initial questionnaire for postnatal only women
Appendix 17: Staff demographic forms
Appendix 18: Disclosure policy
Appendix 19: Distress policy
Appendix 20: Amendment History
Appendix 21: Social Media Adverts
Appendix 22: Study withdrawal form

For peer review only

Supplementary table 2, Standards for Reporting Qualitative Research (SRQR) Checklist(1)

No.	Topic	Item
**Title and abstract**		
S1	**Title**	See title.
S2	**Abstract**	See abstract.
**Introduction**		
S3	**Problem formulation**	See introduction.
S4	**Purpose or research question**	We aimed to understand MCHP's experience of decision-making from a multi-disciplinary perspective in maternity care as a foundation to develop interventions to improve practice.
**Methods**		
S5	**Qualitative approach and research paradigm**	Reflexive thematic analysis using an experiential approach. See methods section.
S6	**Researcher characteristics and reflexivity**	See research team and reflexivity.
S7	**Context**	Online focus groups - see data collection.
S8	**Sampling strategy**	Until no new themes emerged, or data saturation was reached. See participant selection, sampling and sample size.
S9	**Ethical issues pertaining to human subjects**	See details of Ethics approval at end of manuscript, participants selection, sampling and sample size, and data collection.
S10	**Data collection methods**	See data collection.
S11	**Data collection instruments and technologies**	See data collection.
S12	**Units of study**	See results, 1st paragraph.
S13	**Data processing**	See data collection , and participants selection, sample and size.
S14	**Data analysis**	See data analysis heading.
S15	**Techniques to enhance trustworthiness**	Member checking, see data analysis heading.

For peer review only

**Results/findings**		
S16	**Synthesis and interpretation**	Main findings, themes and subthemes in results section.
S17	**Links to empirical data**	Quotes integrated throughout results section.
**Discussion**		
S18	**Integration with prior work, implications, transferability, and contribution(s) to the field**	See discussion, beginning paragraph and interpretation in context.
S19	**Limitations**	See strengths and limitations.
**Other**		
S20	**Conflicts of interest**	See disclosure of interests
S21	**Funding**	See funding

1. O'Brien BC, Harris IB, Beckman TJ, Reed DA, Cook DA. Standards for Reporting Qualitative Research: A Synthesis of Recommendations. Academic Medicine. 2014;89(9).

For peer review only

Supplementary Table 3, A phased approach to reflexive thematic analysis(1, 2)

	Phase	Description of activities
1	Familiarisation with data	Immersion within the data was achieved by reading, and re-reading the data set and referring to the field notes associated with each focus group.
2	Generate initial codes	The dataset was coded using succinct, shorthand descriptive labels.
3	Generate themes	Initial themes were formed by identifying patterns within the coded dataset.
4	Review themes	A recursive review of candidate themes was performed.
5	Define themes	The data set and coded data items were re-read to ensure that candidate themes functioned as meaningful interpretation of the data.
6	Write up	An illustrative narrative analysis of each theme was undertaken. A final review of theme name and order was undertaken to ensure the themes and key messages reflected the data, and answer the research question in a logical and clear manner.

1. Braun V, Clarke V. Reflecting on reflexive thematic analysis. Qualitative Research in Sport, Exercise and Health. 2019;11(4):589-97.
2. Byrne D. A worked example of Braun and Clarke's approach to reflexive thematic analysis. Quality & Quantity. 2021.

For peer review only

Supplementary Table 4, Focus groups by participants and experience in maternity care

Group number	Participants in group	Number of participants	Experience in maternity care (years)
1	Midwife, community and integrated care	3	5 - 35
2	Midwife, hospital working in birth centre	3	6.5 - 17
3	Consultant and associated specialist anaesthetists	3	12 - 30
4	Midwife, community, integrated care and diabetes	3	4 - 30
5	Midwife, hospital working in central delivery suite	3	13 - 19
6	Consultant, Obstetrician	5	11 - 20
7	Trainee, Obstetrician and Gynaecologist (ST3-ST7)	4	3.5 - 9
Total		24	

For peer review only

Supplementary Table 5. Theme 1: Contextualising decision-making
Subtheme 1.1. "I keep coming back to time."
lets explore that further, and what's concerning you, and what's led to that decision so far, so that we can make sure that it's the right decision for you. TO, P22.
So our discussion starts with "*Tell me about what you want your experience to be?...Tell me about what you're planning?"*...So it's very much a - their decision making can't happen without the information that I'm going to give them, but equally, I'm taking into account information they're giving me to help them come to a conclusion that works for them... BCMW, P4.
I keep coming back to time, maybe this is ....what I keep coming back to, but you know, it's time to process. Process that information, and then come to as [name] said, you know, what might not necessarily be what we think is the right decision, from our perspective, but when it comes to the patient, and you're bringing all that information together, they feel that's the right decision for them. TO, P22
the midwives that we all work with are incredibly stressed, underfunded, under great time pressures, and there are not enough of them to do the work that is required and the population is increasing and their workload is increasing. TO, P23.
And I feel like shared decision making is something that we all aspire to in situations where we as clinicians feel that there is enough time...TO, P24.
That's why women aren't given information, we have 20 minutes to do so many things...CMW, P11.
So great [name] that you've managed to find space for someone, in a quite a complex situation, but for the majority of women, it's a very superficial process... So we really need to improve that for, you know, for every woman. IMW, P3
you are very much trying to limit the consultation based on the time that you're given for that woman. So I would say that when I'm allowed, longer time with a woman, I would think it was a more informed decision that was going to come out of that because I have time to listen. CO, P17.
Subtheme 1.2: Intrapartum decision-making
there are times...that it isn't always possible to give them ...accommodate them having a discussion about something sometimes you do have to make more...more channelled decision making. BCMW, P5.
you have to we, we have to, and also the birth educators that we currently have in this country have to start having conversation from the very first antenatal class that they hold. BCMW, P6.
at the point where they're in... the process of the labour...that too much choice at that point is actually really derailing. And then I felt like I've left conversations thinking, why did I even? Why did I even do that to that poor woman? ..Like she's now on the edge to a really traumatic experience, because I've given her those choices and tried to say, look, there are other ways you can do X, Y, and Z... BCMW, P6.
how then are we expecting women to be ready to make decisions when they've actually not made a decision at all throughout the whole process of the nine months prior to that. So the whole time when they're meant to be training almost for the event of... Trying to make the shared decisions. We've not given them any training time. Instead, what you say is "*Okay, at the point of birth, then you get choices*". But actually, at the point of birth, the choices go from nothing to a million and one choices. BCMW, P6.
So let's prepare them for the main options, and then train them to be fluid, you know, so that then they have a slightly more open minded, kind of coming into it. BCMW, P6
Subtheme 1.3: Variation in practice
I think there's just such a massive variety of sources of information that women receive and I don't think there's a huge amount of standardisation. TO, P22
every single situation, every woman is different. Every doctor is different, every interaction is different. All you can do is keep honing your skills, practising and doing your best. There's no, I don't think there's one way that definitely isn't one way of doing it...A, P8.
But again, I think the whole consent thing is it is you tailor it to the patient... So it's very hard to say "*this is the way you should be imparting that information*". A, P9.

For peer review only

[we] will communicate with the same person in a different way, depending on the situation. A,P7.
We are all different people…we're better off getting a broad experience of seeing how different people do these things in order to work out actually what would work for our particular communication style or personality to try and keep things as, as shared and as broad as possible. TO, P22.
sometimes women get less a whole lot less from me, than perhaps they should because I'm tired and rushed. A, P9.
So I think every single woman you tailor what you say differently. It's all according to like you say what, or how, or your perception of their understanding as well. IMW, P12.
there are days when you're better at it, then there are days when you think, *"Oh, God, I could have done that better"* CMW, P11.
I just think in general, communication is something that we bang on about all the time and you do it, you know…everyone's saying "*you know communication's key*", but actually, the communication isn't always there. IMW, P3
skills around the actual conversation could be improved… you're making me think there's some teaching sessions we could be doing here. A, P9
I'd like a trainee to sit in clinic, and [name] trained in [place] as I did, and there was consultant there, who actually, came and sat in with you in clinic, and he sat there with you while you consulted, and by golly, your consultation style, improved, your feedback, etc… I think hands on direct, consultant, training like that, is really important. CO, 17.
Subtheme 1.4: Accessibility of decision-making conversations
whilst we want to give women this information, to try and empower them, and hopefully make things better, that I think there will be a group of patients who who will, they won't want that information, because they'll find it potentially very scary, or, you know that, but certainly, it might put some barriers up to accepting that information. TO, P22.
they don't realise that they can discuss that option. So I think when when, when you present them with an opportunity to discuss this, whatever problem they might have, um they're quite welcoming... I think sometimes they're quite surprised that that actually can happen, that they can discuss.... Whatever point they've they've come across with somebody. CMW, P2.
It's almost like continuing to give them permission that they can say what they feel, or they can say what they want or, you know, and then ...so there's that in the process of continuing to say, "*You have choice, this isn't prescription*" BCMW, P6
I think using an interpreter for people with a language barrier has a profound impact on trying to communicate in an emergency or even semi emergency situation. If I have someone on the labour ward who in any way might need a caesarean, sometimes in the middle of night, I find it quite useful to go in and go through a consent form with a translator in advance of doing a procedure because I think for those women communicating with them is so incredibly difficult... I think undoubtedly those women making intrapartum decision making is like the dreaded decision making because it's a difficult thing to communicate if both people have a shared first language, let alone with a language line and a phone interpreter and a possible partner. I think that's just like a perfect storm of issues. .TO, P21.
you've got the whole range of the tertiary level educated patient who doesn't want us to do anything versus quite often someone who maybe left school after GCSEs...but you still have to provide both of those sets of patients with all the same information, but you have to then guide how you do that. And that's, that can be quite challenging. TO, P22.

For peer review only

**Supplementary Table 6. Theme 2: Controversies in current decision-making practices.**
Subtheme 2.1. Clinical expertise and personal experience in decision making
So what you would tend to do in that situation is probably stress the the downsides of having a general anaesthetic and talk about actually, you know, failed intubation ...So actually, we will manipulate that conversation based on us...thinking we actually probably do know the best thing for that patient. A, P8.
Yeah, exactly like a midwife, led unit midwife describing a breech in [place] where they do it quite frequently-ish, versus like a consultant who works in HCIB describing it at [place], that is a very different description that you will receive. TO, P21
We do all subconsciously do that, we select which bits of information we think the patient needs. A, P9.
Can you distinguish bias from experience? Or from or from teaching? I suppose, in that we're coming from a clinical viewpoint where we're...How do I put this? ...I'm trying to sort of say that it's not necessarily a biased opinion. Whereas I suppose what I'm trying to get at is that as a hopefully an experienced clinician, is it still bias? TO, P22
And so like if I don't want to induce a patient at 37 weeks for a pretty benign reason, but the patient is really keen to be induced, I will give them the figures for nicu admission, whereas if there's a patient who I want to induce, I might not necessarily tell them that same information. TO, P23.
Because we all know that, you know, with risks and percentages and risk ratios, etc, you can you can lean any decision to different ways. CO, P19.
I sort of feel like women are very coerced. ..And I feel like the information that's shared with women isn't neutral. They're scared into stuff. CMW, P12.
you acknowledge your bias and say, "*Well, obviously, I'm a consultant obstetrician, and I see, you know, a lot of high risk, and therefore I am biased*" CO, P18.
[the BRAIN app] is really good because it gives a really good balance and what are the risks, what are the benefits, what are the alternatives, what are the family's preferences. So it just it's a really good tool for facilitating those shared decisions, and looking at other people's perspectives as well. CMW, P2.
I do think sometimes putting numbers on things [by using absolute risk rather than relative risk] does help to give a kind of a more fair picture and allow people to make decisions that are maybe, well you know, just informs them and then they can make the decision they feel is right for them. TO, P24.
Subtheme 2.2. Conditions limiting validity of consent
I think in an emergency situation, I find it very difficult , because I think the consent process I currently go through seems like a bit of a sham... we go through this process of waving a consent form at them saying "*you and your baby going to die. If we don't do this*". TO, P21.
when you're trying to consent a labouring woman for an epidural, and she's screaming, "*just put it in*" at you that, you know, they don't take on board, we could tell them the risks were, you know, "*1 in 2 risk of death*" or something, at that point, they're not listening to you at all. A,P8.
we cannot say that a woman in labour is giving true consent, even for an epidural, when she has so much pain...She's so crippled and tired and, you know, fed up with everything, that she'll just agree to anything. IMW, P12.
I've never seen anyone try and do a decision making kind of conversation at the time of a shoulder dystocia, and I've also never come across a mum who has retrospectively said, "*I can't believe you didn't talk to me about that first*." TO, P24
"*Why was it five minutes before they took me around to theatre that somebody suddenly mentioned that I might end up having a caesarean section, why? When there were nine months when I could have been counselled about this*." TO, P21

For peer review only

We've got sort of risks of general anaesthesia, regional anaesthesia, we've got these lovely information cards from the OAA (Obstetric Anaesthetists' Association). A, P22
Subtheme 2.3. Challenges faced when women/birthing people decline medical advice
she knew the risk, but she was absolutely clear what the risks were, what the implications could be what the outcome could be for her baby, but, that was the decision that she wanted. And it's it was so difficult. IMW, P3.
I think it is the fear of, of litigation, and that defensive practice, which is the overwhelming you know, feeling. I know, I've had some personal experiences around that. So that definitely does probably change the way I practice as a midwife, making me perhaps more overcautious… it's that kind of fear of, if something happens or goes wrong the responsibility then lies with you as the midwife, and the woman…will turn around and say, "*Well, that was something that you didn't do,*" Or "*if you'd have told me something differently, that wouldn't have happened*.". BCMW, P5.
they won't let you deliver that baby. And I find that always challenging and it takes maybe 12, 24 or 48 hours before you're allowed to do that. And then that baby obviously has, may have problems. And they're the ones I really struggle with…and it gets turned very much back against you as the medical professional saying, "*Why didn't you explain that this might happen*?" Even if it's been written in black and white. TO, P23
Yeah, it's, it's massive that and um what support networks are in there? Because at the end of the day, you still got another, you know, 50 women on your caseload that you've got to look after. IMW, P1
You need to talk about every option, not just the one you want them to do, and so you need to be really facetious about it.. It's a nightmare...CO, P18
if you give women too much information, you're just scare mongering, you know, if I say "*you've got this percentage chance and this percentage or whatever*". So it is difficult. CO, P19
the trace was pretty horrid... And she really did need to have a caesarean section, but she'd made the decision... And, and that's what we did. And I actually felt although it was, it wasn't a pleasant experience, it actually, for me, it was positive, because I know that we'd I'd worked with both with the couple as much as I possibly could. DSMW, P15
It's like women who decline induction, it's like, well, we'll tell you about the risks again, because you aren't doing what we've decided is the right thing to do from our perspective of, you know, recommendations. *"Remember, it's on you now*"...You know, and therefore, it's not shared decision making. BCMW, P6.
That might be because we've alienated people as well. So I think with with regards to pre birthing, and birthing outside of guidance. CO, P19.
they were they were quite bullish, actually in the hospital, they kept ringing her but she just turned the phone off in the end and said," I'm not going to speak to you, I need a day off from all of you. CMW, P11.

For peer review only

A qualitative study of maternity healthcare professionals' experiences of supporting women in decision-making for labour and birth.

Journal:	*BMJ Open*
Manuscript ID	bmjopen-2023-080961.R1
Article Type:	Original research
Date Submitted by the Author:	01-Mar-2024

Complete List of Authors:	Hardman, Kitty; University of Bristol, Bristol Medical School; North Bristol NHS Trust<br>Davies, Anna; University of Bristol, Centre for Academic Child Health<br>Demetri, Andrew; University of Bristol, Bristol Medical School; North Bristol NHS Trust, Department of Women's and Children's Health<br>Clayton, Gemma; University of Bristol, Faculty of Health Sciences<br>Bakhbakhi, Danya; University of Bristol, Centre for Academic Women's Health<br>Birchenall, Katherine; University of Bristol, Bristol Medical School<br>Barnfield, Sonia; North Bristol NHS Trust, Department of Women's and Children's Health<br>Fraser, Abigail; University of Bristol, School of Social and Community Medicine<br>Burden, Christy; University of Bristol Faculty of Health Sciences<br>McGuinness, Sheelagh ; University of Bristol, Law; University of Bristol Law School<br>Miller, Rachel; Independent patient representative<br>Merriel, Abi; University of Bristol, Bristol Medical School; University of Liverpool, Institute of Life Course and Medical Sciences, Department of Women's and Children's Health, Centre for Women's Health Research

**Primary Subject Heading**:	Obstetrics and gynaecology
Secondary Subject Heading:	Communication, Patient-centred medicine, Qualitative research
Keywords:	Decision Making, OBSTETRICS, QUALITATIVE RESEARCH, Clinical Decision-Making

SCHOLARONE™ Manuscripts

I, the Submitting Author has the right to grant and does grant on behalf of all authors of the Work (as defined in the below author licence), an exclusive licence and/or a non-exclusive licence for contributions from authors who are: i) UK Crown employees; ii) where BMJ has agreed a CC-BY licence shall apply, and/or iii) in accordance with the terms applicable for US Federal Government officers or employees acting as part of their official duties; on a worldwide, perpetual, irrevocable, royalty-free basis to BMJ Publishing Group Ltd ("BMJ") its licensees and where the relevant Journal is co-owned by BMJ to the co-owners of the Journal, to publish the Work in this journal and any other BMJ products and to exploit all rights, as set out in our licence.

The Submitting Author accepts and understands that any supply made under these terms is made by BMJ to the Submitting Author unless you are acting as an employee on behalf of your employer or a postgraduate student of an affiliated institution which is paying any applicable article publishing charge ("APC") for Open Access articles. Where the Submitting Author wishes to make the Work available on an Open Access basis (and intends to pay the relevant APC), the terms of reuse of such Open Access shall be governed by a Creative Commons licence – details of these licences and which Creative Commons licence will apply to this Work are set out in our licence referred to above.

Other than as permitted in any relevant BMJ Author's Self Archiving Policies, I confirm this Work has not been accepted for publication elsewhere, is not being considered for publication elsewhere and does not duplicate material already published. I confirm all authors consent to publication of this Work and authorise the granting of this licence.

For peer review only

Title page

A qualitative study of maternity healthcare professionals' experiences of supporting women in decision-making for labour and birth.

Authors:

Kitty Hardman[1,2], Anna Davies[1,], Andrew Demetri[1,2], Gemma Clayton[1], Danya Bakhbakhi[1], Katherine Birchenall,[1] Sonia Barnfield[2], Abigail Fraser[1], Christy Burden[1,2], Sheelagh McGuinness[3], Rachel Miller[4], Abi Merriel.[1,5]

1. [1]University of Bristol Medical School, University of Bristol, UK
2. [2]Department of Women's and Children's Health, North Bristol NHS Trust, UK
3. [3]University of Bristol Law School, University of Bristol, UK
4. [4]Independent patient representative
5. [5]Centre for Women's Health Research, Department of Women's and Children's Health, Institute of Life Course and Medical Sciences, University of Liverpool, UK

Corresponding author: Dr Abi Merriel, abi.merriel@liverpool.ac.uk, https://orcid.org/0000-0003-0352-2106

Address: Centre for Women's Health Research, Department of Women's and Children's Health, Institute of Life Course and Medical Sciences, Faculty of Health & Life Sciences, University of Liverpool, Liverpool Women's Hospital, Crown Street, L8 7SS

For peer review only

For peer review only - http://bmjopen.bmj.com/site/about/guidelines.xhtml

ABSTRACT

Objectives

To explore and characterise maternity healthcare professionals' (MHCPs) experience and practice of shared decision-making (SDM), to inform policy, research, and practice development.

Design

Qualitative focus group study.

Setting

Large Maternity Unit in the Southwest of England.

Participants

MHCPs who give information relating to clinical procedures and pregnancy care relating to labour and birth and are directly involved in decision-making conversations were purposively sampled to ensure representation across MHCP groups.

Data collection: A semi-structured topic guide was used.

Data Analysis: Reflexive thematic analysis was undertaken.

Results

Seven focus groups were conducted, comprising a total of 24 participants (3-5 per group). Two themes were developed: *contextualising decision-making* and *controversies in current decision-making*. Contextual factors that influenced decision-making practices included lack of time, and challenges faced in intrapartum care. MHCPs reported variation in how they approach decision-making conversations and asked for more training on how to consistently achieve SDM. There were communication challenges with women who do not speak

For peer review only

For peer review only - http://bmjopen.bmj.com/site/about/guidelines.xhtml

English. Three controversies were explored: the role of prior clinical experience, the validity of informed consent when women were in pain and during life-threatening emergencies, and instances where women declined medical advice.

Conclusions

We found that MHCPs are committed to SDM but need better support to deliver it. Structured processes including Core Information Sets, communication skills training and decision support aids may help to consistently deliver SDM in maternity care.

Strengths and limitations

- Multi-disciplinary perspective: community, integrated care, diabetes specialist, birth centre and delivery suite midwives, consultant and trainee obstetricians and specialist associate and consultant anaesthetists.
- Moderated focus group study design enabling generation of rich data.
- Online setting allowing safe collection of data during COVID-19 pandemic.
- Limited to single healthcare trust.
- Limited to MHCPs' perspectives.

For peer review only - http://bmjopen.bmj.com/site/about/guidelines.xhtml

For peer review only

INTRODUCTION

Shared decision-making (SDM) is fundamental to clinical practice in obstetrics (1, 2, 3, 4, 5, 6). It is a process where the woman is at the centre of her care and is able to share information regarding decision-making preferences, personal values and beliefs, and where the maternity healthcare professional (MHCP) provides information about benefits and risks of management options to enable an autonomous, informed decision (4, 5, 7). Informed consent (IC), often the endpoint of SDM, is where the woman makes an informed, voluntary choice of treatment, and is often symbolised by signing a consent form(3, 8). MHCPs are legally bound to achieve IC prior to providing treatment (4, 8).

Various terminology has been used to describe decision-making practices, including SDM, informed decision making, and supported decision making (5, 9). This research follows UK General Medical Council (GMC), National Health Service (NHS), and Royal College of Obstetricians and Gynaecologists (RCOG) guidance on decision-making and consent (4, 10, 11) and therefore uses the term SDM to ensure understanding and comparison. However it is recognised that the word "shared" may fail to acknowledge women as the ultimate decision-makers (12, 13). We have referred to "woman/women." Other parents and families use different words and we respect their chosen terminology. An ongoing dialogue between health care systems and the patients they serve is required to ensure patient centred terminology.

SDM is an international healthcare priority (3, 14, 15). It provides short- and long-term benefits through improved birth experiences, satisfaction with care regardless of outcome, improved maternal mental health outcomes, reduced pre-term birth, higher birth weights, and enables safer care (16, 17, 18, 19, 20). Failing to involve women can lead to their feeling out of control and powerless, and is associated with negative and traumatic birth experiences, increased rates of postnatal depression, anxiety and PTSD (19, 21).

Decision-making occurs throughout pregnancy. However, achieving intrapartum and emergency SDM poses unique challenges: women may be in pain, tired, scared, under the influence of opiate analgesia, or all of the above (22, 23). In addition they often have limited time to consider available options and the risks posed to

For peer review only - http://bmjopen.bmj.com/site/about/guidelines.xhtml

themselves or their baby (22). Frequently cited barriers to practising SDM are time pressures and lack of clinical applicability, i.e. a belief that SDM is inappropriate in that clinical situation (24).

Despite these challenges, the Royal Colleges of Emergency Medicine, Obstetrics and Gynaecology and Midwifery provide limited emergency specific SDM guidance(5, 7, 25). The GMC advise taking a proportional approach to emergency decision-making, leaving MHCPs to subjectively interpret best practice. Given limited guidance and challenges posed by emergency care, it is unsurprising that emergency obstetric interventions confer the greatest sense of perceived loss of control and choice, and are associated with the poorest psychosocial outcomes (26, 27).

Guidance is needed for SDM in maternity, and especially intrapartum care, to achieve better psychosocial outcomes for women and to support MHCPs (17). We aimed to understand MCHP's experience of decision-making from a multi-disciplinary perspective in maternity care as a foundation to develop interventions to improve practice.

METHODS

The standards for reporting qualitative research (SRQR), checklist guided reporting of this study (28) (supplementary file, S1).

Patient and public involvement

A patient representative was a member of the project steering committee and contributed to protocol design.

Research team and reflexivity

The research team comprised obstetricians (KH, AD, DB, KB, SB, CB, AM) , a research psychologist (AKD), patient representative (RM), a lawyer (SM), information specialist (KB) and epidemiologist (AF). Data collection and analysis was carried out by KH, AKD, AD and AM. KH and AD are trainee obstetricians and

For peer review only

early career academics, AKD, a research psychologist, with qualitative and maternity research experience, AM is an academic obstetrician. Whilst the professional background of the researchers (KH, AD) enabled "fitting in" with participants (29), we considered possible over-representation of the MHCP perspective, therefore a second non- MHCP moderator attended focus groups and multidisciplinary discussion of candidate themes was undertaken by the research team.

Study design

Moderated focus groups explored MHCPs' experiences of SDM. Focus groups provide an open, supportive environment that facilitate in-depth discussions about sensitive and personal topics, leading to new and unexpected knowledge (30).

Participant selection, sampling and sample size

Participants were purposively sampled from a single NHS trust in the south-west of England, with 6,000 deliveries annually. All MHCPs directly involved in clinical decision-making conversations with women were considered for inclusion. We targeted: midwives (community, integrated care, birth centre and delivery suite) and doctors (consultant and trainee obstetricians and anaesthetists). Focus groups included between three and five participants to ensure each person had the opportunity to contribute. Potential participants were approached via email, posters, and word of mouth. Participant information leaflets were emailed to interested participants and remote informed consent, demographic data, and anonymised record ID numbers were generated and recorded using REDCap (31, 32). Participants received a £10 e-voucher. Recruitment continued until data was said to be "saturated" (33).

Data collection

Moderated focus groups were held online in July 2021.The primary moderator (KH) asked questions, whilst the second moderator (AKD/AD) took field notes. A topic guide (supplementary file, S2) structured the discussions but once they begun, the natural flow was not interrupted. Questions relating to: experiences

For peer review only - http://bmjopen.bmj.com/site/about/guidelines.xhtml

For peer review only

and challenges of supporting women in decision-making, perceptions of maternal preparedness for labour and birth, and ways in which decision-making for labour and birth could be improved were asked. An encrypted audio recording device was used, audio-recordings were transcribed verbatim, and uploaded into NVivo (34).

Data analysis

An experiential orientation to data interpretation was adopted. Meaning was derived through personal experiences, and how individuals process these experiences (35). Reflexive thematic analysis was undertaken using an iterative process (supplementary file, S3) (36, 37). An inductive (bottom-up) approach was taken, whereby codes and themes were directly linked to the data, however a degree of deductive (top-down) analysis was used to ensure the research question remained at the fore (37, 38). Each phase was carried out independently and then discussed collaboratively (KH, AKD and AM).

Trustworthiness

The presence of a second moderator through all focus groups enabled field notes to be taken throughout. The re-reading of these notes alongside audio recordings ensured non-verbal communication, and subtleties of communication were captured enabling an accurate interpretation of what was said, and triangulation of data sources.

After each focus group, moderators would reflect on how the focus group had run, and discuss initial interpretation of the data, enabling immediate assumptions to be challenged and discussed. A continuous and “prolonged engagement” of reflection and discussion was maintained throughout the study (39, 40) (S3). Data saturation was reached once researchers independently agreed that sufficient “thick” and “rich” data had been achieved, and no new codes or themes emerged through group discussion (40).

For peer review only - http://bmjopen.bmj.com/site/about/guidelines.xhtml

RESULTS

Seven focus groups, each with 3-5 participants per group (24 participants in total) were conducted in July 2021 (see Table 1 below). Participants included community (CMW), integrated care and diabetes specialist midwives (IMW), birth centre (BCMW) and delivery suite (DSMW) midwives, trainee (TO) and consultant obstetricians (CO), and specialist associate and consultant anaesthetists (A).

Table 1, Focus groups by participants and experience in maternity care.

Focus group	Participants in group	Participants (n)	Experience in maternity care (years)
1	Midwife, community and integrated care	3	5 - 35
2	Midwife, hospital working in birth centre	3	6.5 - 17
3	Anaesthetist, consultant and associated specialist	3	12 - 30
4	Midwife, community, integrated care and diabetes specialist	3	4 - 30
5	Midwife, hospital working in delivery suite	3	13 - 19
6	Obstetrician and Gynaecologist, Consultant	5	11 - 20
7	Obstetrician and Gynaecologist, Trainee	4	3.5 - 9
Total		24	

Two overarching themes were developed, theme 1 *Contextualising decision-making*, and theme 2 *Controversies in current decision-making practices.* Figure 1 illustrates each theme and component subthemes. Select quotations supporting each theme are presented in Tables 2 and 3, with full list of quotations in supplementary files S4, S5. Each theme and subtheme will be discussed in turn.

Figure 1, Themes and subthemes

Theme 1: Contextualising decision-making

For peer review only - http://bmjopen.bmj.com/site/about/guidelines.xhtml

Participants identified systemic barriers to SDM. They felt there was not enough time to adequately discuss management options, and described limiting the discussion to the time available. Participants felt more time could enable better discussions. MHCPs reported significant variation in their individual approach to SDM. Decision-making conversations varied depending on the individual needs, and preferences of the woman.

Subtheme 1.1: Not enough time

All groups felt their ability to achieve SDM was related to the time available, "I keep coming back to time… when you've got that time to actually provide some information" (TO, P22).

Having time enabled MHCPs to perform high-quality decision-making, which involved exploring preferences, addressing fears and building trusting relationships. However, the time routinely available was felt to be insufficient, "you are very much trying to limit the consultation based on the time that you're given for that woman. So I would say that when I'm allowed longer time with a woman I would think it was a more informed decision that was going to come out of that because I have time to listen" (CO, P17).

Experienced community midwives felt that systemic changes including reduced appointment times contributed to poorer SDM, "we used to spend hours sitting with every single woman before she delivered doing the birth plan, but it wasn't really the birth plan, it was a birth discussion…so she could tell you all her fears, and that would help with her decision-making process" (IMW, P1).

Participants described good decision-making experiences to involve multiple or longer appointments to build rapport, so women could process information and deliberate decisions, however this was not the norm. For example, when a CMW described using multiple longer antenatal appointments another participant replied, " So great [name] that you've managed to find space for someone, in a quite a complex situation, but for the *majority* of women, it's a very superficial process… So we really need to improve that for, you know, for every woman" (IMW, P3).

Subtheme 1.2: Intrapartum decision-making

For peer review only - http://bmjopen.bmj.com/site/about/guidelines.xhtml

SDM during labour was challenging, women were felt to have limited capacity to engage in conversations due to pain, fatigue, and feeling scared and that decisions were time dependent, “you've not got time to just pause the body, give everyone a break, give the woman 25 minutes to process the information” (BSMW, P6). Presenting women with new information and multiple options during labour can be “derailing” and “traumatising”. SDM approaches adopted in the antenatal setting were sometimes felt to be ineffective in achieving a meaningful, informed conversation.

Groups emphasized the need for improved antenatal education so that women arrived at the point of birth informed of the main options, and knowledgeable of their preferences, so that discussions in labour did not require giving new information, or unexpected choices.

Subtheme 1.3: Variation in practice

Decision-making practice varied within and between MHCPs. Communication skills and how information was imparted to women varied with time, the decision, the patient, and the clinician, “every single situation, every woman is different, every doctor is different, every interaction is different. All you can do is keep honing your skills, practising and doing your best. [There] definitely isn't one way of doing it...” (A,P8)

All groups described an ad-hoc approach to developing communication skills for decision-making, with none receiving formal training. The obstetrician and anaesthetist groups discussed the importance of learning from senior clinicians, “seeing how different people do these things in order to work out actually what would work for our particular communication style or personality to try and keep things as, as shared and as broad as possible” (TO, 22). The midwifery groups reported fewer on-the-job learning opportunities. Participants felt communication skills training would improve consultations.

All participants reported variation in their ability to achieve SDM, “there are days when you're better at it, then there are days when you think, “*Oh, God, I could have done that better*” (CMW, P11). Factors such as

For peer review only

For peer review only - http://bmjopen.bmj.com/site/about/guidelines.xhtml

fatigue, hunger and stress were felt to also contribute to how well they carried out SDM, "sometimes women get less a whole lot less from me, than perhaps they should because I'm tired and rushed" (A, P9).

Subtheme 1.4: Adapting to the individual needs and preferences of the woman

There was perceived variation in the individual needs, and decision-making preferences of women. Most notably, women who did not speak English faced barriers to communication, and pre-emptive conversations in these situations were felt to be essential to ensure understanding and consent, "I think using an interpreter for people with a language barrier has a profound impact on trying to communicate in an emergency or even semi emergency situation…I find it quite useful to go in and go through a consent form with a translator in advance of doing a procedure because I think for those women communicating with them is so incredibly difficult" (TO, P21).

Others highlighted the challenges of ensuring patients from all socio-demographic backgrounds were equally informed, "you've got the whole range of the tertiary level educated patient who doesn't want us to do anything versus quite often someone who maybe left school after GCSEs...but you still have to provide both of those sets of patients with all the same information…that can be quite challenging" (TO, P22).

Lastly, women were perceived to vary in the amount of information they wanted to receive, and their role in decision-making, some seeming to prefer clinician-led decision making, "You know, there are some people who say, *"I don't want to know, I don't want to know the risks of this, I just want you to do what you think I need"*….but I've got to tell you...it says here I have to consent.. I have to tell you all the risks, and they don't want to know" (A, P8).

For peer review only

224 *Table 2, Theme 1 Contextualising decision-making.*

**Theme 1: Contextualising decision-making**
Subtheme 1.1. Not enough time
I keep coming back to time....what I keep coming back to, but you know, it's time to process. Process that information, and then come to as [name] said, you know, what might not necessarily be what we think is the right decision, from our perspective, but when it comes to the patient, and you're bringing all that information together, they feel that's the right decision for them. TO, P22
The midwives that we all work with are incredibly stressed, underfunded, under great time pressures, and there are not enough of them to do the work that is required and the population is increasing and their workload is increasing. TO, P23.
And I feel like shared decision making is something that we all aspire to in situations where we as clinicians feel that there is enough time.TO, P24.
Subtheme 1.2: Intrapartum decision-making
There are times that it isn't always possible to give them ...accommodate them having a discussion about something sometimes you do have to make...more channelled decision making. BCMW, P5.
You have to we, we have to, and also the birth educators that we currently have in this country have to start having conversation from the very first antenatal class that they hold. BCMW, P6.
At the point where they're in... the process of the labour...that too much choice at that point is actually really derailing. And then I felt like I've left conversations thinking, why did I even? Why did I even do that to that poor woman? Like she's now on the edge to a really traumatic experience, because I've given her those choices and tried to say, look, there are other ways you can do X, Y, and Z. BCMW, P6.
Subtheme 1.3: Variation in practice
I think there's just such a massive variety of sources of information that women receive and I don't think there's a huge amount of standardisation. TO, P22
So I think every single woman you tailor what you say differently. It's all according to like you say what, or how, or your perception of their understanding as well. IMW, P12.
There are days when you're better at it, then there are days when you think, "*Oh, God, I could have done that better*." CMW, P11.
Communication is something that we bang on about all the time and you do it, you know...everyone's saying "*you know communication's key*", but actually, the communication isn't always there. IMW, P3
Subtheme 1.4: Adapting to the individual needs and preferences of the woman
Whilst we want to give women this information, to try and empower them, and hopefully make things better, that I think there will be a group of patients who who will, they won't want that information, because they'll find it potentially very scary, or, you know that, but certainly, it might put some barriers up to accepting that information. TO, P22.
I think using an interpreter for people with a language barrier has a profound impact on trying to communicate in an emergency or even semi emergency situation. If I have someone on the labour ward who in any way might need a caesarean, sometimes in the middle of night, I find it quite useful to go in and go through a consent form with a translator in advance of doing a procedure because I think for those women communicating with them is so incredibly difficult.TO, P21.
You've got the whole range of the tertiary level educated patient who doesn't want us to do anything versus quite often someone who maybe left school after GCSEs...but you still have to provide both of those sets of patients with all the same information, but you have to then guide how you do that. And that's, that can be quite challenging. TO, P22.

225

226

For peer review only - http://bmjopen.bmj.com/site/about/guidelines.xhtml

Theme 2: Controversies in current decision-making practices.

Theme 2 explores controversies in current decision-making practices: participants reported providing information in a way that aligned with their clinical perspective; pain and emergency situations were felt to limit the validity of IC; women declining medical advice was challenging and MHCPs were fearful of medicolegal repercussions, whilst these women were made to feel isolated.

Subtheme 2.1: Clinical expertise and personal experience in decision-making

All groups reported bringing their clinical expertise, training and experience to decision-making conversations, resulting in women receiving differing information from different MHCPs, "a [midwife] describing a breech where they do it quite frequently-ish, versus like a consultant who works in HSIB [Healthcare Safety Investigation Branch] that is a very different description that you will receive" (TO, P21).

Two distinct issues became apparent. First, the way in which MHCPs conducted decision-making conversations and the information provided to women was influenced by training, experience and individual interpretation of the available evidence, and was described by some as their personal or clinical bias. This was felt to be very difficult to mitigate.

Second, there were occasions where participants felt that they presented information differently depending on the particular clinical situation, "so like if I don't want to induce a patient at 37 weeks for a pretty benign reason, but the patient is really keen to be induced, I will give them the figures for NICU admission, whereas if there's a patient who I want to induce, I might not necessarily tell them that same information" (TO, P23). This practice was reported most amongst the obstetrician and anaesthetist groups.

Some participants had insight about their potential clinical biases. They discussed the importance of using absolute rather than relative risk and infographics to help communicate information objectively. Two participants described using a decision tool to help standardise information.

For peer review only - http://bmjopen.bmj.com/site/about/guidelines.xhtml

Subtheme 2.2: Conditions limiting the validity of consent

MHCPs believed that severe pain and life-threatening emergencies meant it was near-impossible to achieve IC, let alone SDM.

The anaesthetic and midwifery groups felt that many women were unable to weigh up risks and benefits of an epidural when they were in so much pain, "when you're trying to consent a labouring woman for an epidural, and she's screaming, "*just put it in!*" …we could tell them the risks were, you know, 1 in 2 risk of death or something, at that point, they're not listening to you at all" (A,P8). The anaesthetic group felt pre-emptive conversations regarding epidural analgesia were important, and reported using information cards to support this.

All groups questioned whether SDM and IC is possible in life-threatening situations. A trainee obstetrician reported, "I've never seen anyone try and do a decision-making kind of conversation at the time of a shoulder dystocia, and I've also never come across a mum who has retrospectively said, "*I can't believe you didn't talk to me about that first" (*TO, P24). However they reported following process and signing consent forms, despite feeling it doesn't reflect SDM or IC. A trainee obstetrician reported, "I think in an emergency situation, I find it very difficult , because I think the consent process I currently go through seems like a bit of a sham… we go through this process of waving a consent form at them saying, "*you and your baby going to die if we don't do this* " (TO, P21).

Subtheme 2.3: Challenges faced when women decline medical advice

Decision-making conversations are challenging when women decline medical advice. MHCPs were psychologically affected by poor neonatal and maternal outcomes and fearful of medicolegal repercussions, "it gets turned very much back against you as the medical professional saying, *Why didn't you explain that this might happen?* Even if it's been written in black and white…" (TO,P23).

For peer review only - http://bmjopen.bmj.com/site/about/guidelines.xhtml

Several participants reported practising more defensively having experienced poor neonatal outcomes. Furthermore, participants from all groups felt they needed to explain all risks to women to protect themselves against litigation, "it's that kind of fear of, if something happens or goes wrong the responsibility then lies with you as the midwife, and the woman…will turn around and say, "*Well, that was something that you didn't do,*" Or "*if you'd have told me something differently, that wouldn't have happened*."" BCMW, P5

However, this approach was perceived to infringe on women's experience of decision-making. For example, women were reportedly harassed when they declined medical advice, and made to feel that their decisions were not respected by repeatedly being told the risks of declining medical advice, or being repeatedly offered medical interventions. A community midwife described a woman having to turn her phone off to avoid repeated phone calls offering her an induction of labour.

For peer review only - http://bmjopen.bmj.com/site/about/guidelines.xhtml

Table 3, Theme 2: Controversies in current decision-making practices.

**Table 2, Theme 2: Controversies in current decision-making practices.**
Subtheme 2.1. Clinical expertise and personal experience in decision making
So what you would tend to do in that situation is probably stress the the downsides of having a general anaesthetic and talk about actually, you know, failed intubation ...So actually, we will manipulate that conversation based on us...thinking we actually probably do know the best thing for that patient. A, P8.
We do all subconsciously do that, we select which bits of information we think the patient needs. A, P9.
I sort of feel like women are very coerced. ..And I feel like the information that's shared with women isn't neutral. They're scared into stuff. CMW, P12.
[The BRAIN app] is really good because it gives a really good balance and what are the risks, what are the benefits, what are the alternatives, what are the family's preferences. So it just it's a really good tool for facilitating those shared decisions, and looking at other people's perspectives as well. CMW, P2.
Subtheme 2.2. Conditions limiting validity of consent
I think in an emergency situation, I find it very difficult , because I think the consent process I currently go through seems like a bit of a sham… we go through this process of waving a consent form at them saying "*you and your baby going to die. If we don't do this*". TO, P21.
When you're trying to consent a labouring woman for an epidural, and she's screaming, "*just put it in*" at you that, you know, they don't take on board, we could tell them the risks were, you know, 1 in 2 risk of death or something, at that point, they're not listening to you at all. A,P8.
We cannot say that a woman in labour is giving true consent, even for an epidural, when she has so much pain…She's so crippled and tired and, you know, fed up with everything, that she'll just agree to anything. IMW, P12.
I've never seen anyone try and do a decision making kind of conversation at the time of a shoulder dystocia, and I've also never come across a mum who has retrospectively said, "*I can't believe you didn't talk to me about that first*." TO, P24
Subtheme 2.3. Challenges faced when women decline medical advice
She knew the risk, but she was absolutely clear what the risks were, what the implications could be what the outcome could be for her baby, but, that was the decision that she wanted. And it's it was so difficult. IMW, P3.
I think it is the fear of, of litigation, and that defensive practice, which is the overwhelming you know, feeling. I know, I've had some personal experiences around that. So that definitely does probably change the way I practice as a midwife, making me perhaps more overcautious…it's that kind of fear of, if something happens or goes wrong the responsibility then lies with you as the midwife, and the woman…will turn around and say, "*Well, that was something that you didn't do,*" Or "*if you'd have told me something differently, that wouldn't have happened*.". BCMW, P5.
It's like women who decline induction, it's like, well, we'll tell you about the risks again, because you aren't doing what we've decided is the right thing to do from our perspective of, you know, recommendations. *"Remember, it's on you now*"...You know, and therefore, it's not shared decision making. BCMW, P6.
That might be because we've alienated people as well. So I think with with regards to pre birthing, and birthing outside of guidance. CO, P19.
They were they were quite bullish, actually in the hospital, they kept ringing her but she just turned the phone off in the end and said, "*I'm not going to speak to you, I need a day off from all of you*". CMW, P11.

For peer review only

DISCUSSION

In this qualitative exploration of MHCPs' experiences of decision-making, participants were motivated to involve women in decision-making. However, challenges to SDM included: time pressures, lack of training and intrapartum/emergency care. MHCPs perceived that women's decision-making needs and preferences varied, non-English speaking women faced communication challenges. Suggested changes to improve SDM were: increased consultation time, skills training, and improved antenatal education. Three areas of controversy were explored: the role of prior clinical experience in SDM, the validity of IC during intrapartum/emergency care, and when women declined medical advice.

The need to deliver patient-centred care, with time to ask questions, express concerns and receive high-quality information coincides with increasing demands on healthcare systems (3, 4, 5, 41). A systematic review of decision-making found that time constraints are the most commonly cited barrier across cultural and organisational contexts (24). For SDM to be successfully implemented a systems approach needs to be considered to provide clinicians with time and resources to counsel women (15).

Decision aids can support MHCPs to standardise content, support risk communication, facilitate discussion about what matters patients, and reduce decisional conflict without extending consultations (42, 43, 44). In UK maternity care, use of decision aids is growing with tools to provide decision-making structure, (10) support discussion about mode of birth, (45) and intrapartum decision making (46).

However, it is unlikely that there can be a decision aid for every decision, and they are not universally acceptable or useful (47). One effective way of improving decision-making skills for clinicians is to role play different decision options alongside the integration of decision aids (47, 48). The NHS personalised care plan expects clinicians to be trained in decision-making conversations (15), however, none of our participants had formal SDM training. MHCPs need to be equipped with the tools to support SDM, and given the opportunity to attend training.

For peer review only

For peer review only - http://bmjopen.bmj.com/site/about/guidelines.xhtml

Language poses a significant barrier to SDM. Women who do not speak the local language face issues around communication and this may affect quality of care (49, 50). The National Institute for Health and Care Excellence (NICE) emphasises the importance of using clear language with resources translated into other languages if needed (50, 51). Our participants had developed strategies to manage decision-making in this group; it is important that the maternity system develops a strategy to support these vulnerable women.

Participants' prior experiences influenced their communication, and in some instances the decision chosen by the woman. These findings are in keeping with research from a range of specialities (26, 52). MHCPs have a duty to declare personal beliefs and potential biases to ensure transparency however, how often this happens in reality is unclear (4, 53). The use of decision aids may help to standardise information, and free it from clinicians' personal biases (42).

In instances where women declined medical advice, participants expressed conflict between fear of litigation and patient autonomy. MHCPs may try to persuade women to accept medical interventions as there is a common belief that they may incur ethical or legal liability if women decline (54, 55). However, this persuasion could negate consent as voluntary choice has been lost (54). MHCPs should be supported to explore the values underlying a woman's refusal, whilst emphasising patient choice. They should be enabled to maintain communication to facilitate safest possible care (26), and in doing so support women to be autonomous decision-makers, and exercise their right to informed refusal of care. Structured, informed refusal processes may help MHCPs feel more confident in caring for these patients, and prevent women from feeling ostracised from medical care (54, 56).

Participants questioned the validity of consent when women were in pain, and during emergencies. Procedures relating to IC were perceived to become meaningless paperwork rather than respectful support and autonomy. Women consented in an emergency are more likely to feel that they would have signed whatever was on the consent form, find the consent form harder to understand and are less likely to remember signing it, and their overall satisfaction with the consent process is lower (57). Focusing on

For peer review only - http://bmjopen.bmj.com/site/about/guidelines.xhtml

332 obtaining written consent in emergency scenarios may not achieve either informed choice or woman-
333 centred care (7). Better birth preparation may improve this.

334 Participants suggested that presenting new information in labour can be overwhelming. Whilst one cannot
335 legislate for every eventuality, women should be aware of common obstetric interventions (58). Improving
336 antenatal education and preparation for birth is vital to improving birth experiences (19, 54, 59), and should
337 be consistently delivered throughout pregnancy to enable SDM (10, 53). The development of Core
338 Information Sets regarding vaginal birth, unplanned assisted birth and unplanned emergency caesarean
339 births offer one way which may help women to receive consistent, accurate information, that is valued by
340 them (60, 61, 62), whilst the use of decision aids may help to standardise and guide decision-making
341 conversations (63).

342 **Strengths and limitations**

343 Further research could involve participant recruitment from additional healthcare trusts and geographically
344 and socially diverse areas. However, our findings are congruent with decision-making experiences across
345 maternity settings, suggesting these results may be relevant more broadly.

346 The online focus groups enabled the study to proceed during the COVID-19 pandemic, they created a relaxed
347 atmosphere and enabled open discussion (30). However technical issues introduced additional challenges.
348 Gaining perspectives of women's experience of SDM is essential and work undertaken to address this is
349 currently being analysed.

350 **CONCLUSION**

351 To improve women's birth experiences, and to better support MHCPs, a systems-wide approach to SDM
352 must be considered. Women require access to information and support throughout pregnancy to ensure
353 they are prepared for decision-making in labour and birth, including familiarisation of common emergency
354 obstetric interventions, and the possibility of unexpected choices. The development of Core Information

For peer review only - http://bmjopen.bmj.com/site/about/guidelines.xhtml

Sets, better support tools, and training for staff will help women to receive balanced information, relevant to them. MHCPs must be supported in providing advice and care to women birthing outside of guidelines with well-defined pathways for those who decline medical advice. Women must be supported to be autonomous decision-makers, including those who choose informed refusal of care. Decision-making and consent during intrapartum and emergency situations should be revisited given the concerns regarding its validity. MHCPs believe in SDM. It is important that research, training, and policy matures alongside health systems to deliver SDM to all women throughout their pregnancy journey.

Acknowledgements

We thank all participants who contributed to the study.

Disclosure of Interests

None

Contribution to Authorship

KH contributed to the design of the study, data acquisition, data interpretation, drafting of the article and with the final approval to be published.

AKD contributed to the conception and design of study, acquisition of data, analysis, interpretation of data, drafting the article and with the final approval to be published.

AD, contributed to the acquisition of data, and final approval of the article to be published.

AM conceived the study and design, contributed to the acquisition of data, analysis, interpretation of data, drafting the article and final approval to be published.

GC, DB, KB, SB, AF, CB, SM, RM contributed to study conception and design, and critical review of the final draft.

For peer review only

For peer review only - http://bmjopen.bmj.com/site/about/guidelines.xhtml

Details of Ethics Approval

20/SW/0035

South West – Frenchay Research Ethics Committee

Funding

This work was supported by a David Telling Charitable Trust award [project 489], and the Elizabeth Blackwell Institute, University of Bristol through the Wellcome Trust [Grant number - 204813/Z/16/Z]. For the purpose of Open Access, the author has applied a CC BY public copyright licence to any Author Accepted Manuscript version arising from this submission.

Data availability

Available upon reasonable request and subject to relevant ethical approvals.

References

1. NHS England. National maternity review. Better Births: Improving outcomes of maternity services in England - A Five Year Forward View for maternity care. London: NHS England; 2016.
2. NHS. Personalised care and support planning guidance. Guidance for local maternity systems.; 2021.
3. Informed consent and shared decision making in obstetrics and gynaecology. ACOG committee opinion. Number 819 (*Replaces Committee Opinion Number 363, April 2007, and Committee Opinion Number 439, August 2009*) [press release]. ACOG2021.
4. GMC. Guidance on professional standards and ethics for doctors. Decision making and consent: General Medical Council; 2020. Available from: https://www.gmc-uk.org/ethical-guidance/ethical-guidance-for-doctors/decision-making-and-consent.
5. Royal College of Midwives. Informed decision making.: Royal College of Midwives; 2022. Available from: https://www.rcm.org.uk/media/6007/informed-decision-making_0604.pdf.
6. Campbell M. Montgomery v Lanarkshire Health Board. Common Law World Review. 2015;44(3):222–8.
7. Royal College of Obstetricians and Gynaecologists. Obtaining Valid Consent. Clinical Governance Advice No.6. London: Royal College of Obstetricians and Gynaecologists; 2015.
8. Department of Health and Social Care. Reference guide to consent for examination or treatment. London: Department of Health; 2009. Available from: www.dh.gov.uk/publications
9. RCS Professional and Clinical Standards. A guide to good practice. Consent: Supported decision making.: Royal College of Surgeons; 2018.
10. NHS. Shared Decision Making Summary Guide: NHS England and NHS Improvement; 2019. Available from: https://www.england.nhs.uk/shared-decision-making/.

For peer review only

11. Royal College of Obstetricians and Gynaecologists. Managing the birth of extremely premature babies is complex and requires shared decision making: Royal College of Obstetricians and Gynaecologists; 2014 [Available from: https://www.rcog.org.uk/en/news/managing-the-birth-of-extremely-premature-babies-is-complex-and-requires-shared-decision-making/.

12. Birthrights. Birthrights and GMC - what does informed consent mean in maternity care? : Birthrights; 2021 [Available from: https://www.birthrights.org.uk/2021/09/17/birthrights-and-gmc-what-does-informed-consent-mean-in-maternity-care/.

13. Women's need must always be at centre of care says RCM in informed decision making briefing for midwives [press release]. RCM2022.

14. World Health Organization (WHO). WHO global strategy on integrated people-centred health services 2016-2026. Switzerland: World Health Organization 2015. Available from: https://www.who.int/health-topics/integrated-people-centered-care#tab=tab_1.

15. NHS. Universal Personalised Care. Implementing the Comprehensive Model. 2019.

16. Care Quality Commission. Getting safer faster: key areas for improvement in maternity services. Available at: Safety, equity and engagement in maternity services: Care Quality Commission (www.cqc.org.uk); 2021. Available from: https://www.cqc.org.uk/publications/themed-work/getting-safer-faster-key-areas-improvement-maternity-services.

17. Independent Maternity Review. Ockenden report – Final: Findings, conclusions, and essential actions from the independent review of maternity services at the Shrewsbury and Telford Hospital NHS Trust. Department of Health and Social Care; 2022 June, 6th, 2022.

18. Garcia ER, Yim IS. A systematic review of concepts related to women's empowerment in the perinatal period and their associations with perinatal depressive symptoms and premature birth. BMC Pregnancy and Childbirth. 2017;17(2):347.

19. Elmir R, Schmied V, Wilkes L, Jackson D. Women's perceptions and experiences of a traumatic birth: a meta-ethnography. Journal of Advanced Nursing. 2010;66(10):2142-53.

20. Nieuwenhuijze MJ, Korstjens I, de Jonge A, de Vries R, Lagro-Janssen A. On speaking terms: a Delphi study on shared decision-making in maternity care. BMC Pregnancy and Childbirth. 2014;14(223).

21. Beck CT. Birth trauma: in the eye of the beholder. Nurs Res. 2004;53(1):28-35.

22. Vernon G, Alfirevic Z, Weeks A. Issues of informed consent for intrapartum trials: a suggested consent pathway from the experience of the Release trial [ISRCTN13204258]. Trials. 2006;7:13.

23. Alvarez M, Hotton E, Harding S, Ives J, Crofts J, Wade J. Women's and midwives' views on the optimum process for informed consent for research in a feasibility study involving an intrapartum intervention: a qualitative study. Pilot and Feasibility Studies. 2023;9.

24. Légaré F, Ratté S, Gravel K, Graham ID. Barriers and facilitators to implementing shared decision-making in clinical practice: Update of a systematic review of health professionals' perceptions. Patient Education and Counseling. 2008;73(3):526-35.

25. The Royal College of Emergency Medicine. Consent in Adults, Adolescents and Children in Emergency Departments: The Royal College of Emergency Medicine; 2018.

26. Birthrights. Dignity in Childbirth: The Dignity Survey 2013: Women's and midwives' experiences of dignity in UK maternity care.; 2013.

27. Yuill C, McCourt C, Cheyne H, Leister N. Women's experiences of decision-making and informed choice about pregnancy and birth care: a systematic review and meta-synthesis of qualitative research. BMC Pregnancy and Childbirth. 2020;20(1):343.

28. O'Brien BC, Harris IB, Beckman TJ, Reed DA, Cook DA. Standards for Reporting Qualitative Research: A Synthesis of Recommendations. Academic Medicine. 2014;89(9).

29. Patton MQ. Enhancing the quality and credibility of qualitative analysis. Health Serv Res. 1999;34(5 Pt 2):1189-208.

30. Wilkinson S. Focus groups in health research : exploring the meanings of health and illness. Journal of health psychology. 1998;3.

For peer review only

For peer review only - http://bmjopen.bmj.com/site/about/guidelines.xhtml

31. Harris PA, Taylor R, Minor BL, Elliott V, Fernandez M, O'Neal L, et al. The REDCap consortium: Building an international community of software platform partners. Journal of Biomedical Informatics. 2019;95:103208.

32. Harris PA, Taylor R, Thielke R, Payne J, Gonzalez N, Conde JG. Research electronic data capture (REDCap)—A metadata-driven methodology and workflow process for providing translational research informatics support. Journal of Biomedical Informatics. 2009;42(2):377-81.

33. Braun V, Clarke V. To saturate or not to saturate? Questioning data saturation as a useful concept for thematic analysis and sample-size rationales. Qualitative Research in Sport, Exercise and Health. 2021;13(2):201-16.

34. QSR International Pty Ltd. Nvivo (Released in March 2020): https://www.qsrinternational.com/nvivo-qualitative-data-analysis-software/home; 2020. Available from: https://www.qsrinternational.com/nvivo-qualitative-data-analysis-software/home.

35. Braun V, Clarke V. Using thematic analysis in psychology. Qualitative Research in Psychology. 2006;3(2):77-101.

36. Byrne D. A worked example of Braun and Clarke's approach to reflexive thematic analysis. Quality & Quantity. 2021;56:1391-412.

37. Braun V, Clarke V. Reflecting on reflexive thematic analysis. Qualitative Research in Sport, Exercise and Health. 2019;11(4):589-97.

38. Clarke V, Braun V. Successful Qualitative Research: A Practical Guide for Beginners. 1 ed. London: SAGE Publications Ltd; 2013.

39. Olmos-Vega FM, Stalmeijer RE, Varpio L, Kahlke R. A practical guide to reflexivity in qualitative research: AMEE Guide No. 149. Medical Teacher. 2023;45(3):241-51.

40. Johnson JL, Adkins D, Chauvin S. A Review of the Quality Indicators of Rigor in Qualitative Research. Am J Pharm Educ. 2020;84(1):7120.

41. All Party Parliamentary Groups. Safe staffing: the impact of staffing shortages in maternity and neonatal care. Report of the baby loss and maternity all party parliamentary groups.; 2022.

42. Poprzeczny AJ, Stocking K, Showell M, Duffy JMN. Patient Decision Aids to Facilitate Shared Decision Making in Obstetrics and Gynecology: A Systematic Review and Meta-analysis. Obstetrics and gynecology. 2020;135(2):444-51.

43. Stacey D, Légaré F, Lewis K, Barry MJ, Bennett CL, Eden KB, et al. Decision aids for people facing health treatment or screening decisions.: Cochrane Database for Systematic Reviews; 2017.

44. Légaré F, Ratté S, Stacey D, Kryworuchko J, Gravel K, Graham ID, et al. Interventions for improving the adoption of shared decision making by healthcare professionals. Cochrane Database Syst Rev. 2010(5):Cd006732.

45. RCOG publishes resources to support pregnant women considering how to give birth [press release]. Royal College of Obstetricians and Gynaecologists2022.

46. Birthrights. IDECIDE - a new consent tool is on its way... 2020 [Available from: https://www.birthrights.org.uk/2020/01/30/idecide-a-new-consent-tool-is-on-its-way/.

47. Joseph-Williams N, Lloyd A, Edwards A, Stobbart L, Tomson D, Macphail S, et al. Implementing shared decision making in the NHS: lessons from the MAGIC programme. BMJ. 2017;357:j1744.

48. Elwyn G, Frosch D, Thomson R, Joseph-Williams N, Lloyd A, Kinnersley P, et al. Shared decision making: a model for clinical practice. Journal of general internal medicine. 2012;27(10):1361-7.

49. Chauvin P, Simonnot N, Douay C, Vanbiervliet F. Access to healthcare for the most vulnerable in a Europe in social crisis. 2014.

50. Knight M BK, Patel R, Shakespeare J, Kotnis R, Kenyon S, Kurinczuk JJ (Eds.) on behalf of MBRRACE-UK. Saving Lives, Improving Mothers' Care Core Report - Lessons learned to inform maternity care from the UK and Ireland Confidential Enquiries into Maternal Deaths and Morbidity 2018-20. Oxford: National Perinatal Epidemiology Unit, University of Oxford; 2022.

51. National Institute for Health and Care Excellence (NICE). Antenatal care. NICE guideline [NG201]. Evidence.: NICE; 2021. Available from: https://www.nice.org.uk/Guidance/NG201/evidence.

For peer review only

For peer review only - http://bmjopen.bmj.com/site/about/guidelines.xhtml

52. Lelie A. Decision-making in nephrology: shared decision making? Patient Educ Couns. 2000;39(1):81-9.

53. Begley K, Daly D, Panda S, Begley C. Shared decision-making in maternity care: Acknowledging and overcoming epistemic defeaters. J Eval Clin Pract. 2019;25(6):1113-20.

54. Kotaska A. Informed consent and refusal in obstetrics: A practical ethical guide. Birth. 2017;44(3):195-9.

55. Thompson A. Midwives' experiences of caring for women whose requests are not within clinical policies and guidelines. British Journal of Midwifery. 2013;21(8):564-70.

56. Jenkinson B, Kruske S, Stapleton H, Beckmann M, Reynolds M, Kildea S. Women's, midwives' and obstetricians' experiences of a structured process to document refusal of recommended maternity care. Women and Birth. 2016;29(6):531-41.

57. Akkad A, Jackson C, Kenyon S, Dixon-Woods M, Taub N, Habiba M. Informed consent for elective and emergency surgery: questionnaire study. BJOG: An International Journal of Obstetrics & Gynaecology. 2004;111(10):1133-8.

58. National Institute for Health and Care Excellence (NICE). NICE guidline [NG201] Antenatal care. Information provision. Evidence reviews underpinning recommendations 1.3.5, 1.3.7 to 1.3.11, 1.3.13, 1.3.14, 1.3.17 and 1.3.18: NICE; 2021. Available from: https://www.nice.org.uk/guidance/ng201/chapter/Recommendations.

59. López-Toribio M, Bravo P, Llupià A. Exploring women's experiences of participation in shared decision-making during childbirth: a qualitative study at a reference hospital in Spain. BMC Pregnancy and Childbirth. 2021;21(1):631.

60. Demetri A, Davies A, Bakhbakhi D, Ijaz S. Vaginal birth core information set: study protocol for a Delphi study to achieve a consensus on a "core information set" for vaginal birth. BMJ Open. 2023.

61. Merriel A. Development of a core information set for caesarean birth: COMET database.; 2023 [Available from: https://www.comet-initiative.org/Studies/Details/2599.

62. Merriel A. Developing a core information set for induction of labour: COMET Database; 2023 [Available from: https://www.comet-initiative.org/Studies/Details/2600.

63. Poprzeczny AJ, Stocking K, Showell M, Duffy JMN. Patient Decision Aids to Facilitate Shared Decision Making in Obstetrics and Gynecology: A Systematic Review and Meta-analysis. Obstet Gynecol. 2020;135(2):444-51.

For peer review only

For peer review only - http://bmjopen.bmj.com/site/about/guidelines.xhtml

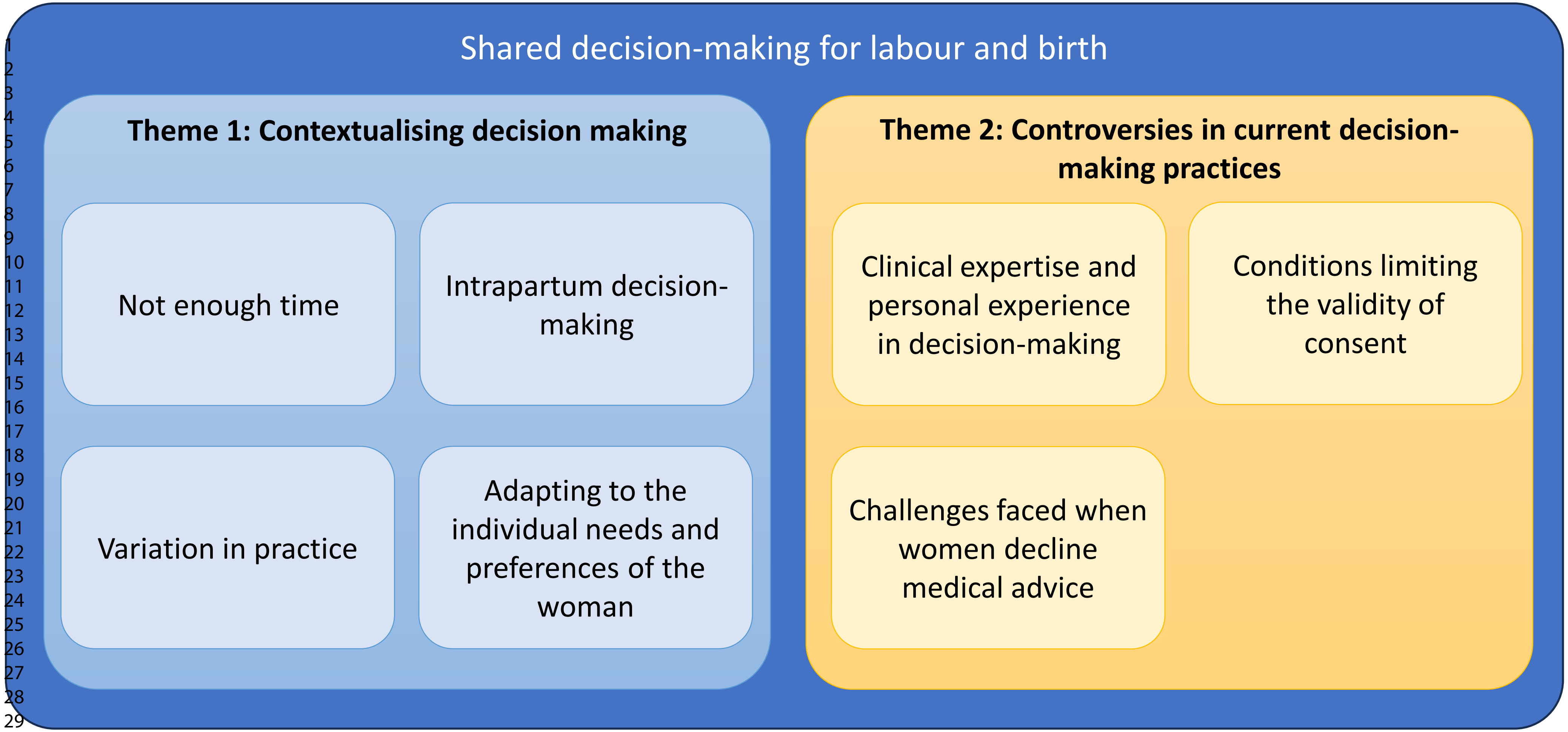
Shared decision-making for labour and birth
Theme 1: Contextualising decision making
Not enough time
Intrapartum decision-making
Variation in practice
Adapting to the individual needs and preferences of the woman
Theme 2: Controversies in current decision-making practices
Clinical expertise and personal experience in decision-making
Conditions limiting the validity of consent
Challenges faced when women decline medical advice

Supplementary table 1, Standards for Reporting Qualitative Research (SRQR) Checklist(1)

No.	Topic	Item
**Title and abstract**		
S1	**Title**	Page 1, Rows 2-3
S2	**Abstract**	Page 2, Rows 17-51
**Introduction**		
S3	**Problem formulation**	Pages 4-5, Rows 75-87
S4	**Purpose or research question**	Page 5, Rows 87-89
**Methods**		
S5	**Qualitative approach and research paradigm**	Page 6, Rows 106-108<br>Page 7, Rows 128-132
S6	**Researcher characteristics and reflexivity**	Page 5, Rows 96-104
S7	**Context**	Pages 6-7, Rows 119-126
S8	**Sampling strategy**	Page 6, Row 109-118<br>Page 7, Rows 139-143
S9	**Ethical issues pertaining to human subjects**	Page 21, Rows 375-377
S10	**Data collection methods**	Pages 6-7, Rows 119-126
S11	**Data collection instruments and technologies**	Pages 6-7, Rows 119-126
S12	**Units of study**	Page 8, Rows 144-150
S13	**Data processing**	Page 7, Rows 124-125.
S14	**Data analysis**	Page 7, Rows 127-133, and supplementary file, S3.
S15	**Techniques to enhance trustworthiness**	Page 7, Rows 134-143

For peer review only

Results/findings		
S16	**Synthesis and interpretation**	Pages 8-16, Rows 144-283.
S17	**Links to empirical data**	Pages 8-16, Rows 144-283. Supplementary files S4, S5.
**Discussion**		
S18	**Integration with prior work, implications, transferability, and contribution(s) to the field**	Pages 17 -19, Rows 284-340
S19	**Limitations**	Page 19, Rows 341-348
**Other**		
S20	**Conflicts of interest**	Page 20, Rows 363-364
S21	**Funding**	Page21, Rows 378-382

1. O'Brien BC, Harris IB, Beckman TJ, Reed DA, Cook DA. Standards for Reporting Qualitative Research: A Synthesis of Recommendations. Academic Medicine. 2014;89(9).

For peer review only

Shared decision making for labour and birth

Staff focus group questions

Intro: This study focusses on shared decision making. We're trying to understand your experiences of supporting women in their decision making.

We also want to find out about what you think is needed to optimise the support that you provide to women when making decisions.

Topic area 1:

Have you heard of shared decision making, what does it mean to you?

Prompt: exchange of information, listening, sharing, weighing, balancing

What do you think the important elements of decision making are?

Topic area 2:

Can you describe your personal experience of supporting women to make decisions?

Prompts: antenatally birth choices, ERCS vs VBAC, during labour, analgesia choices and pain management, instrumental delivery vs CS, Foetal blood sampling, birthing positions.

Topic area 3 :

Do you think women are well informed and prepared for the interventions, or medical care they might receive during labour and birth?

Appropriate expectations

Prompts: where do you think they get their information from, do you think the information they have is accurate and sufficient? What information do women receive about intrapartum interventions?

Do you think this affects their decision making ability?

For peer review only

Topic area 4:

How can we better support women to make decisions during labour and birth?

More training for health care professionals **,** More consultations, more antenatal classes, videos, leaflets

Topic area 5:

Which interventions, or decision choices do you think we should be focus on when practising shared decision making?

For example Which interventions are women least expecting? Least information. Most uncertainty, distress

Topic area 6:

Are there any interventions that should not involve shared decision making during labour?

Prompts: are you happy to participate in supporting shared decision making for all interventions? Life threatening emergencies are not usually expected to involve shared decision making – what is a life-threatening emergency to you?

For peer review only

Supplementary Table 3, A phased approach to reflexive thematic analysis (1, 2)

	Phase	Description of activities
1	Familiarisation with data	Immersion within the data was carried out independently (KH, AKD, AM). It was achieved by reading, and re-reading the data set and referring to the field notes associated with each focus group.
2	Generating initial codes	The dataset was coded independently by KH, AKD, and AM using succinct, shorthand descriptive labels.
3	Generating themes	Through a series of meetings, KH, AKD, and AM collaboratively explored initial themes using an inductive approach, whereby themes were directly linked to the data (3). Through this approach we hoped to directly represent the lived experiences of MHCPs', rather than by attempting to fit them onto pre-defined social constructs or frameworks.<br><br>In keeping with our experiential approach meaning and meaningfulness of themes was attributed to their relevance in answering the research question, and the significance participants attributed to these issues (4, 5, 6). Whilst we were also interested in the concept of theme frequency, and commonality, we also were aware that what is common is not necessarily meaningful or important(6).
4	Reviewing and identifying themes	A process of reading and re-reading themes, codes and data was carried out to reach a set of consistent, distinct, and coherent themes. Theme discussion, reflection and exploration occurred simultaneously.
5	Defining themes	The data set and coded data items were re-read to ensure that candidate themes functioned as meaningful interpretation of the data.
6	Write up	An illustrative narrative analysis of each theme was undertaken. A final review of theme name and order was undertaken to ensure the themes and key messages reflected the data, and answer the research question in a logical and clear manner.

1. Braun V, Clarke V. Reflecting on reflexive thematic analysis. Qualitative Research in Sport, Exercise and Health. 2019;11(4):589-97.
2. Byrne D. A worked example of Braun and Clarke's approach to reflexive thematic analysis. Quality & Quantity. 2021.

For peer review only

3. Braun V, Clarke V. Using thematic analysis in psychology. Qualitative Research in Psychology. 2006;3(2):77-101.
4. Byrne D. A worked example of Braun and Clarke's approach to reflexive thematic analysis. Quality & Quantity. 2021;56:1391-412.
5. Clarke V, Braun V. Thematic analysis. The Journal of Positive Psychology. 2017;12(3):297-8.
6. Braun V, Clarke V. Thematic analysis. APA handbook of research methods in psychology, Vol 2: Research designs: Quantitative, qualitative, neuropsychological, and biological. APA handbooks in psychology®. Washington, DC, US: American Psychological Association; 2012. p. 57-71.

For peer review only

**Supplementary Table 4. Theme 1: Contextualising decision-making**
Subtheme 1.1. Not enough time
lets explore that further, and what's concerning you, and what's led to that decision so far, so that we can make sure that it's the right decision for you. TO, P22.
So our discussion starts with "*Tell me about what you want your experience to be?...Tell me about what you're planning?"*...So it's very much a - their decision making can't happen without the information that I'm going to give them, but equally, I'm taking into account information they're giving me to help them come to a conclusion that works for them... BCMW, P4.
I keep coming back to time, maybe this is ....what I keep coming back to, but you know, it's time to process. Process that information, and then come to as [name] said, you know, what might not necessarily be what we think is the right decision, from our perspective, but when it comes to the patient, and you're bringing all that information together, they feel that's the right decision for them. TO, P22
the midwives that we all work with are incredibly stressed, underfunded, under great time pressures, and there are not enough of them to do the work that is required and the population is increasing and their workload is increasing. TO, P23.
And I feel like shared decision making is something that we all aspire to in situations where we as clinicians feel that there is enough time...TO, P24.
That's why women aren't given information, we have 20 minutes to do so many things...CMW, P11.
So great [name] that you've managed to find space for someone, in a quite a complex situation, but for the majority of women, it's a very superficial process... So we really need to improve that for, you know, for every woman. IMW, P3
you are very much trying to limit the consultation based on the time that you're given for that woman. So I would say that when I'm allowed, longer time with a woman, I would think it was a more informed decision that was going to come out of that because I have time to listen. CO, P17.
Subtheme 1.2: Intrapartum decision-making
There are times...that it isn't always possible to give them ...accommodate them having a discussion about something sometimes you do have to make more...more channelled decision making. BCMW, P5.
You have to we, we have to, and also the birth educators that we currently have in this country have to start having conversation from the very first antenatal class that they hold. BCMW, P6.
At the point where they're in... the process of the labour...that too much choice at that point is actually really derailing. And then I felt like I've left conversations thinking, why did I even? Why did I even do that to that poor woman? Like she's now on the edge to a really traumatic experience, because I've given her those choices and tried to say, look, there are other ways you can do X, Y, and Z. BCMW, P6.
How then are we expecting women to be ready to make decisions when they've actually not made a decision at all throughout the whole process of the nine months prior to that. So the whole time when they're meant to be training almost for the event of... Trying to make the shared decisions. We've not given them any training time. Instead, what you say is: "*Okay, at the point of birth, then you get choices*". But actually, at the point of birth, the choices go from nothing to a million and one choices. BCMW, P6.
So let's prepare them for the main options, and then train them to be fluid, you know, so that then they have a slightly more open minded, kind of coming into it. BCMW, P6
Subtheme 1.3: Variation in practice
I think there's just such a massive variety of sources of information that women receive and I don't think there's a huge amount of standardisation. TO, P22
Every single situation, every woman is different. Every doctor is different, every interaction is different. All you can do is keep honing your skills, practising and doing your best. There's no, I don't think there's one way that definitely isn't one way of doing it...A, P8.
But again, I think the whole consent thing is it is you tailor it to the patient... So it's very hard to say "*this is the way you should be imparting that information*". A, P9.

For peer review only

[we] will communicate with the same person in a different way, depending on the situation. A,P7.
We are all different people…we're better off getting a broad experience of seeing how different people do these things in order to work out actually what would work for our particular communication style or personality to try and keep things as, as shared and as broad as possible. TO, P22.
Sometimes women get less a whole lot less from me, than perhaps they should because I'm tired and rushed. A, P9.
So I think every single woman you tailor what you say differently. It's all according to like you say what, or how, or your perception of their understanding as well. IMW, P12.
There are days when you're better at it, then there are days when you think, "*Oh, God, I could have done that better*" CMW, P11.
I just think in general, communication is something that we bang on about all the time and you do it, you know…everyone's saying "*you know communication's key*", but actually, the communication isn't always there. IMW, P3
Skills around the actual conversation could be improved… you're making me think there's some teaching sessions we could be doing here. A, P9
I'd like a trainee to sit in clinic, and [name] trained in [place] as I did, and there was consultant there, who actually, came and sat in with you in clinic, and he sat there with you while you consulted, and by golly, your consultation style, improved, your feedback, etc… I think hands on direct, consultant, training like that, is really important. CO, 17.
Subtheme 1.4: Adapting to the individual needs and preferences of the woman
Whilst we want to give women this information, to try and empower them, and hopefully make things better, that I think there will be a group of patients who who will, they won't want that information, because they'll find it potentially very scary, or, you know that, but certainly, it might put some barriers up to accepting that information. TO, P22.
They don't realise that they can discuss that option. So I think when when, when you present them with an opportunity to discuss this, whatever problem they might have, um they're quite welcoming... I think sometimes they're quite surprised that that actually can happen, that they can discuss.... Whatever point they've they've come across with somebody. CMW, P2.
It's almost like continuing to give them permission that they can say what they feel, or they can say what they want or, you know, and then ...so there's that in the process of continuing to say, "*You have choice, this isn't prescription*" BCMW, P6
I think using an interpreter for people with a language barrier has a profound impact on trying to communicate in an emergency or even semi emergency situation. If I have someone on the labour ward who in any way might need a caesarean, sometimes in the middle of night, I find it quite useful to go in and go through a consent form with a translator in advance of doing a procedure because I think for those women communicating with them is so incredibly difficult... I think undoubtedly those women making intrapartum decision making is like the dreaded decision making because it's a difficult thing to communicate if both people have a shared first language, let alone with a language line and a phone interpreter and a possible partner. I think that's just like a perfect storm of issues.TO, P21.
You've got the whole range of the tertiary level educated patient who doesn't want us to do anything versus quite often someone who maybe left school after GCSEs...but you still have to provide both of those sets of patients with all the same information, but you have to then guide how you do that. And that's, that can be quite challenging. TO, P22.

For peer review only

**Supplementary Table 5. Theme 2: Controversies in current decision-making practices.**
Subtheme 2.1. Clinical expertise and personal experience in decision making
So what you would tend to do in that situation is probably stress the the downsides of having a general anaesthetic and talk about actually, you know, failed intubation ...So actually, we will manipulate that conversation based on us...thinking we actually probably do know the best thing for that patient. A, P8.
Yeah, exactly like a midwife, led unit midwife describing a breech in [place] where they do it quite frequently-ish, versus like a consultant who works in HCIB describing it at [place], that is a very different description that you will receive. TO, P21
We do all subconsciously do that, we select which bits of information we think the patient needs. A, P9.
Can you distinguish bias from experience? Or from or from teaching? I suppose, in that we're coming from a clinical viewpoint where we're...How do I put this? ...I'm trying to sort of say that it's not necessarily a biased opinion. Whereas I suppose what I'm trying to get at is that as a hopefully an experienced clinician, is it still bias? TO, P22
And so like if I don't want to induce a patient at 37 weeks for a pretty benign reason, but the patient is really keen to be induced, I will give them the figures for nicu admission, whereas if there's a patient who I want to induce, I might not necessarily tell them that same information. TO, P23.
Because we all know that, you know, with risks and percentages and risk ratios, etc, you can you can lean any decision to different ways. CO, P19.
I sort of feel like women are very coerced. ..And I feel like the information that's shared with women isn't neutral. They're scared into stuff. CMW, P12.
you acknowledge your bias and say, "*Well, obviously, I'm a consultant obstetrician, and I see, you know, a lot of high risk, and therefore I am biased*" CO, P18.
[the BRAIN app] is really good because it gives a really good balance and what are the risks, what are the benefits, what are the alternatives, what are the family's preferences. So it just it's a really good tool for facilitating those shared decisions, and looking at other people's perspectives as well. CMW, P2.
I do think sometimes putting numbers on things [by using absolute risk rather than relative risk] does help to give a kind of a more fair picture and allow people to make decisions that are maybe, well you know, just informs them and then they can make the decision they feel is right for them. TO, P24.
Subtheme 2.2. Conditions limiting validity of consent
I think in an emergency situation, I find it very difficult , because I think the consent process I currently go through seems like a bit of a sham… we go through this process of waving a consent form at them saying "*you and your baby going to die. If we don't do this*". TO, P21.
when you're trying to consent a labouring woman for an epidural, and she's screaming, "*just put it in*" at you that, you know, they don't take on board, we could tell them the risks were, you know, "*1 in 2 risk of death*" or something, at that point, they're not listening to you at all. A,P8.
We cannot say that a woman in labour is giving true consent, even for an epidural, when she has so much pain…She's so crippled and tired and, you know, fed up with everything, that she'll just agree to anything. IMW, P12.
I've never seen anyone try and do a decision making kind of conversation at the time of a shoulder dystocia, and I've also never come across a mum who has retrospectively said, "*I can't believe you didn't talk to me about that first*." TO, P24
"*Why was it five minutes before they took me around to theatre that somebody suddenly mentioned that I might end up having a caesarean section, why? When there were nine months when I could have been counselled about this*." TO, P21

We've got sort of risks of general anaesthesia, regional anaesthesia, we've got these lovely information cards from the OAA (Obstetric Anaesthetists' Association). A, P22
Subtheme 2.3. Challenges faced when women decline medical advice
She knew the risk, but she was absolutely clear what the risks were, what the implications could be what the outcome could be for her baby, but, that was the decision that she wanted. And it's it was so difficult. IMW, P3.
I think it is the fear of, of litigation, and that defensive practice, which is the overwhelming you know, feeling. I know, I've had some personal experiences around that. So that definitely does probably change the way I practice as a midwife, making me perhaps more overcautious… it's that kind of fear of, if something happens or goes wrong the responsibility then lies with you as the midwife, and the woman…will turn around and say, "*Well, that was something that you didn't do,*" Or "*if you'd have told me something differently, that wouldn't have happened*.". BCMW, P5.
They won't let you deliver that baby. And I find that always challenging and it takes maybe 12, 24 or 48 hours before you're allowed to do that. And then that baby obviously has, may have problems. And they're the ones I really struggle with…and it gets turned very much back against you as the medical professional saying, "*Why didn't you explain that this might happen*?" Even if it's been written in black and white. TO, P23
Yeah, it's, it's massive that and um what support networks are in there? Because at the end of the day, you still got another, you know, 50 women on your caseload that you've got to look after. IMW, P1
You need to talk about every option, not just the one you want them to do, and so you need to be really facetious about it.. It's a nightmare…CO, P18
If you give women too much information, you're just scare mongering, you know, if I say "*you've got this percentage chance and this percentage or whatever*". So it is difficult. CO, P19
The trace was pretty horrid... And she really did need to have a caesarean section, but she'd made the decision... And, and that's what we did. And I actually felt although it was, it wasn't a pleasant experience, it actually, for me, it was positive, because I know that we'd I'd worked with both with the couple as much as I possibly could. DSMW, P15
It's like women who decline induction, it's like, well, we'll tell you about the risks again, because you aren't doing what we've decided is the right thing to do from our perspective of, you know, recommendations. *"Remember, it's on you now*"…You know, and therefore, it's not shared decision making. BCMW, P6.
That might be because we've alienated people as well. So I think with with regards to pre birthing, and birthing outside of guidance. CO, P19.
They were they were quite bullish, actually in the hospital, they kept ringing her but she just turned the phone off in the end and said," I'm not going to speak to you, I need a day off from all of you. CMW, P11.
